# Precise regulation of cancer vaccine and immune checkpoint inhibitor synergy potentiates immunotherapy with reduced immune-related pneumonitis

**DOI:** 10.1016/j.mtbio.2026.102968

**Published:** 2026-02-23

**Authors:** Zhen Wang, Shuting Zuo, Xiaoyu Wan, Yan He, Xiaoman Jiang, Guanglin Fan, Qixiang Liu, Dan Shao, Qihui Liu, Yan Zhang

**Affiliations:** a Department of Breast Surgery, The Second Hospital of Jilin University, Changchun, 130041, PR China; b Department of Central Laboratory, Yuedong Hospital of the Third Affiliated Hospital of Sun Yat-sen University, Guangdong, 514700, PR China; c Department of Vascular Surgery, Jilin Province Qianwei Hospital, Changchun, 130041, PR China; d National Engineering Research Center for Tissue Restoraton and Reconstcton. South China University of Technology, Guangzhou, Guangdong, 510006, PR China; e Key Laboratory of Pathobiology, Ministry of Education, Nanomedicine and Translational Research Center, China-Japan Union Hospital of Jilin University, Changchun, 130033, PR China

**Keywords:** Anti-PD-1, Tumor vaccine, Checkpoint inhibitor pneumonitis, Breast cancer, Immunotherapy

## Abstract

The combination of cancer vaccine and an immune checkpoint inhibitor (ICI) function synergistically to induce effective antitumor immune responses. However, their clinical application is constrained by exacerbated immune-related adverse events (irAEs), notably checkpoint inhibitor-associated pneumonitis (CIP). To address this challenge, a peripheral lymphoid organ-targeted strategy was developed to spatiotemporally modulate T-cell responses through the co-localization of tumor vaccines and anti-PD1 (αPD1). This approach substantially reduced tumor growth and CIP severity by attenuating nonspecific T-cell infiltration in the lungs. In contrast, when tumor vaccines and αPD1 failed to precisely target the same T cell population, the enhanced therapeutic efficacy was at the cost of increased off-target CIP. As a consequence, combined tumor-specific T cells with PD1-blockade performed superior tumor-specific cytotoxicity and preferential tumor infiltration, further augmenting anti-tumor effects while minimizing CIP. These findings provide a homologous lymphoid organ-targeted paradigm that optimizes anti-tumor immune responses with reduced immune-related toxicities, offering a promising strategy for safer and more effective cancer immunotherapy.

## Highlights

• Tumor vaccines with αPD1 boost anti-tumor efficacy but raise CIP risk.
• CD8^+^ T cells are closely associated with the occurrence of CIP.
• Homologous targeted delivery strategy enables precise regulation of CD8^+^ T cells.
• Increasing tumor-specific T cells with PD1-blockade may reduce CIP risk.

## Graphical abstract

**Figure:**
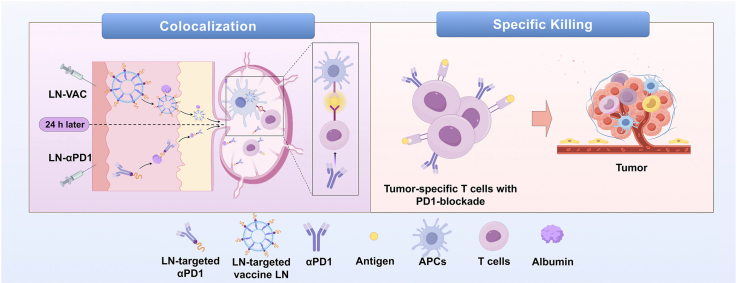

## 1 Introduction

The combination of ICIs and tumor vaccines significantly enhances overall survival and progression-free survival in patients considered incurable by conventional therapies, such as chemotherapy [[1], [2], [3], [4]]. This combinatorial strategy is especially promising for “cold tumors” (immunologically ignorant tumors), where vaccines boost immunogenicity and ICIs relieve immunosuppression, potentially converting them into “hot tumors”. Despite these substantial benefits, the number of patients deriving benefit remains limited, primarily due to exacerbated irAEs, notably checkpoint inhibitor-associated pneumonitis (CIP) [[5], [6], [7]].

So far, numerous ICIs and tumor vaccines have been developed as strategies to enhance anti-tumor effects [[8], [9], [10], [11], [12]]. For instance, some studies have focused on optimizing the antigen presenting cells (APCs) targeting efficiency of vaccines, such as improving the size, shape, charge, rigidity, and ligand modification of vaccines to enhance their delivery to APCs [[13], [14], [15], [16], [17]]. Most combined strategies involve multiple intravenous administrations of anti-PD1 (αPD1), PDL1 antibodies or CTLA-4 antibodies after vaccine treatment to amplify the anti-tumor effect. Although these pioneering studies aimed at enhancing anti-tumor effects are very encouraging, the improvements in CIP mitigation remain modest.

**Scheme 1:**
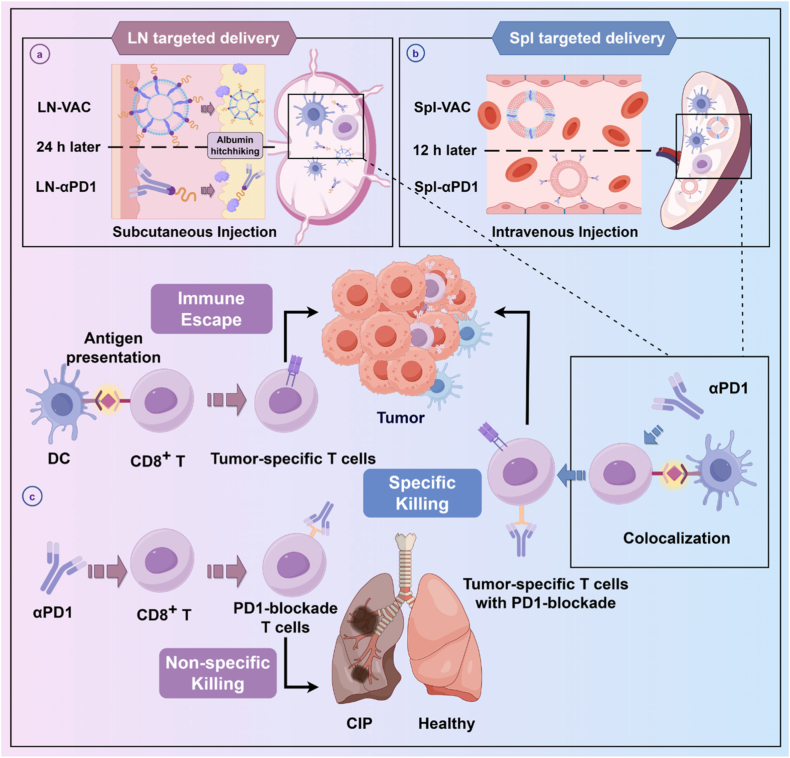
Schematic illustration of the spatial synergistic immunotherapy strategy combining tumor vaccines and αPD1. (a) Subcutaneous administration of modified tumor vaccines and αPD1 with optimized time intervals enables albumin hitchhiking for lymph node-targeted delivery. This promotes the generation of tumor-specific T cells with enhanced cytotoxicity and superior tumor infiltration, while significantly reducing the risk of CIP. (b) Similarly, a spleen-targeted co-delivery strategy achieves precise spatiotemporal co-localization of tumor vaccines and αPD1 on the same T-cell population, enabling fine-tuned immune modulation. (c) In contrast, conventional systemic αPD1 administration results in heterogeneous biodistribution, disrupting effective interactions among T cells, αPD1, and tumor vaccines. This impairs the generation of PD1-blockaded tumor-specific T cells, increases the likelihood of immune escape, and elevates the proportion of non-specific PD1-blockaded T cells, thereby heightening the risk of CIP.

Our findings demonstrate that αPD1-induced CIP is closely associated with non-tumor-specific CD8^+^ T cells with PD1-blockade. Accordingly, we hypothesize that enhancing the antigen-presenting efficiency of PD1-blockaded T cells could reducing the incidence of CIP. Herein, we developed a controllable strategy for co-localizing tumor vaccines and αPD1 to spatiotemporally regulate T cell responses (Scheme 1). Our results indicate that co-targeting tumor vaccines and αPD1 to the same peripheral lymphoid organ (draining lymph nodes or spleen) significantly reduced T cell infiltration in the lungs, particularly non-tumor-specific CD8^+^ T cells with PD1-blockade, leading to suppressed tumor growth and attenuated CIP severity. Conversely, when tumor vaccines and αPD1 failed to precisely target the same T cell populations, the enhanced therapeutic effects came at the cost of increased off-target CIP. Furthermore, when tumor-specific T cells were combined with PD1-blockade and reinfused into mice, they exhibited superior tumor-specific cytotoxicity and preferential tumor infiltration. These discoveries underscore the potential of spatiotemporally programmed immune modulation of T cells by tumor vaccines and immune checkpoint inhibitors to convert immune side effects into enhanced cancer treatment efficacy. This study provides an example for targeted therapy of homologous lymphoid organs and offers a promising strategy for safer and more effective cancer immunotherapy.

## 2 Methods

### 2.1 *In vivo* tumor models

#### 2.1.1 Tumor vaccine and αPD1 immunotherapy combination model

Female BALB/c mice (6–8 weeks old, n = 10) were subcutaneously inoculated with 1 × 10^6^ 4T1 cells. On day 6 post-inoculation, mice in the treatment group received a subcutaneous injection of 100 μL tumor cell membrane-derived vesicles (VAC), containing 500 μg tumor protein and 10 μg CpG. Starting on day 7, αPD1 (50 μL, 1 mg/mL; equivalent to 50 μg per mouse) was administered intravenously, with subsequent doses given every other day (days 9, 11, 13, and 15). Control groups received equivalent volumes of VAC alone, αPD1 alone, or phosphate-buffered saline (PBS). Tumor volume was measured every two days using a caliper and calculated as volume = (length × width^2^) × 0.5. Body weight was recorded twice daily. On day 18 post-inoculation, all mice were euthanized; tumors were excised, weighed, and processed for further analysis.

#### 2.1.2 Peripheral lymphoid organ-targeted delivery immunotherapy model

Female BALB/c mice (n = 10) were subcutaneously inoculated with 1 × 10^6^ 4T1 cells.

In the VAC+αPD1 treatment group, mice received a subcutaneous injection of VAC (100 μL, containing 500 μg tumor protein and 10 μg CpG) on day 7 post-inoculation. Starting on day 8, αPD1 (50 μL, 1 mg/mL; equivalent to 50 μg per mouse) was administered intravenously every two days for a total of four injections (days 8, 10, 12, and 14). Control mice received an equivalent volume of PBS.

In the lymph node-targeted delivery group, mice were administered LN-VAC (100 μL, containing 500 μg tumor protein and 10 μg CpG) subcutaneously on day 7, followed by subcutaneous administration of LN-αPD1 (50 μL, containing 50 μg αPD1) on days 8, 10, 12, and 14. The total doses of tumor antigen and αPD1 were kept identical between the targeted and non-targeted combination therapy groups. Tumor volume and body weight were measured every two days starting from day 7. On day 18, mice were euthanized, and lungs, bronchoalveolar lavage fluid (BALF), serum, and draining lymph nodes were harvested for further analysis.

In the spleen-targeted delivery model, the VAC+αPD1 group followed the same dosing schedule as described above, with αPD1 administered intravenously on days 8, 10, 12, and 14. Control mice received PBS intravenously at equivalent volumes. Spl-VAC was administered via tail vein injection on days 7, 10, and 13, with each dose containing 167 μg tumor protein and 3.3 μg CpG (total antigen and adjuvant exposure matched to that of the non-targeted group). Spl-αPD1 (66.7 μg αPD1) was administered intravenously 12 h after each Spl-VAC injection. Tumor volume and body weight were monitored every two days from day 7. On day 18, mice were euthanized, and lungs, BALF, and spleens were collected for subsequent analysis.

#### 2.1.3 Differential-targeted delivery models

Female BALB/c mice (n = 10 per group) were subcutaneously inoculated with 1 × 10^6^ 4T1 cells. On day 6 post-inoculation, mice were assigned to receive either LN-VAC (100 μL, containing 500 μg tumor protein and 10 μg CpG, s.c.) or Spl-VAC (100 μL, containing 500 μg tumor protein and 10 μg CpG, i.v.).

On day 7, mice in the LN-VAC group received Spl-αPD1 (50 μL, containing 50 μg αPD1, i.v.), whereas mice in the Spl-VAC group received LN-αPD1 (50 μL, containing 50 μg αPD1, s.c.). Additional doses of the respective targeted αPD1 formulations were administered every other day on days 9, 11, 13, and 15.

The VAC+αPD1 and control groups were treated as described in Section 2.1.1. Tumor volume and body weight were measured every two days as previously described. On day 18, mice were euthanized, and lungs, BALF, lymph nodes, and spleens were collected for subsequent analyses.

#### 2.1.4 T cell depletion with anti-CD8 antibody

To assess the role of CD8^+^ T cells in CIP, 4T1 tumor-bearing mice (n = 10) were established by subcutaneous inoculation of 1 × 10^6^ 4T1-OVA cells. Mice received VAC (100 μL, containing 500 μg tumor protein, s.c.) on days 7 and 11, followed by intravenous administration of αPD1 (100 μL, containing 100 μg) on days 8, 10, and 12. On days 6 and 9, anti-CD8 antibody (100 μg) was administered intravenously to deplete T cells. Control groups received isotype control (hamster IgG, 100 μg). On day 13, lungs were harvested, and one lobe was processed for hematoxylin and eosin (H&E) staining and flow cytometry.

#### 2.1.5 Adoptive transfer of engineered T cells

BMDCs were harvested from CD45.2 mice and, upon maturation, co-incubated with a tumor vaccine derived from 4T1 tumor cell membranes (1 mg/mL) for 24 h. Splenocytes were isolated from CD45.2^+^ OT1 mouse spleens and co-cultured with matured DCs for 6 h. CD8^+^ T cells were purified using magnetic bead sorting to obtain OVA-specific T cells (OVA-T). These OVA-T cells were incubated with αPD1 for 2 h to generate tumor-specific T cells with PD1-blockade (4T1&αPD1-T). Similarly, control T cells (T), OVA-specific T cells (4T1-T), and αPD1-treated T cells (αPD1-T) were prepared using the same protocol. On days 4, 8, 12, and 16 post-tumor inoculation, modified T cells (1 × 10^6^ cells) were adoptively transferred into CD45.1 mice bearing subcutaneous 4T1-OVA tumors established by injection of 1 × 10^6^ tumor cells, and tumor volume and weight were measured as previously described. The mice were euthanized on day 21.

Ethical approval for this study was obtained from South China University of Technology Animal Care and Use Committee (Approval No. 2023061) prior to the commencement of the research.

### 2.2 H&E staining for detection of lung inflammation in mice

To evaluate pneumonia incidence in animal experiments, lungs were harvested after mouse euthanasia, with part of the lung fixed and subjected to H&E staining. Histological examination was performed to identify the presence or absence of inflammatory infiltration indicative of CIP.

### 2.3 Inflammatory infiltration score

H&E-stained lung sections were evaluated for inflammatory infiltration by a blinded pathologist. The scoring was based on the area of inflammatory infiltration: 1 represents <20% infiltration area under the microscope, 2 represents 20-40% infiltration area, and 3 represents >40% infiltration area. The inflammatory infiltration score was calculated as: Score = Inflammatory infiltration area score × number of mice with CIP. Three random fields per section were analyzed under a light microscope.

### 2.4 Flow cytometry analysis

After collecting samples for H&E staining, the remaining lung tissue is digested to prepare for flow cytometry. After incubating with FcBlock (TruStain FcX™ anti-mouse CD16/32, Biolegend) to block non-specific binding, cells from the lungs were stained with fluorochrome-conjugated monoclonal antibodies at 4 °C for 30 min in the dark and then washed with PBS. Monoclonal antibodies are detailed in the supplementary information. Data were collected using a flow cytometer (FACS Aria, BD) and analyzed using FlowJo V10 software.

For data analysis, in treatment groups where pneumonia was observed, all data points were collected and analyzed from the CIP-positive mice. In groups with no observed pneumonia, a subset of mice was randomly selected to match the number of CIP-positive mice in the corresponding pneumonia-affected group, ensuring balanced sample sizes for statistical comparisons.

### 2.5 *In vivo* imaging system (IVIS) detection of drug distribution in peripheral lymphoid organs

#### 2.5.1 Biodistribution of LN-VAC and LN-αPD1 *in vivo*

LN-VAC and LN-αPD1 (FITC-labeled) were injected subcutaneously. At 24 h, mice were euthanized, and major organs (liver, spleen, kidneys, lungs, heart, and lymph nodes) were harvested. Fluorescence was assessed by IVIS.

#### 2.5.2 Biodistribution of Spl-VAC and Spl-αPD1 *in vivo*

Spl-VAC and Spl-αPD1 (FITC-labeled) were injected intravenously. At 2 h, major organs were harvested, and fluorescence was assessed by IVIS.

### 2.6 Detection of T cell-mediated cytotoxicity

BMDCs were extracted from OT1 mice and, after maturation, co-incubated with OVA257-264 peptide (1 mg/mL) for 24 h. Splenocytes were isolated from OT1 mouse spleens and co-incubated with DCs for 6 h. CD8^+^ T cells were then sorted using magnetic beads, yielding OVA antigen-specific T cells (OVA-T). These OVA-T cells were further co-incubated with αPD1 for 2 h to generate tumor-specific T cells with PD1-blockade (OVA&αPD1-T). A portion of spleen-derived cells, without undergoing antigen presentation, were directly sorted using magnetic beads and used as a control (T cells). These T cells were co-incubated with αPD1 for 2 h to prepare PD1-blockade T cells (αPD1-T). T cells, OVA-T, αPD1-T, and OVA&αPD1-T were each co-incubated with L929 and 4T1-OVA cells (5 × 10^3^ cells/well) for 24 h, and cell viability was assessed using an LDH assay.

### 2.7 Statistical analysis

Data are presented as mean ± standard deviation (SD). Statistical analyses were performed using GraphPad Prism 8 (GraphPad Software, Inc.). Data normality was assessed using the Shapiro-Wilk test prior to statistical comparisons. For datasets that satisfied the assumption of normality, one-way analysis of variance (ANOVA) followed by Tukey's post hoc test was used for multiple-group comparisons. Tumor growth curves were analyzed using two-way ANOVA with Bonferroni's correction for multiple comparisons. For datasets that did not satisfy normality assumptions, non-parametric statistical tests were applied. Specifically, the Mann-Whitney *U* test was used for comparisons between two groups, and the Kruskal-Wallis test followed by Dunn's post hoc test was used for comparisons among multiple groups. Significance was set at ∗*p* < 0.05, ∗∗*p* < 0.01, ∗∗∗*p* < 0.001; ns indicates no significance.

For further details regarding the materials used, please refer to the Supplementary methods and CTAT table.

## 3 Results

### 3.1 The increased risk of CIP in mice by the combined immunotherapy strategy of tumor vaccines and αPD1 is related to CD8^+^ T cells

**Fig. 1:**
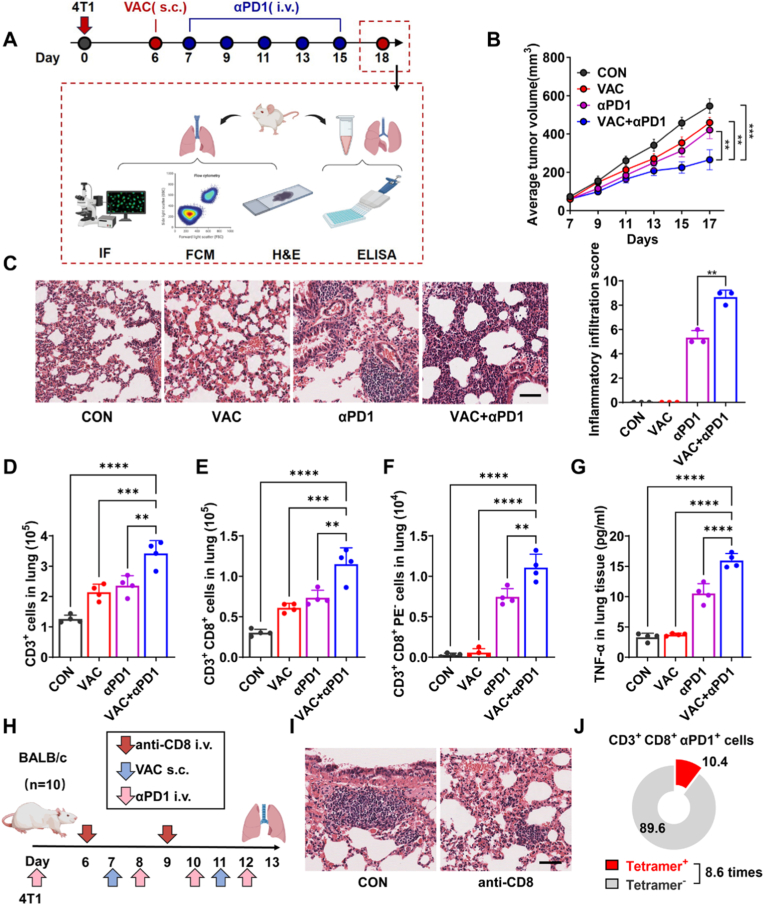
Anti-tumor Efficacy and Safety Profile of Tumor Vaccine Combined with αPD1 Immunotherapy. (A) Schematic illustration of the treatment schedule for combined immunotherapy in the 4T1 tumor-bearing mouse model. (B) Tumor growth curves in mice treated with PBS, tumor vaccine (VAC), αPD1, or VAC+αPD1 (n = 10). (C) Representative H&E-stained lung sections and quantified inflammatory infiltration scores indicating CIP severity (n = 3). Scale bar, 50 μm. (D-F) Flow cytometry quantification of (D) CD3^+^ T cells, (E) CD3^+^ CD8^+^ T cells and (F) CD3^+^ CD8^+^ PE^−^ T cells (n = 4). (G) Detection of TNF-α concentrations in lung tissue using ELISA (n = 4). (H) Schematic of the treatment schedule for T-cell depletion studies using anti-CD8 antibodies in the 4T1 model. (I) Representative H&E-stained lung sections. Scale bar, 50 μm. (J) Proportion of tumor-specific CD8^+^ T cells among PD1-blockade T cells in the lungs, assessed by flow cytometry (n = 4). Data are presented as mean ± SD. ∗∗*p* < 0.01, ∗∗∗∗*p* < 0.0001; ns, not significant.

Tumor membrane vaccines, which leverage tumor cell surface antigens, offer a promising approach for eliciting targeted immune responses [[18], [19], [20]]. In this study, we developed a tumor membrane vaccine derived from homologous tumor cells and evaluated its therapeutic efficacy and potential in combination αPD1 in a breast cancer model (Fig. 1A). Our results demonstrate that the combination therapy (VAC+αPD1) significantly suppressed tumor growth compared to vaccine alone (VAC) or αPD1 (Fig. 1B and Fig. S1A). However, the incidence and severity of CIP were increased in the VAC+αPD1 group compared to either VAC or αPD1 alone (Fig. 1C). Notably, no significant changes in mouse body weight were observed (Fig. S1B). Flow cytometry analysis of lung tissue revealed that the VAC+αPD1 group exhibited a significant increase in the populations of CD3^+^ T cells and CD3^+^ CD8^+^ T cells compared to VAC or αPD1 groups (Fig. 1D–E, Fig. S1C-E and Fig. S2). This finding was further corroborated by lung tissue section analysis (Fig. S3). Additionally, αPD1 treatment induced marked infiltration of PD1-blockade CD8^+^ T cells in the lung tissue (Fig. 1F). Compared to the VAC group, the VAC+αPD1 group showed elevated levels of TNF-α and IL-6 in both lung tissue and BALF, further indicating the occurrence of lung inflammation induced by the combination of tumor vaccine and αPD1 (Fig. 1G and Fig. S4).

To further investigate the role of CD8^+^ T cells in CIP, we utilized the 4T1 tumor-bearing mouse model. Seven days post-tumor inoculation, mice received VAC and αPD1 treatment. On days 6 and 9, anti-CD8 antibodies were administered to deplete CD8^+^ T cells, with untreated mice serving as the control group. On day 13, lungs were harvested and subjected to H&E staining (Fig. 1H). As shown in Fig. 1I, the severity of CIP was significantly reduced in the anti-CD8 antibody-treated group compared to the control group. Furthermore, in the lungs of CIP-affected mice from the control group, PD1-blocked CD8^+^ T cells were predominantly non-OVA antigen-specific. The abundance of these, tumor-nonspecific CD8^+^ T cells with PD1-blockade was 8.6-fold higher than that of tumor-specific CD8^+^ T cells (Fig. 1J and Fig. S5). These findings suggest that the infiltration of tumor-nonspecific CD8^+^ T cells with PD1-blockade, in the lung is a critical factor in the development of CIP.

### 3.2 Preparation and characterization of tumor vaccine and αPD1 targeted to peripheral lymphoid organs

**Fig. 2:**
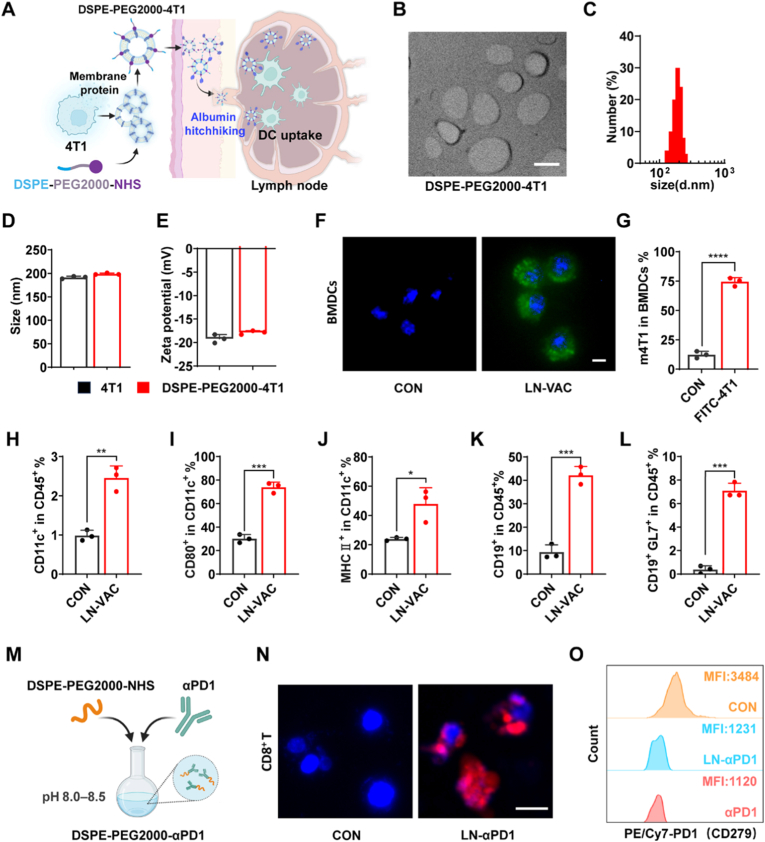
Schematic and Characterization of lymph node-targeted immunotherapies. (A) Schematic of LN-VAC synthesis. (B) TEM image of LN-VAC. Scale bar, 100 nm. (C) TEM measurement of LN-VAC particle size (n = 200 particles from at least three independent TEM images). (D-E) DLS characterization of LN-VAC showing (D) hydrodynamic diameter and (E) zeta potential (n = 3). (F) Immunofluorescence (IF) of FITC-labeled LN-VAC uptake by BMDCs after 24 h co-incubation. Blue, nuclei (DAPI); green, FITC-LN-VAC. Scale bar, 10 μm. (G) Flow cytometry detection of antigen binding in LN-VAC co-incubated with BMDCs for 24 h (n = 3). (H-L) Flow cytometry analysis of lymph node immune activation 7 days post-subcutaneous LN-VAC administration, showing (H) dendritic cell (DC) population changes, (I-J) DC maturation and activation markers, (K) B cell population changes and (L) germinative centers (n = 3). (M) Schematic illustration of LN-αPD1 preparation. (N) IF of FITC-labeled LN-αPD1 binding to CD8^+^ T cells after 2 h co-incubation. Blue, nuclei (DAPI); red, FITC-LN-αPD1. Scale bar, 10 μm. (O) Flow cytometry of PD1-blockade on T cells after 2 h co-incubation with LN-αPD1 (n = 3). Data are presented as mean ± SD, ∗*p* < 0.05, ∗∗*p* < 0.01, ∗∗∗*p* < 0.001, ∗∗∗∗*p* < 0.0001. (For interpretation of the references to colour in this figure legend, the reader is referred to the Web version of this article.)

To investigate whether spatial modulation of T cell responses through co-localized delivery of tumor vaccines and αPD1 could enhance antitumor efficacy while reducing the incidence of CIP, we first developed LN-VAC and LN-αPD1 for experimental evaluation. LN-VAC was prepared by conjugating DSPE-PEG2000-NHS with tumor cell membrane proteins extracted from 4T1 cells in a buffer solution (pH 8.0-8.5), followed by mixing with CpG (the mass ratio of tumor protein to CpG was 50:1) and incubating at 37 °C for 2 h. As an amphiphilic polymer, DSPE-PEG2000-NHS features a hydrophilic NHS terminus that forms covalent bonds with tumor cell membrane proteins. Following subcutaneous injection in mice, the hydrophobic DSPE moiety facilitates strong binding of the vaccine to albumin, enabling its transport to lymph nodes (Fig. 2A) [21,22]. As shown in Fig. 2B–E and Fig. S6, LN-VAC exhibited a negative zeta potential and a uniform particle size of approximately 180 nm. Additionally, bone marrow-derived dendritic cells (BMDCs) efficiently internalized LN-VAC *in vitro* (Fig. 2F–G and Fig. S7). In vivo, subcutaneous injection of LN-VAC for 7 days promoted dendritic cell maturation and germinal center formation in lymph nodes (Fig. 2H–L and Fig. S8). For LN-αPD1, conjugation of DSPE-PEG2000-NHS with αPD1 was performed (Fig. 2M). LN-αPD1 was labeled with FITC and co-incubated with CD8^+^ T cells for 2 h, with binding observed under fluorescence microscopy (Fig. 2N). Flow cytometry analysis confirmed that LN-αPD1 exhibited PD1 receptor blockade comparable to αPD1 (Fig. 2O) [23,24].

**Fig. 3:**
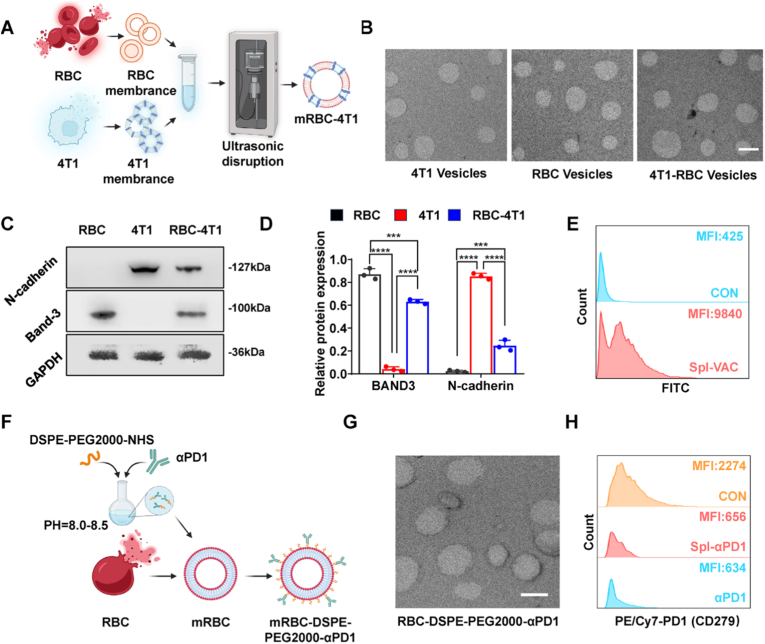
Preparation and Characterization of Spleen-Targeted immunotherapies. (A) Schematic of Spl-VAC synthesis. (B) TEM image of Spl-VAC. Scale bar, 100 nm. (C) Western blot of N-cadherin (4T1-specific) and Band-3 (RBC-specific) in 4T1 membranes, RBC membranes and 4T1-RBC hybrid membranes. (D) Quantitative analysis of Western blot (n = 3). (E) Flow cytometry analysis of uptake by BMDCs after 24 h co-incubation with Spl-VAC (n = 3). (F) Schematic illustration of Spl-αPD1 preparation. (G) TEM measurement of Spl-VAC and Spl-αPD1 particle sizes. Scale bar, 100 nm. (H) Flow cytometry of PD1-blockade on CD8^+^ T cells after 2 h co-incubation with Spl-αPD1 and αPD1 (n = 3). Data are presented as mean ± SD. ∗∗∗*p* < 0.001, ∗∗∗∗*p* < 0.0001.

Given the critical role of the spleen, the largest secondary lymphoid organ, in immune activation, we explored it as an alternative target for homologous delivery strategies. To enhance vaccine and αPD1 accumulation in the spleen, we utilized erythrocyte membranes [[25], [26], [27]]. Tumor cell membranes were thoroughly mixed with erythrocyte membranes (with a mass ratio of 1:20) under sonication and combining them with CpG (with a mass ratio of 50:1 for tumor cell membranes and CpG) to prepare hybrid vesicles (Fig. 3A), with an average particle size of approximately 210 nm and a negative charge (Fig. 3B and Fig. S9A–C). The hybrid membrane vesicles retained surface proteins from both vesicle types (Fig. 3C–D) and were efficiently taken up by DCs (Fig. S9D). Similarly, spleen-targeted αPD1 (Spl-αPD1) was prepared using erythrocyte membrane vesicles (Fig. 3F), with a particle size of approximately 200 nm and a negative charge (Fig. 3G and Fig. S9E). Both Spl-VAC and Spl-αPD1 exhibited good stability (Fig. S10 and Fig. S11). Notably, incorporating αPD1 into vesicles did not impair its ability to block PD1 receptors on CD8^+^ T cells compared to free αPD1 (Fig. 3H). These results demonstrate the successful development of tumor vaccines and αPD1 formulations targeted to peripheral lymphoid organs.

### 3.3 Spatial co-localization of tumor vaccine and αPD1 to modulate T cell responses

**Fig. 4:**
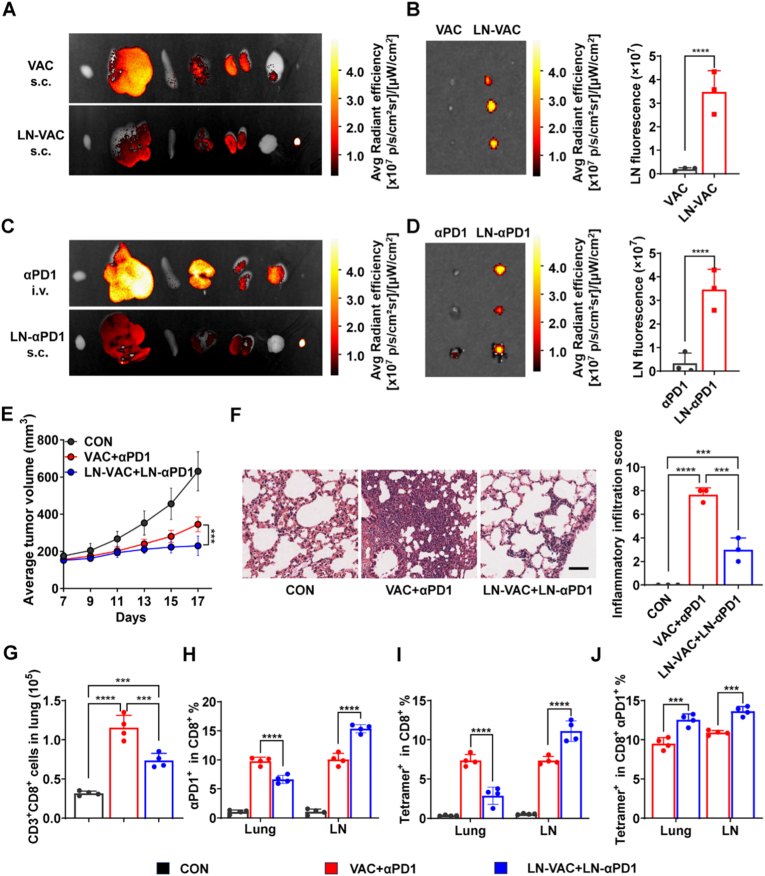
Anti-tumor efficacy and CIP assessment of LN-targeted delivery immunotherapy. (A) *In vivo* fluorescence imaging of FITC-labeled LN-VAC distribution 24 h post-subcutaneous injection (organs from left to right: heart, liver, spleen, lungs, kidneys, brain, lymph nodes) (n = 3). (B) Lymph node imaging and quantitative fluorescence analysis of LN-VAC accumulation (n = 3). (C) *In vivo* fluorescence imaging of FITC-labeled LN-αPD1 distribution 24 h post-subcutaneous injection (organs from left to right: heart, liver, spleen, lungs, kidneys, brain, lymph nodes) (n = 3). (D) Lymph node imaging and quantitative fluorescence analysis of LN-αPD1 accumulation (n = 3). (E) Tumor growth curves in 4T1 tumor-bearing mice treated with PBS, VAC+αPD1, or LN-VAC+LN-αPD1 (n = 10). (F) H&E-stained lung sections and quantified inflammatory infiltration scores indicating CIP severity (n = 3). Scale bar, 50 μm. (G) Flow cytometry quantification of CD3^+^ CD8^+^ T cells in lung tissues (n = 4). (H-I) Flow cytometry analysis of (H) PD1-blocked CD8^+^ T cells and (I) tumor-specific T cells as a proportion of total CD8^+^ T cells in lungs (n = 4). (J) Proportion of tumor-specific T cells among PD1-blocked CD8^+^ T cells in lungs (n = 4). Data are mean ± SD, ∗∗∗*p* < 0.001, ∗∗∗∗*p* < 0.0001.

To achieve spatial synchronization of tumor vaccine and αPD1 in modulating T cell responses, we first analyzed the biodistribution of LN-VAC and LN-αPD1 *in vivo*. FITC-labeled LN-VAC was administered via subcutaneous injection, and fluorescence imaging was performed using an *in vivo* imaging system (IVIS) over 72 h. As shown in Fig. 4A–B, Fig. S12 and Fig. S13A-B, compared to the VAC group (FITC-labeled 4T1 cell-derived tumor membrane proteins mixed with CpG), LN-VAC-FITC signals were predominantly concentrated in the draining lymph nodes (LNs) proximal to the injection site, with minimal signals detected in other organs such as the liver, kidney, lung, spleen, and heart. This demonstrates that LN-VAC specifically delivers antigens to LNs. Additionally, LN-VAC-FITC signals in LNs increased over time, reaching a peak at 24 h, decreasing to approximately 50% of maximum fluorescence by 48 h, and maintaining 30% of maximum fluorescence at 72 h (Fig. S13C–E).

A similar approach was employed for LN-αPD1, with results shown in Fig. 4C–D, Fig. S14 and Fig. S15A-B, demonstrating superior lymph node-targeting accumulation compared to free αPD1. Tissue fluorescence imaging revealed that LN-αPD1 fluorescence signals peaked at 12 h post-administration and decreased to approximately 20% of maximum fluorescence by 48 h (Fig. S15C–E). Given that antigen presentation typically requires 12 to 24 h during the initiation of immune responses, LN-αPD1 administration was performed 24 h after LN-VAC in subsequent studies to synchronize their effects on T cell modulation (Fig. S16) [[28], [29], [30]].

Next, the anti-tumor ability was investigated using a 4T1 tumor model (Fig. S17A), Mean tumor growth curves demonstrated that the lymph node-targeted group (LN-VAC+LN-αPD1) significantly inhibited tumor growth compared to the VAC+αPD1 group (Fig. 4E and Fig. S17B–C). No significant differences in body weight were observed among groups (Fig. S17D). As shown in Fig. 4F, the incidence and severity of pneumonitis were reduced in the LN-VAC+LN-αPD1 group compared to the VAC+αPD1 group. Further analysis by ELISA revealed decreased levels of IL-6 and TNF-α in lung tissue homogenates and BALF in the LN-VAC+LN-αPD1 group, indicating that the lymph node-targeted delivery strategy significantly attenuated lung inflammation (Fig. S18A–D). Lung tissue section analysis further revealed a significant reduction in CD8^+^ T cell infiltration in the targeted groups compared to the non-targeted VAC+αPD1 group (Fig. S18E–F). Flow cytometry analysis of immune responses in the lungs and lymph nodes showed that, compared to the VAC+αPD1 group, the LN-VAC+LN-αPD1 group exhibited significantly reduced numbers of CD8^+^ T cells, PD1-blocked CD8^+^ T cells, and antigen-specific CD8^+^ T cells in the lungs, potentially contributing to the reduced risk of CIP (Fig. 3G–I, Fig. S19-21). Correspondingly, the number of antigen-specific CD8^+^ T cells in draining lymph nodes was increased, suggesting enhanced antitumor activity (Fig. S22 and Fig. S23). Intriguingly, compared to the VAC+αPD1 group, the LN-VAC+LN-αPD1 group showed significantly elevated levels of antigen-specific CD8^+^ T cells with PD1-blockade in both the lungs and lymph nodes (Fig. 4J), highlighting the effective spatial synchronization of T cell responses achieved by the lymph node-targeted delivery strategy.

**Fig. 5:**
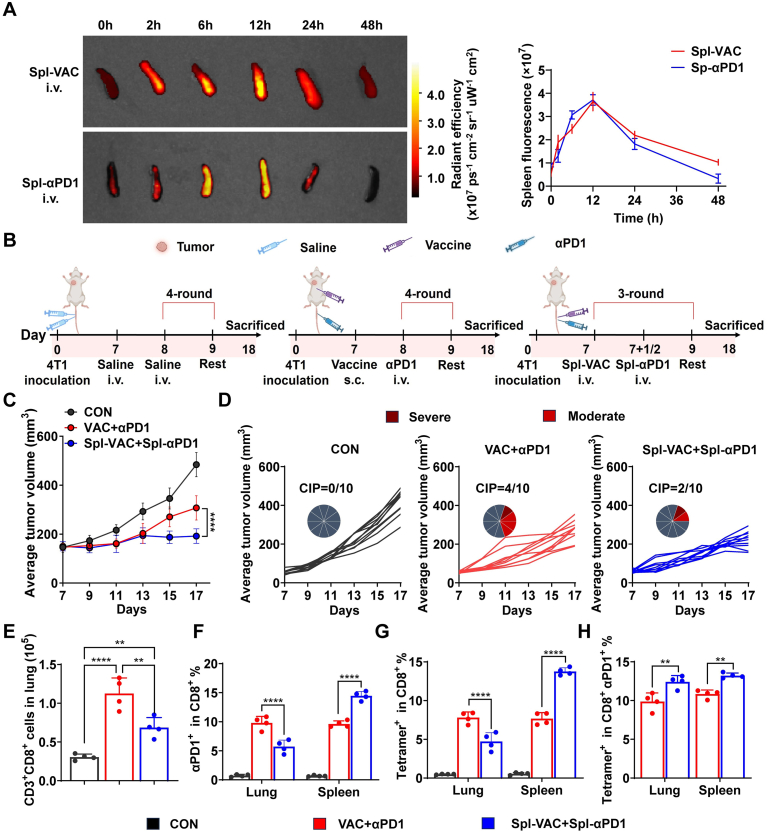
Anti-tumor efficacy and CIP assessment of Spl-targeted delivery immunotherapy. (A) IVIS detection of fluorescence intensity in spleens at 0, 2, 6, 12, 24, and 48 h post-intravenous injection of FITC-labeled Spl-VAC and Spl-αPD1, with corresponding statistical analysis of fluorescence intensity (n = 3). (B) Schematic of the treatment schedule for spleen-targeted delivery in 4T1 tumor-bearing mice. (C) Tumor volume changes in mice during treatment (n = 10). (D) Individual tumor growth curves for 4T1 tumor-bearing mice in CON group, VAC+αPD1 group and Spl-VAC+Spl-αPD1 group (n = 10),the red portion of the pie chart represents the number of mice with CIP, with the intensity of the red shade indicating the severity of CIP. (E) CD3^+^ CD8^+^ T cells in lung tissues (n = 4). (F-H) Flow cytometry analysis of (F) PD1-blocked CD8^+^ T cells, (G) tumor-specific T cells as a proportion of total CD8^+^ T cells in lungs and (H) proportion of tumor-specific T cells among PD1-blocked CD8^+^ T cells in lungs and spleen (n = 4). Data are mean ± SD, ∗∗*p* < 0.01, ∗∗∗∗*p* < 0.0001. (For interpretation of the references to colour in this figure legend, the reader is referred to the Web version of this article.)

We further evaluated the efficacy of a homologous targeting delivery strategy in a spleen-targeted model. The spleen-targeted efficiency of Spl-VAC and Spl-αPD1 was confirmed through IVIS analysis (Fig. S24–27), revealing an optimal administration interval of 12 h for the combination of Spl-VAC and Spl-αPD1 (Fig. 5A and Fig. S28). Subsequently, the antitumor efficacy of the spleen-targeted homologous delivery strategy was assessed in the 4T1 tumor model (Fig. 5B). As shown in Fig. 5C–D and Fig. S29, the Spl-VAC+Spl-αPD1 group exhibited significantly slower tumor growth compared to other groups, alongside a reduced CIP (Fig. S30–32). Moreover, compared to the VAC+αPD1 group, the Spl-VAC+Spl-αPD1 group showed significantly decreased numbers of CD8^+^ T cells, PD1-blockade CD8^+^ T cells, and antigen-specific CD8^+^ T cells in the lungs, with a corresponding increase in these cell populations in the spleen (Fig. 5E–H and Fig. S33-34). Similar to the lymph node-targeted homologous strategy, spleen-targeted treatment resulted in significantly elevated levels of antigen-specific CD8^+^ T cells with PD1-block in both the lungs and spleens, underscoring the feasibility of spatially modulating T cell responses through co-localized delivery of tumor vaccines and αPD1.

### 3.4 Immune effects of vaccine and αPD1 targeting different peripheral lymphoid organs

**Fig. 6:**
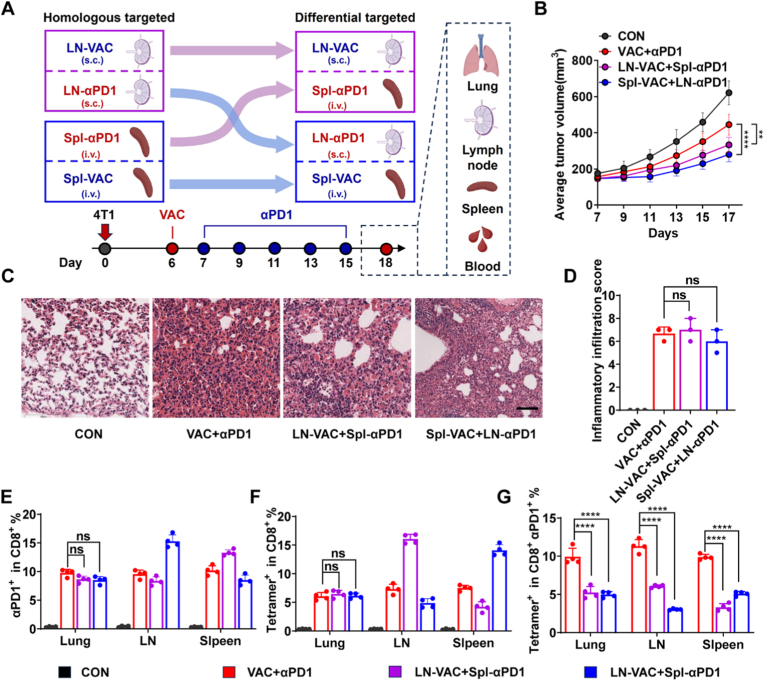
Immune Effects of Vaccine and αPD1 Targeting Different Peripheral Lymphoid Organs (A) Diagram of the targeted delivery strategy and schematic of the experimental timeline for the differential-targeting regimen in 4T1 tumor-bearing mice. In this study, homologous targeting refers to a delivery strategy in which both the tumor vaccine and PD-1 blockade are directed to the same peripheral lymphoid organ (e.g., LN-VAC administered subcutaneously (s.c.) together with LN-αPD1 (s.c.), or Spl-VAC administered intravenously (i.v.) together with Spl-αPD1 (i.v.)). In contrast, differential targeting refers to a strategy in which the tumor vaccine and PD-1 blockade are directed to different lymphoid organs (e.g., LN-VAC (s.c.) combined with Spl-αPD1 (i.v.), or Spl-VAC (i.v.) combined with LN-αPD1 (s.c.)). (B) Tumor growth curves in mice treated with PBS, VAC+αPD1, or differential targeting delivery combinations (LN-VAC+Spl-αPD1 or Spl-VAC+LN-αPD1) (n = 10). (C) H&E-stained lung tissue sections for detection of pulmonary inflammatory infiltration. Scale bar, 50 μm. (D) Quantified inflammatory infiltration scores indicating CIP severity (n = 3). (E-F) Flow cytometry analysis of (E) PD1-blocked CD8^+^ T cells and (F) tumor-specific T cells as a proportion of total CD8^+^ T cells in lungs, lymph nodes, and spleen (n = 4). (G) Proportion of tumor-specific T cells among PD1-blocked CD8^+^ T cells in lungs, lymph nodes, and spleen (n = 4). ∗∗*p* < 0.01, ∗∗∗*p* < 0.001, ∗∗∗∗*p* < 0.0001; ns, not significant.

To further evaluate the impact of targeting tumor vaccines and αPD1 to different peripheral lymphoid organs, we compared four treatment groups in the 4T1 mouse model: a control group receiving PBS (CON), non-targeted vaccine combined with free αPD1 (VAC+αPD1), lymph node-targeted vaccine combined with spleen-targeted αPD1 (LN-VAC+Spl-αPD1), and spleen-targeted vaccine combined with lymph node-targeted αPD1 (Spl-VAC + LN-αPD1) (Fig. 6A). In this study, “homologous targeting” refers to a delivery strategy in which both the tumor vaccine and PD1 blockade are directed to the same peripheral lymphoid organ, thereby aiming to promote spatiotemporal co-localization of antigen presentation and immune checkpoint modulation. In contrast, “differential targeting” denotes a strategy in which the tumor vaccine and PD1 blockade are directed to distinct lymphoid organs, resulting in spatial segregation of antigen priming and checkpoint inhibition. The results demonstrated that both the LN-VAC+Spl-αPD1 and Spl-VAC+LN-αPD1 groups exhibited superior antitumor efficacy compared to the VAC+αPD1 group (Fig. 6B and Fig. S35A-B). No significant changes in body weight were observed between the treatment groups (Fig. S35C).

To assess the incidence and severity of CIP, we performed H&E staining. As shown in Fig. 6C–D, no significant differences in inflammatory infiltration or CIP scores were observed among the treatment groups. Flow cytometry analysis revealed significant infiltration of CD8^+^ T cells in the lungs across all treatment groups, with no notable differences among them (Fig. S35D-F and Fig. S36). Lung tissue section analysis further confirmed marked CD8^+^ T cell infiltration in the treatment groups (Fig. S37). Although T cells in the targeted organs effectively interacted with αPD1 or the tumor vaccine, the differential targeting strategies did not reduce the proportion of PD1-blocked CD8^+^ T cells in the lungs and even decreased the proportion of tumor-specific CD8^+^ T cells with PD1-blockade (Fig. 6E–G, Fig. S38 and Fig. S39). Which may be closely associated with CIP development. Additionally, ELISA analysis revealed significantly elevated levels of IL-6 and TNF-α in lung and BALF across the treatment groups, further indicating the occurrence of inflammation (Fig. S40). Collectively, these findings suggest that targeting tumor vaccines and αPD1 to different peripheral lymphoid organs enhances antitumor efficacy compared to non-targeted vaccine and αPD1. However, these strategies do not reduce the incidence of immune-related adverse events.

### 3.5 Immune effects of reinfusion of tumor-specific T cells with PD1-blockade

**Fig. 7:**
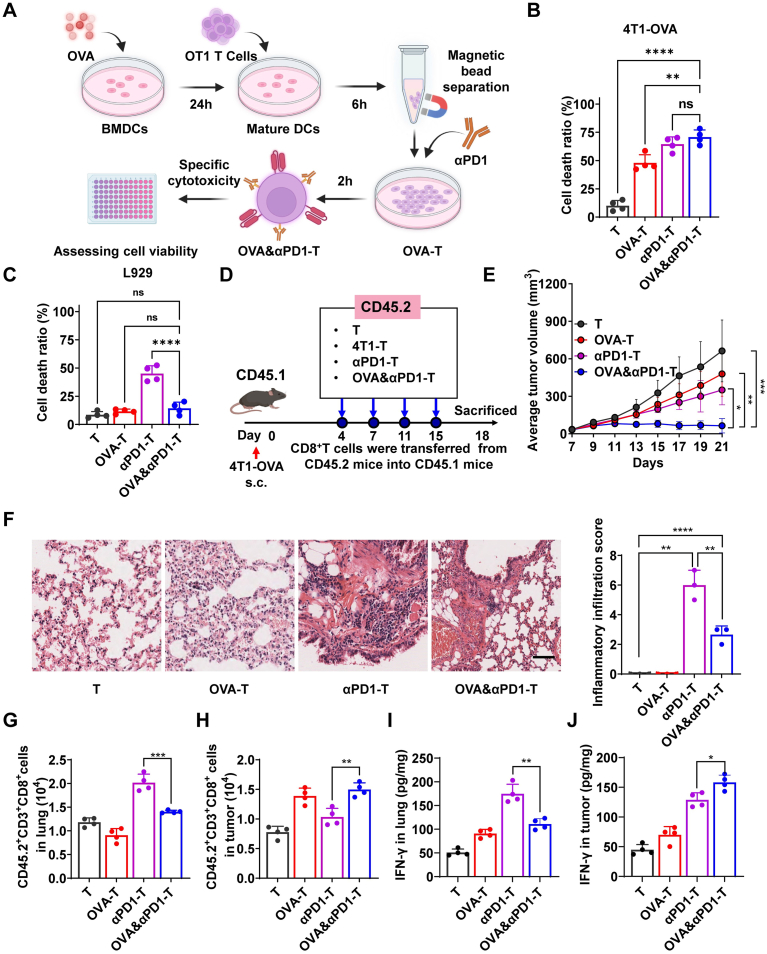
Tumor-Specific T Cells with PD1-blockade Enhance Anti-Tumor Efficacy and Reduce CIP in 4T1-OVA Tumor-Bearing Mice. (A) Schematic of *in vitro* cytotoxicity assay for homologous co-delivery of tumor vaccine and αPD1. (B-C) Cell death ratio of (B) 4T1-OVA cells and (C) L929 cells after co-incubation with T, OVA-T, αPD1-T, or OVA&αPD1-T cells, assessed by LDH assay (n = 4). (D) Schematic of T cell adoptive transfer experiment; CD8^+^ T cells from CD45.2 mice were isolated, modified *in vitro* to generate T, OVA-T, αPD1-T, and OVA&αPD1-T populations, and transferred into 4T1-OVA tumor-bearing CD45.1 mice. (E) Tumor growth curves in treated mice (n = 10). (F) H&E-stained lung sections and quantified inflammatory infiltration scores indicating CIP severity. Scale bar, 50 μm (n = 3). (G-H) Flow cytometry quantification of CD45.2^+^ CD3^+^ CD8^+^ T cells in (G) lungs and (H) tumors of CD45.1 mice (n = 4). (I-J) IFN-γ concentrations in (I) lungs and (J) tumors measured by ELISA (n = 4). Data are mean ± SD, ∗*p* < 0.05, ∗∗*p* < 0.01, ∗∗∗*p* < 0.001, ∗∗∗∗*p* < 0.0001; ns, not significant.

To elucidate the role of T cells in precisely modulating immune responses through a homologous targeting delivery strategy, we applied chimeric antigen receptor T-cell (CAR-T) principles to harvest and engineer T cells for tumor immunotherapy [31]. To investigate the cytotoxicity and specificity of *in vitro* modified T cells, BMDCs from OT1 mice were matured and co-incubated with OVA257-264 peptide (1 mg/mL) for 24 h. Splenocytes from OT1 mouse spleens were co-incubated with DCs for 6 h. CD8^+^ T cells were isolated using magnetic beads to obtain OVA-specific T cells (OVA-T), which were then co-incubated with αPD1 to generate PD1-blockade, tumor-specific T cells (OVA&αPD1-T). As a control, splenocytes not subjected to antigen presentation were directly isolated using magnetic beads (T cells) and co-incubated with αPD1 to prepare PD1-blockade T cells (αPD1-T) (Fig. 7A). Cytotoxicity assays were performed using 4T1-OVA cells (mimicking tumor cells) and L929 cells (mimicking autologous tissue cells). As shown in Fig. 7B–C, and Fig. S41, αPD1-T cells exhibited significantly enhanced cytotoxicity against both 4T1-OVA and L929 cells compared to unmodified T cells. Notably, OVA&αPD1-T cells displayed high cytotoxicity against 4T1-OVA cells but minimal toxicity toward L929 cells, suggesting that antigen presentation enhances the selective tumor-targeting capability of T cells. Additionally, compared to OVA&αPD1-T cells, tumor-specific OVA-T cells showed relatively lower cytotoxicity against 4T1-OVA cells, potentially due to immune escape mechanisms. IF confirmed the co-localization of engineered T cells with 4T1-OVA-mCherry cells, further validating their tumor specificity (Fig. S42A). These results collectively demonstrate that tumor-antigen-specific CD8^+^ T cells with PD1-blockade exhibit tumor-specific cytotoxic activity, whereas αPD1-T cells lack such specificity.

To further investigate the *in vivo* effects of tumor-specific T cells with PD1-blockade, CD8^+^ T cells were isolated from CD45.2^+^ OT1 mice, modified *ex vivo*, and adoptively transferred into tumor-bearing CD45.1 mice (Fig. 7D). Tumor volume and weight measurements revealed that mice receiving OVA&αPD1-T cell therapy exhibited significantly superior antitumor effects compared to other groups (Fig. 7E and Fig. S42B-C). IF and immunohistochemical analysis of lung tissue showed that CD8 expression (indicative of T cell infiltration) was significantly higher in the αPD1-T group compared to the OVA&αPD1-T group (Fig. S42D-E and Fig. S43). Furthermore, H&E staining was used to assess the incidence and severity of CIP. The OVA&αPD1-T group displayed a lower incidence and severity of CIP compared to the αPD1-T group (Fig. 7F), suggesting that converting PD1-blockade T cells into tumor-specific T cells reduces CIP and off-target effects. Flow cytometry analysis of CD8^+^ T cells in lungs and tumor tissues revealed distinct spatial distribution patterns (Fig. S44). Following reinfusion into CD45.1 mice, tumor-specific T cells predominantly infiltrated tumor tissues, whereas non-specific T cells showed no clear distribution preference (Fig. 7G–H). Notably, no significant differences were observed in the distribution of endogenous CD45.1 mouse T cells among groups (Fig. S45), indicating that CIP in mice is likely driven by adoptively transferred T cells. ELISA analysis of IFN-γ expression in lung and tumor tissues further confirmed spatial differences in expression. In tumors, IFN-γ levels were significantly higher in the OVA&αPD1-T group (Fig. 7I), while in the lungs, IFN-γ expression was higher in the αPD1-T group compared to other groups (Fig. 7J). Collectively, these findings indicate that tumor-specific T cells with PD1-blockade are a promising target for enhancing antitumor efficacy while minimizing nonspecific immune-related adverse events such as CIP.

## 4 Discussion

The integration of tumor vaccines with αPD1 therapy has demonstrated significant potential in enhancing cancer immunotherapy. Preclinical studies and early-phase clinical trials show that tumor vaccines induce tumor-specific T-cell responses, while αPD1 amplifies these responses by relieving checkpoint inhibition [10,11,[32], [33], [34]], improving overall and progression-free survival in otherwise refractory cancers. However, CIP remains a critical ICI-associated limitation, likely driven by immune hyperactivation and autoimmune attacks on healthy tissues [[5], [6], [7]], underscoring the need to elucidate CIP mechanisms in vaccine-αPD1 combinations. Consistent with previous reports [10,32], intravenous αPD1 after tumor vaccination suppressed subcutaneous tumor growth in mice but exacerbated CIP, accompanied by marked pulmonary T-cell infiltration, particularly CD8^+^ T cells. Prior work links irAEs such as CIP to nonspecific T-cell activation during systemic immunotherapy [6]; accordingly, CD8^+^ T-cell depletion markedly reduced lung inflammatory infiltration and CIP severity, consistent with clinical evidence implicating aberrant T-cell activation in irAEs [7,[35], [36], [37], [38]]. Notably, we identified a subset of lung CD8^+^ T cells activated by PD1 blockade yet unrelated to tumor recognition, suggesting that improving tumor antigen presentation to PD1-blocked T cells may help reduce CIP incidence.

Non-tumor-specific PD1-blocked CD8^+^ T cells may preferentially accumulate in the lung due to systemic expansion of activated bystander T cells and lung-specific recruitment/retention cues. PD-1 blockade broadens effector activation in inflammatory settings, increasing circulating activated CD8^+^ T cells. The lung's immune-reactive microenvironment, extensive capillary network, and inflammation-induced chemokines/adhesion molecules can further promote T-cell extravasation and retention, potentially contributing to CIP.

To test this hypothesis, we developed a targeted delivery immunotherapy strategy, involving the temporally and spatially controlled delivery of tumor vaccines and αPD1 to peripheral immune organs, precisely targeting the same T-cell subset to achieve programmed antigen presentation and PD1-blockade. Compared to previous immunotherapies combining tumor vaccines with systemic (intravenous) αPD1 administration [10,26], this co-localized delivery more effectively suppressed tumor growth while reducing CIP risk. Relative to systemic VAC+αPD1, the strategy increased antigen presentation to PD1-blocked CD8^+^ T cells in peripheral lymphoid organs and the lung, decreased pulmonary CD8^+^ T-cell infiltration, and lowered inflammatory cytokine levels, indicating enhanced tumor-specific immunity with fewer off-target immune effects. In contrast, delivering the vaccine and αPD1 to different lymphoid organs (e.g., LN-VAC+Spl-αPD1) improved antitumor efficacy but did not mitigate CIP, underscoring the importance of spatiotemporal programming. This programmed immunomodulation may also extend to adoptive cell therapy: *in vitro*-generated tumor-specific T cells with PD1 blockade (OVA&αPD1-T) showed stronger cytotoxicity against 4T1-OVA cells, greater tumor infiltration, higher intratumoral IFN-γ, and reduced lung CD8^+^ T cells. Overall, spatiotemporally programmed co-delivery of tumor vaccines and αPD1 enhances efficacy while lowering CIP by precisely coordinating antigen presentation and PD1 blockade in the same T-cell population.

Despite these encouraging findings, several limitations warrant consideration. The translational relevance of the murine CIP model and the conclusions drawn form a single tumor model (4T1/4T1-OVA) may be limited. In addition, as the therapeutic efficacy critically depends on the precise timing of LN-VAC and LN-αPD1 administration, the definitive contribution of stringent intranodal temporal coordination remains to be elucidated. To establish the generalizability and translational potential of this spatiotemporally programmed strategy, future studies should compare synchronized versus time-staggered administration regimens across diverse tumor models, including both solid and hematological malignancies. These efforts should be complemented by in-depth immune profiling, such as single-cell analyses and TCR repertoire sequencing, to dissect the underlying mechanisms, particularly the enhancement of antigen presentation to PD1-blocked T cells.

## 5 Conclusion

In this study, we demonstrate that spatiotemporally programmed co-delivery of tumor vaccines and αPD1 enables modulation of antigen presentation and PD1-blockade within the same T-cell population, thereby enhancing vaccine efficacy and reducing the risk of CIP. Our results identify PD1-blocked CD8^+^ T cells that do not recognize tumor antigen as the principal drivers of CIP. By targeting both the vaccine and αPD1 to the same peripheral lymphoid organs and applying time-staggered administration informed by their *in vivo* biodistribution, we achieved programmed control of antigen presentation and PD1 inhibition to selectively activate the same T-cell cohort. This strategy improved the efficiency of tumor antigen presentation to PD1-blockade T cells, augmented antitumor responses, and attenuated CIP. Validation experiments confirmed that immunomodulation of distinct T-cell populations by the vaccine and αPD1 does not reduce CIP risk. Moreover, the programmed immunomodulatory approach has important implications for CAR-T therapy: tumor-specific T cells subjected to PD1-blockade (OVA&αPD1-T cells) exhibited enhanced cytotoxicity and preferential tumor infiltration.

## Funding sources

This work was supported by 10.13039/501100001809National Natural Science Foundation of China [grant number 82503819]; 10.13039/501100013061Jilin Provincial Scientific and Technological Development Program [grant number YDZJ202501ZYTS011]; Zhongguancun Precision Medicine Foundation [grant number YJFH-YXKY-LC-04].

## CRediT authorship contribution statement

**Zhen Wang:** Project administration, Software, Writing – original draft. **Shuting Zuo:** Funding acquisition, Methodology, Writing – original draft. **Xiaoyu Wan:** Investigation, Project administration. **Yan He:** Project administration, Software, Writing – review & editing. **Xiaoman Jiang:** Data curation. **Guanglin Fan:** Data curation. **Qixiang Liu:** Data curation. **Dan Shao:** Methodology. **Qihui Liu:** Project administration, Writing – review & editing. **Yan Zhang:** Funding acquisition, Supervision, Writing – review & editing.

## Declaration of competing interest

The authors declare that they have no known competing financial interests or personal relationships that could have appeared to influence the work reported in this paper.

## Data Availability

Data will be made available on request.
