# Supplementary material for: Precise regulation of cancer vaccine and immune checkpoint inhibitor synergy potentiates immunotherapy with reduced immune-related pneumonitis
Source: Mater Today Bio. 2026 Feb 23;37:102968. doi: 10.1016/j.mtbio.2026.102968 (PMC12955687; doi:10.1016/j.mtbio.2026.102968)
Supplement: Multimedia component 1 [file mmc1.docx]

**Supplementary Material**

**Experimental Section**

## 1. Cell culture

4T1 cells, 4T1-OVA cells were cultured using DMEM with 10% fetal bovine serum at 37 ℃ with 5% CO_2_. To generate BMDCs, healthy BALB/c mice were euthanized, and their femurs were harvested, dissected, and surface-cleaned with 75% ethanol. Subsequently, the bone marrow cells were flushed from the bones using warm DMEM, then resuspended in BMDC growth medium supplemented with GM-CSF and IL-4 to induce differentiation. The cell were cultured for 6 days for subsequent use. To isolate CD8^+^ T cells, healthy BALB/c mice were euthanized, and their spleen were harvested, the spleen was placed in PBS, ground, and filtered through a 200-mesh sieve. The cells were resuspended in RBC lysis buffer at 4℃ for 5 minutes to fully lyse the RBCs. After centrifugation, the cells were collected and sorted for CD8^+^ T cells using the MojoSort kit. The sorting efficiency was evaluated by flow cytometry.

## 2. Animals

CD45.1 BALB/c mice，CD45.2 BALB/c and OT1 mice (6 to 8 weeks) were purchased from SLAC animal (Shanghai). Mice were treated under protocols approved by South China University of Technology Animal Careand Use Committee. All animals used in this experiment were raised in an SPF-grade animal laboratory center.

**3. Tumor cell membrane extraction**

Tumor cell membranes were derived from 4T1 or 4T1-OVA tumor cells, which were the same cell lines used for in vivo tumor inoculation. Collected tumor cells were washed twice with ice-cold PBS (pH 7.4) and then resuspended in hypotonic lysis buffer (10 mM Tris-HCl, pH 7.5; 10 mM NaCl; 1 mM MgCl₂) containing a protease inhibitor cocktail. After 15 min on ice, cells were disrupted by sonication (3–5 pulses, 10 s each, 30% amplitude) on ice. The homogenate was centrifuged at 1,000 × g for 10 min at 4°C to remove nuclei and debris. The supernatant was ultracentrifuged at 100,000 × g for 1 hour at 4°C to isolate the membrane fraction. The pellet was resuspended in solubilization buffer (50 mM Tris-HCl, pH 7.5, 150 mM NaCl, 1% Triton X-100, protease inhibitors), incubated for 30 min on ice, and clarified by centrifugation at 12,000 × g for 15 min at 4°C. Membrane proteins were quantified using a BCA assay and stored at -80°C [1].

**4. Preparation of lymph node-targeted vaccine (LN-VAC)**

Tumor cell membrane proteins (2 mg/mL in PBS, pH 7.4) were conjugated with DSPE-PEG2000-NHS (10 mg/ml in DMSO) at a 9:1 mass ratio. The mixture, with <5% DMSO (v/v), was incubated at room temperature for 2 hours with gentle shaking. The conjugate was purified by dialysis against PBS at 4°C for 24 hours with three buffer changes. Subsequently, CpG (100 µg, 1 mg/mL) was mixed with the DSPE-PEG2000-NHS-conjugated 4T1 membrane proteins (5 mg, 10 mg/mL) and incubated at 37°C for 2 hours prior to use, the vaccine dosage was based on the tumor cell membrane protein component, with each mouse receiving 500 μg [2].

**5. Preparation of lymph node-targeted αPD1 (LN-αPD1)**

DSPE-PEG2000-NHS and αPD1 were conjugated in 100 mM sodium bicarbonate buffer (pH 8.8) at a 10:1 mass ratio. The mixture was stirred on ice for 8 h, followed by dialysis (5 kDa MWCO) against PBS for 24 hours with three buffer changes. The protein concentration was measured using a BCA assay.

**6. RBC membrane isolation**

RBC membranes were isolated from whole blood collected from the same mice used in the corresponding animal experiments. Blood was collected in 1 mM EDTA. RBCs were lysed with 1.5 volumes of 1 mM EDTA, mixed for 5 min, and centrifuged at 4,000 rpm for 10 min at 4°C. The supernatant was centrifuged at 14,800 rpm for 20 min at 4°C to collect the membrane pellet. The pellet was resuspended in 100 nM EDTA, sonicated (60 Hz, 10 s), and centrifuged again at 14,800 rpm for 20 min. The pellet was washed twice with 100 nM EDTA, resuspended in 100 µL PBS, and quantified using a NanoDrop spectrophotometer [3].

**7. Preparation of spleen-targeted vaccine (Spl-VAC)**

The protein content of red blood cell membranes and 4T1 tumor cell membranes was determined using a BCA assay kit. The red blood cell membranes and tumor cell membranes were mixed at a 20:1 mass ratio, sonicated (60 Hz, 15 min) on ice, and incubated at 37°C for 30 min. CpG (the mass ratio of tumor cell membrane to CpG is 50:1) was added and incubated at 37°C for 2 hours prior to use [3, 4]. The mixture was then extruded through a 400 nm polycarbonate membrane.

**8. Preparation of spleen-targeted αPD1 (Spl-αPD1)**

RBC membranes were extruded through a 400 nm polycarbonate membrane and subsequently mixed with DSPE-PEG2000-αPD1, maintaining a mass ratio of RBC membrane proteins to αPD1 of 20:1. The mixture was stirred at 25°C for 2 hours to facilitate conjugation.

**9. Detection of physicochemical characterization**

Characterization of LN-VAC, LN-αPD1, Spl-VAC, and Spl-αPD1:

The morphology of LN-VAC, LN-αPD1, Spl-VAC, and Spl-αPD1 was observed using a JEM-2100F transmission electron microscope (JEOL, Japan). The hydrodynamic diameter and zeta potential of these formulations were measured using a Nano-ZS 90 Nanosizer (Malvern Instruments, UK). The stability of these formulations was assessed by dynamic light scattering (DLS).

To determine the drug loading of LN-αPD1:

After the completion of the conjugation reaction, the reaction mixture was collected and dialyzed using a 10 kDa MWCO dialysis membrane against PBS (pH 7.4) to remove low-molecular-weight impurities and unreacted components. The retained fraction was subsequently recovered, lyophilized, and weighed to obtain the dry mass of DSPE-PEG2000-αPD1. Drug loading was calculated as the mass ratio of αPD1 to DSPE-PEG2000-αPD1.

To determine the drug loading of Spl-αPD1:

Following the preparation of Spl-αPD1, the resulting suspension was collected and directly lyophilized to obtain the dry mass of RBC-DSPE-PEG2000-αPD1. Drug loading was then calculated as the mass ratio of αPD1 to RBC-DSPE-PEG2000-αPD1.

**10. Detection of red blood cell-tumor cell membrane fusion**

RBCs (DiL-labeled) and 4T1 membranes (FITC-labeled) were co-incubated with BMDCs for 6 hours, and uptake was visualized by fluorescence microscopy [5].

**11. Detection of tumor vaccine membrane-specific proteins**

RBC membranes and 4T1 tumor cell membranes were separately extracted to prepare Spl-VAC. Membrane proteins from RBC, 4T1 cells, and Spl-VAC were individually isolated, and WB analysis was performed to quantify the levels of N-cadherin (specific to tumor cells) and Band-3 (specific to RBCs) [5].

## 12. Stability Assessment

To evaluate the stability of the prepared formulations (including LN-VAC, Spl-VAC, LN-αPD1, and Spl-αPD1), freshly prepared samples were stored at 4°C in phosphate-buffered saline (PBS, pH 7.4) and subjected to dynamic assessment over a 7-day period. The hydrodynamic diameter and zeta potential were measured using a nanoparticle size analyzer via DLS on days 1, 3, 5, and 7, with each measurement conducted at 25°C in triplicate. For the release of CpG from LN-VAC and Spl-VAC, 1 mL of each formulation containing 1 mg of CpG was encapsulated in a dialysis bag (molecular weight cutoff: 5 kDa) and immersed in 10 mL of PBS (pH 7.4). At predetermined time points (days 1, 3, 5, and 7), 1 mL of the dialysis solution was collected as a sample for analysis, and the dialysis medium was replenished with fresh PBS. The CpG concentration in the samples was quantified using ELISA, with the cumulative release calculated as a percentage of the initial CpG content, and the experiments were performed in triplicate.

## 13. Detection of Vaccine Antigen Kinetics

To elucidate the antigen presentation processes of the vaccine within peripheral lymphoid organs (lymph nodes or spleen), LN-VAC (500 µg/mouse) was administered via subcutaneous injection, and mice were sacrificed 12-72 hours post-injection to assess antigen presentation by DCs and CD8^+^ T cells in the lymph nodes using flow cytometry. Similarly, to investigate the antigen kinetics of the spleen-targeted tumor vaccine, Spl-VAC (500 µg/mouse) was delivered via tail vein injection, and mice were sacrificed 12-72 hours post-injection to evaluate antigen presentation of the tumor vaccine by different immune cell subsets in the spleen using flow cytometry.

## Uptake of Targeted Tumor Vaccines in Peripheral Lymphoid Organs

To investigate whether the prepared targeted tumor vaccines are effectively captured by APCs following entry into lymph nodes, FITC-labeled LN-VAC formulations were administered via subcutaneous injection into female BALB/c mice. 12 hours post-injection, lymph node samples were collected and analyzed using flow cytometry to quantify FITC uptake by APCs and assess changes in immune cell subsets, with the non-targeted vaccine group serving as a control for comparison. Similarly, for the spleen-targeted tumor vaccine, FITC-labeled Spl-VAC formulations were delivered via intravenous injection into female BALB/c mice. 6 hours post-injection, spleen samples were harvested and evaluated by flow cytometry to quantify FITC uptake by APCs and monitor variations in immune cell subsets, again using the non-targeted vaccine group as a control for comparison.

## 15. Flow cytometry

**15.1. Detection of immune activation by LN-VAC in lymph nodes**

To assess LN-VAC immunostimulation, 500 µg LN-VAC was injected subcutaneously. After 7 days, axillary lymph nodes were harvested, processed into single-cell suspensions, and stained with APC-anti-CD45.2, V500-anti-CD11b, BV421-anti-CD3, PerCP-anti-CD11c, AF700-anti-MHCII, PE/Cy7-anti-CD4, BV711-anti-CD8, and AF488-anti-GL7. DC activation (CD80, MHCII) and B cell activation (GL7) were evaluated by flow cytometry.

**15.2. Verification of Spl-VAC uptake by DCs**

BMDCs were extracted from mice and differentiated into DCs. Spl-VAC was labeled with FITC and co-incubated with DCs in the dark for 24 hours. The fluorescence intensity of FITC in DCs was measured using flow cytometry.

**15.3. Verification of the blocking capacity of LN-αPD1 and Spl-αPD1 on T cells**

Primary T cells were isolated from mouse spleens and co-incubated with LN-αPD1 or Spl-αPD1 for 2 hours. The cells were then stained with PD1-PE antibody, and the degree of PD1 blockade on the T cell surface was evaluated by flow cytometry.

**16. IVIS detection of drug metabolism in peripheral lymphoid organs**

**16.1. *In vivo* lymph node metabolism**

LN-VAC and LN-αPD1 (FITC-labeled) were subcutaneously injected into BALB/c mice, with non-targeted VAC and αPD1 as controls. Lymph nodes were excised at 0, 6, 12, 24, 48 and 72 hours post-injection. Fluorescence intensity was measured using IVIS and quantified with Living Image software.

**16.2. *In vivo* spleen Metabolism**

Spl-VAC and Spl-αPD1 (FITC-labeled) were injected intravenously (n = 3/group), with non-targeted controls. Spleens were excised at 0, 2, 6, 12, 24, and 48 hours, and fluorescence was quantified by IVIS.

**17. T cell-tumor cell colocalization**

BMDCs were extracted from OT1 mice and differentiated into mature DCs, then co-incubated with OVA257–264 peptide (1 mg/mL) for 24 hours. Spleens from OT1 mice were processed into a single-cell suspension, subjected to red blood cell lysis, and stained with CFSE. Splenocytes (1×10⁷ cells) were co-incubated with DCs for 6 hours, followed by magnetic bead sorting to isolate OVA antigen-specific CD8^+^ T cells (OVA-T). αPD1 (10 μg/mL) was added to designated groups and incubated for 2 hours, defined as the OVA&αPD1-T group. Following the same protocol, T, OVA-T, and αPD1-T cells were sequentially prepared. Cells were collected, counted, and co-incubated with 4T1-OVA-mCherry cells for 2 hours. Each group (T, OVA-T, αPD1-T, OVA&αPD1-T) was then imaged using fluorescence microscopy.

**18. Detection of T cell-mediated cytotoxicity**

BMDCs were extracted from OT1 mice and, after maturation, co-incubated with OVA257–264 peptide (1 mg/mL) for 24 hours. Splenocytes were isolated from OT1 mouse spleens and co-incubated with DCs for 6 hours. CD8^+^ T cells were then sorted using magnetic beads, yielding OVA antigen-specific T cells (OVA-T). These OVA-T cells were further co-incubated with αPD1 for 2 hours to generate tumor-specific PD1-blockade T cells (OVA&αPD1-T). A portion of spleen-derived cells, without undergoing antigen presentation, were directly sorted using magnetic beads and used as a control (T cells). These T cells were co-incubated with αPD1 for 2 hours to prepare PD1-blocked T cells (αPD1-T). T cells, OVA-T, αPD1-T, and OVA&αPD1-T were each co-incubated with L929 and 4T1-OVA cells (5 × 10³ cells/well) for 24 hours, and the MTT assay was used to detect cell death rate [6, 7].

**19. Immunohistochemical detection of CD8 expression in mouse lungs**

To perform immunohistochemical detection of CD8 expression in mouse lungs, lung tissues were harvested from euthanized mice and fixed in 10% neutral buffered formalin for 24 hours. The tissues were then dehydrated through a graded ethanol series (70%, 80%, 95%, 100%) and embedded in paraffin. Sections (4–5 μm thick) were cut using a microtome and mounted on glass slides. Slides were deparaffinized in xylene and rehydrated through a descending ethanol series (100%, 95%, 70%) to water. Antigen retrieval was performed by heating the slides in a citrate buffer (pH 6.0) at 95°C for 20 minutes, followed by cooling to room temperature. Endogenous peroxidase activity was blocked with 3% hydrogen peroxide for 10 minutes. Non-specific binding was minimized by incubating sections with 5% bovine serum albumin (BSA) in phosphate-buffered saline (PBS) for 30 minutes. Sections were then incubated with a primary anti-CD8 antibody (rabbit anti-mouse CD8, 1:200 dilution) overnight at 4°C in a humidified chamber. After washing with PBS, slides were incubated with a horseradish peroxidase (HRP)-conjugated secondary antibody for 1 hour at room temperature. The signal was developed using 3,3'-diaminobenzidine (DAB) substrate for 5-10 minutes, and sections were counterstained with hematoxylin for 1 minute. Slides were dehydrated through an ascending ethanol series, cleared in xylene, and coverslipped with mounting medium. CD8 expression was visualized and quantified under a light microscope, with images captured from three random fields per section for analysis.

**20. ELISA detection**

To detect IL-6 and TNF-α levels in BALF and lung tissue fluid using ELISA, mice were euthanized, and BALF was collected by cannulating the trachea and flushing the lungs with 1 mL of sterile phosphate-buffered saline (PBS) three times, pooling the recovered fluid, and centrifuging at 500 × g for 5 minutes at 4°C to obtain the supernatant. The lung tissue was placed in a pre-chilled 1.5 mL or 2 mL microcentrifuge tube containing ice-cold homogenization buffer. The tissue was thoroughly homogenized at 4°C until fully disrupted. The homogenate was transferred to a new microcentrifuge tube and centrifuged at 12,000–15,000 × g for 10-15 minutes at 4°C to pellet cellular debris and insoluble material. The supernatant (lung tissue fluid) was carefully collected, transferred to a new microcentrifuge tube, and labeled. ELISA was performed using BioLegend kits specific for mouse IL-6 and TNF-α. Briefly, 96-well plates were coated with capture antibodies diluted in coating buffer and incubated overnight at 4°C. Plates were washed four times with wash buffer (PBS with 0.05% Tween-20) and blocked with assay diluent (1% BSA in PBS) for 1 hour at room temperature. After washing, 100 µL of BALF or lung tissue fluid samples and standards were added to wells in duplicate and incubated for 2 hours at room temperature with gentle shaking. Plates were washed, and detection antibodies were added for 1 hour, followed by another wash and incubation with avidin-HRP for 30 minutes. Absorbance was measured at 450 nm using a microplate reader, and cytokine concentrations were calculated based on standard curves generated from known concentrations of IL-6 and TNF-α standards.

**References**

[1] Gan J, Du G, He C, et al. Tumor cell membrane enveloped aluminum phosphate nanoparticles for enhanced cancer vaccination. J. J Control Release. Oct 10 **2020**;326:297-309. https://doi.org/10.1016/j.jconrel.2020.07.008

[2] Qin H, Zhao R, Qin Y, et al. Development of a Cancer Vaccine Using In Vivo Click-Chemistry-Mediated Active Lymph Node Accumulation for Improved Immunotherapy. J. Adv Mater. May **2021**;33(20):e2006007.https://doi.org/10.1002/adma.202006007

[3] Han X, Shen S, Fan Q, et al. Red blood cell-derived nanoerythrosome for antigen delivery with enhanced cancer immunotherapy. J. Sci Adv. Oct **2019**;5(10):eaaw6870. https://doi.org/10.1126/sciadv.aaw6870

[4] Jiang Q, Liu Y, Guo R, et al. Erythrocyte-cancer hybrid membrane-camouflaged melanin nanoparticles for enhancing photothermal therapy efficacy in tumors. J. Biomaterials. Feb **2019**;192:292-308. https://doi.org/10.1016/j.biomaterials.2018.11.021

[5] Wang D, Dong H, Li M, et al. Erythrocyte-Cancer Hybrid Membrane Camouflaged Hollow Copper Sulfide Nanoparticles for Prolonged Circulation Life and Homotypic-Targeting Photothermal/Chemotherapy of Melanoma. J. ACS Nano. Jun 26 **2018**;12(6):5241-5252. https://doi.org/10.1021/acsnano.7b08355

[6] Kansal R, Richardson N, Neeli I, et al. Sustained B cell depletion by CD19-targeted CAR T cells is a highly effective treatment for murine lupus. J. Sci Transl Med. Mar 6 **2019**;11(482). https://doi.org/10.1126/scitranslmed.aav1648

[7] Yang X, Zhu X, Sheng J, et al. RNF213 promotes Treg cell differentiation by facilitating K63-linked ubiquitination and nuclear translocation of FOXO1. J. Nat Commun. Jul 16 **2024**;15(1):5961. https://doi.org/10.1038/s41467-024-50392-z

**21. CTAT table**

| Reagent | Supplier | Cat no. |
| --- | --- | --- |
| Red Blood Cell Lysis Buffer | Beyotime | C3702 |
| Collagenase Type IV | Sigma-Aldrich | C5138 |
| Deoxyribonuclease Ⅰ | Sigma-Aldrich | DN25 |
| ELISA MAX^TM^Deeluxe Set Mouse TNF-α | BioLegend | 430904 |
| ELISA MAX^TM^Deeluxe Set Mouse IL-6 | BioLegend | 431304 |
| ELISA MAX^TM^Deeluxe Set Mouse IFN-γ | BioLegend | 430804 |
| Mouse CpG igodeoxynucleotide (CpG-ODN) ELISA Kit | MyBioSource | MBS725299 |
| MojoSort™ Mouse CD8 T Cell Isolation Kit | BioLegend | 480007 |
| N Cadherin Antibody | Affinity | AF5239 |
| Band 3 Polyclonal Antibody | Thermo | PA5-80030 |
| TruStain FcX™ (anti-mouse CD16/32) Antibody | Biolegend | 101320 |
| FITC anti-mouse NK 1.1 Antibody | Biolegend | 108706 |
| PE/Cy7 - F4/80 Antibody | Biolegend | 123114 |
| BV 421 - CD3 Antibody | Biolegend | 100228 |
| BV 711 - CD8a Antibody | Biolegend | 100748 |
| BV 785 - F4/80 Antibody | Biolegend | 123141 |
| Anti-mouse Ly6C/Ly6G Antibody | Bioxcell | BE0320 |
| V500 -CD11b Antibody | BD | 562127 |
| BUV563 - CD4 Antibody | BD | 565709 |
| APC/Cy7-CD4 Antibody | Biolegend | 100414 |
| PE-OVA257-264 Antibody | Invitrogen | 12-5743-81 |
| APC/Cy7-CD45.2 Antibody | Biolegend | 109824 |
| APC-CD45.1 Antibody | Biolegend | 110714 |
| FITC-F4/80 Antibody | Invitrogen | 11-4801-82 |
| PE/Cy7-CD279 Antibody | Biolegend | 109110 |
| AF700-I-A/I-E Antibody | Biolegend | 107622 |
| Name | Supplier | Sequence (5’ to 3’) |
| CpG ODN 1826 | Sangon Biotech | TCC CTG ACG TTC CTG ACG TT |

**Table S1.** Drug Loading Capacity.

| **Targeted αPD1 formulations** |  | **αPD1 (%)** |
| --- | --- | --- |
| DSPE-PEG2000-αPD1  （LN-αPD1） |  | 15.62±1.35 |
| RBC-DSPE-PEG2000-αPD1  （Spl-αPD1） |  | 11.9±1.23 |

**Results**


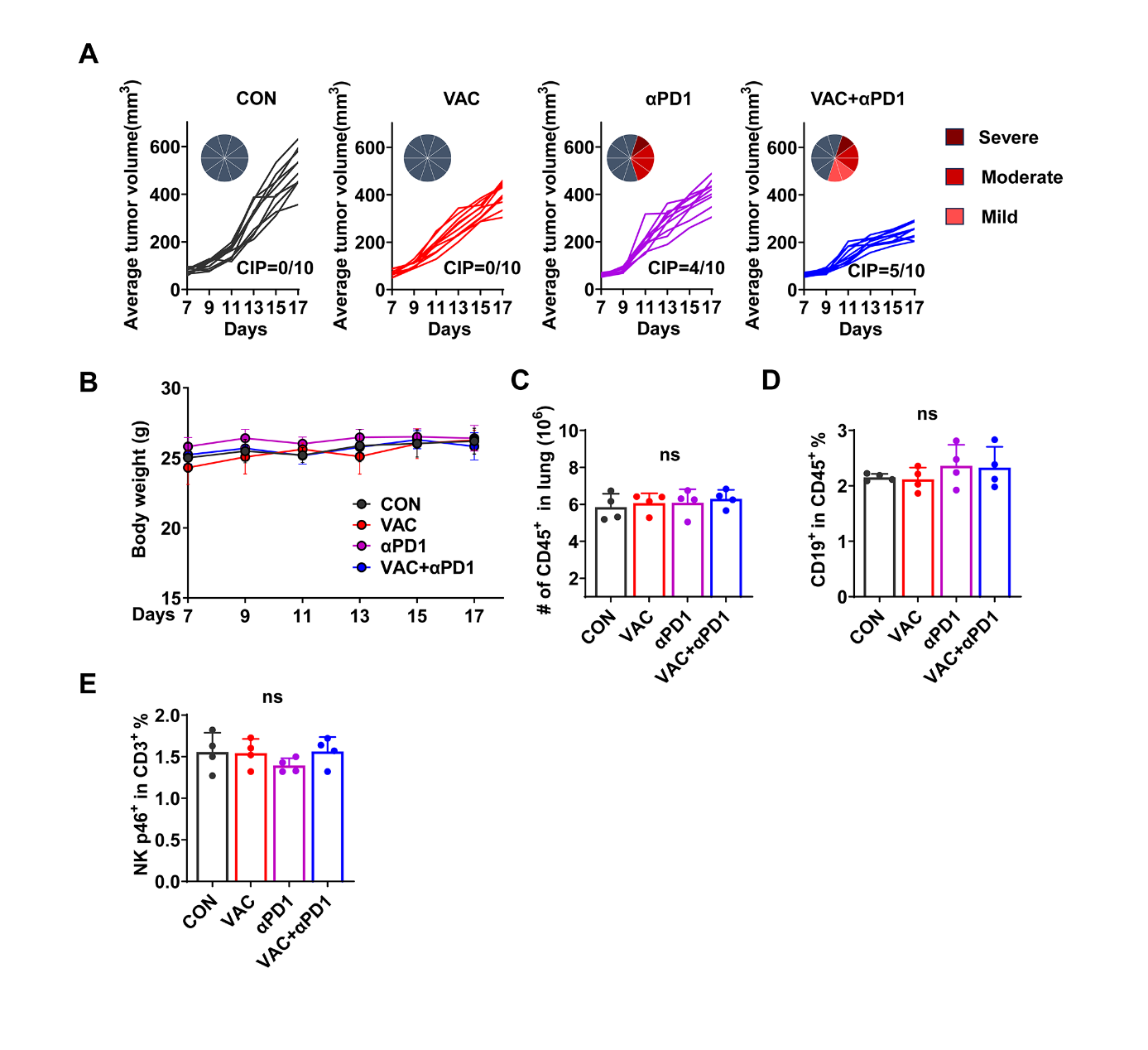


**Figure S1: Anti-Tumor Efficacy of Tumor Vaccine Combined with αPD1 Immunotherapy.** (A) Individual tumor growth curves for 4T1 tumor-bearing mice treated with PBS (CON), tumor vaccine (VAC), αPD1, or VAC+αPD1 (n = 10)，the red portion of the pie chart represents the number of mice with CIP, with the intensity of the red shade indicating the severity of CIP. (B) Body weight changes across treatment groups during the study period (n = 10). (C) Flow cytometry quantification of CD45^+^ T cells (n = 4). (D–E) Flow cytometry analysis of (D) B cells and (E) NK cells in lung tissues from different treatment groups (n = 4). Data are presented as mean ± SD, ns, not significant.


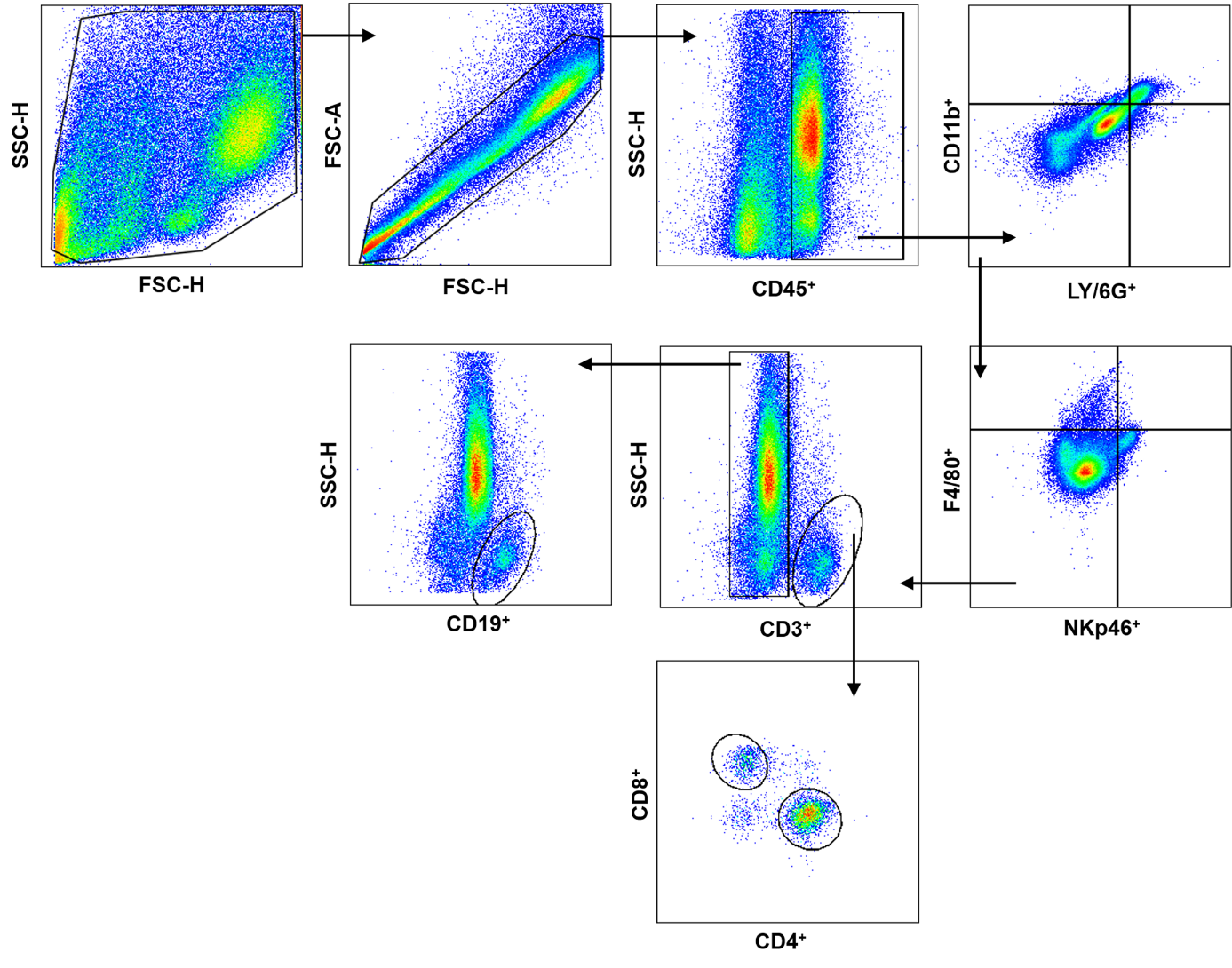
**Figure S2: Flow Cytometry Gating Strategy for Pneumonia Investigation Experiment.**


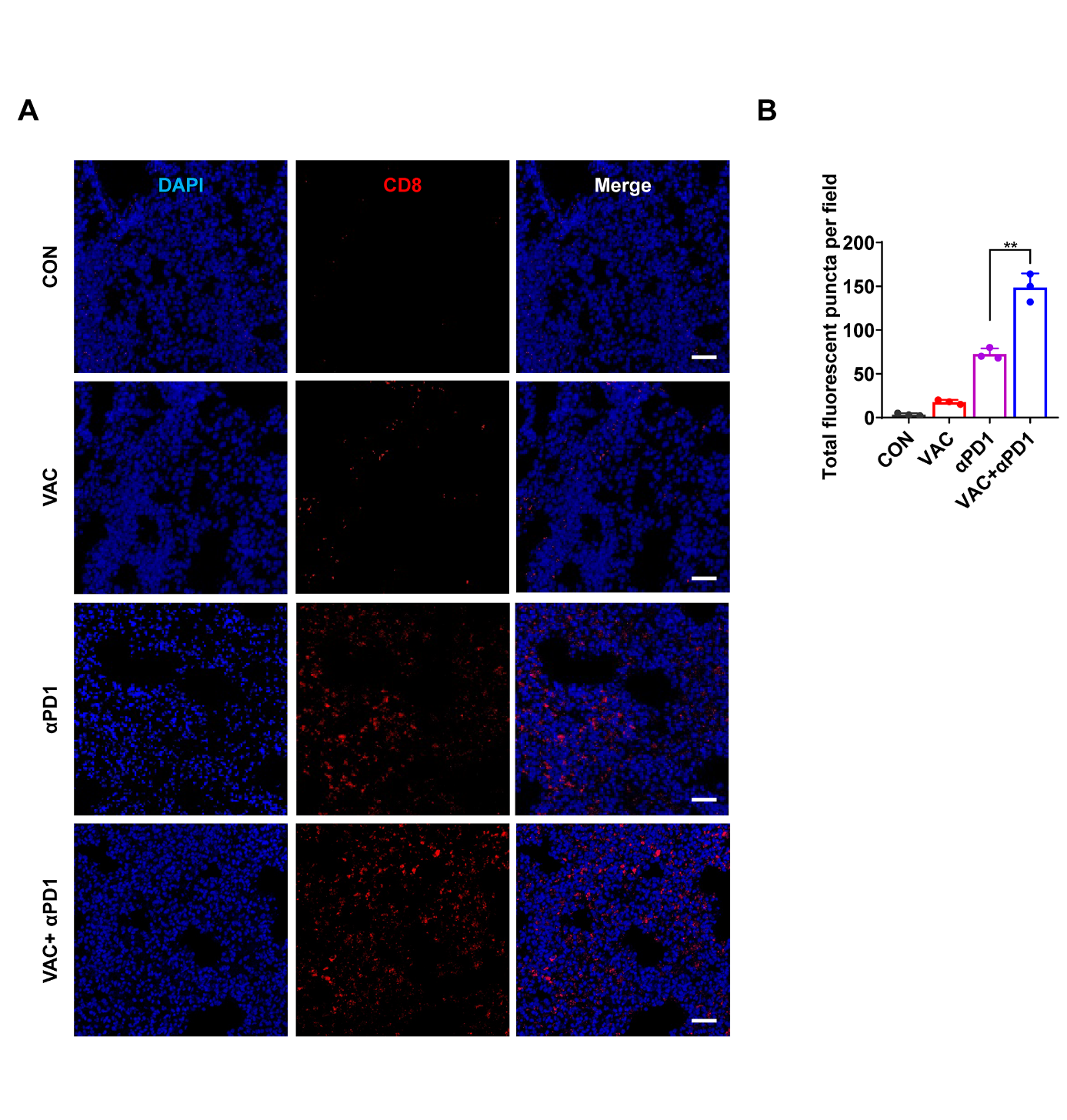


**Figure S3: Immunofluorescence Analysis of CD8^+^ T cell Infiltration in Lung Tissues in Pneumonia Investigation Experiment.** (A) Representative immunofluorescence images of CD8^+^ T cell infiltration in lung tissues of 4T1 tumor-bearing mice treated with PBS (control), tumor vaccine (VAC), αPD1, or VAC+αPD1. Scale bar, 50 μm. (B) Quantification of CD8^+^ T cell numbers in lung tissues across treatment groups (n = 3). Data are mean ± SD, ***p*<0.01.


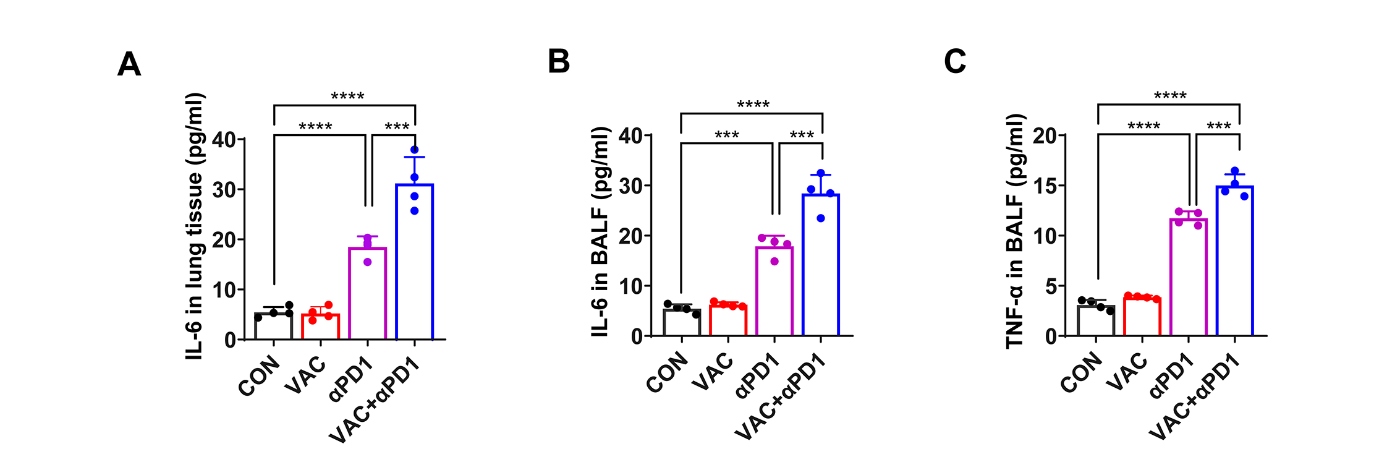


**Figure S4:** **Inflammatory Factors in the Lungs of Mice.**  (A) Lung tissue fluid IL-6 concentrations measured by ELISA (n = 4). (B-C) Concentrations of (B) IL-6 and (C) TNF-α in BALF measured by ELISA (n = 4). Data are presented as mean ± SD, ****p* < 0.001, *****p* < 0.0001.


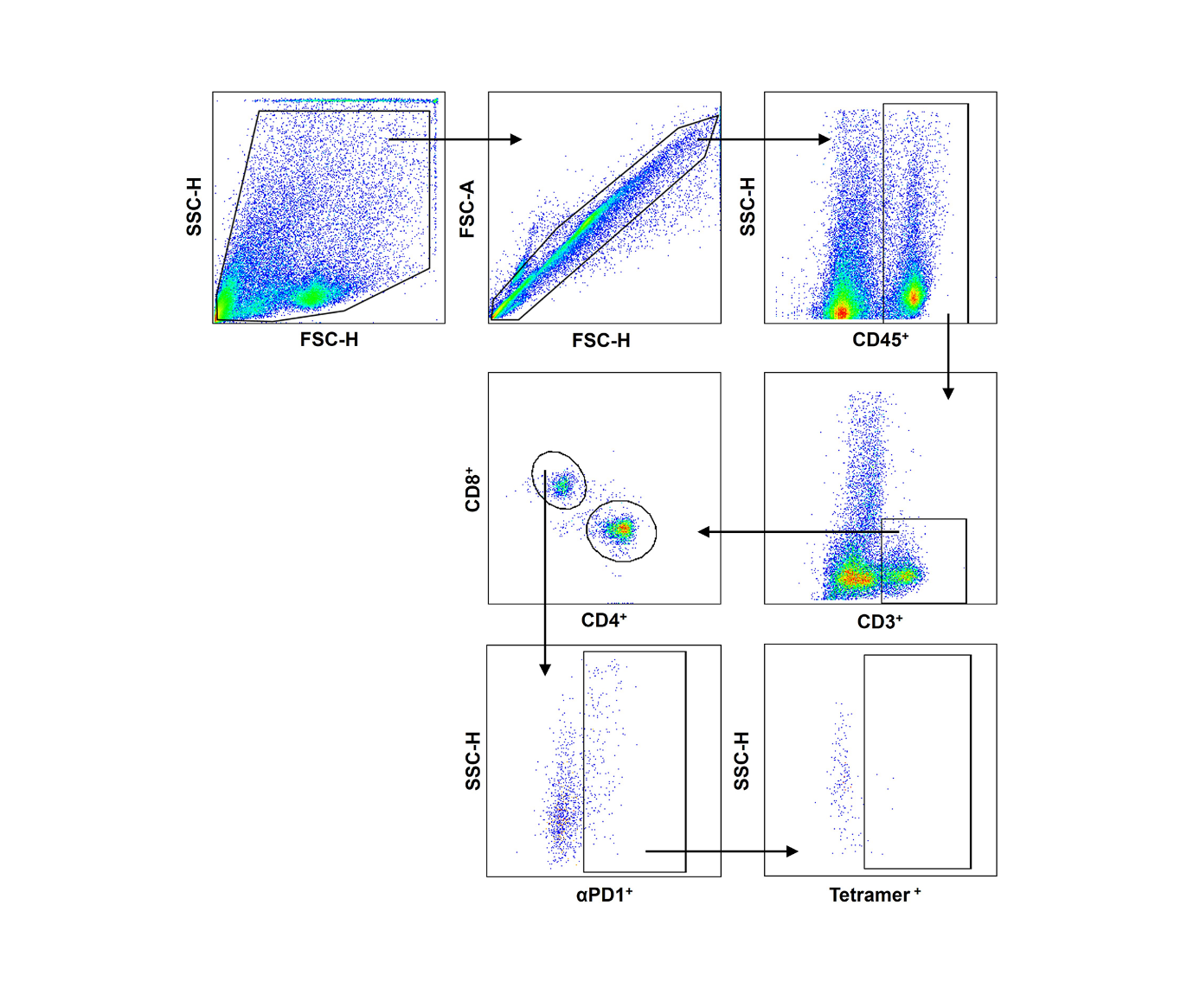
**Figure S5: Flow Cytometry Gating Strategy for Assessing Immune Cell Changes Following CD8 Blockade.**


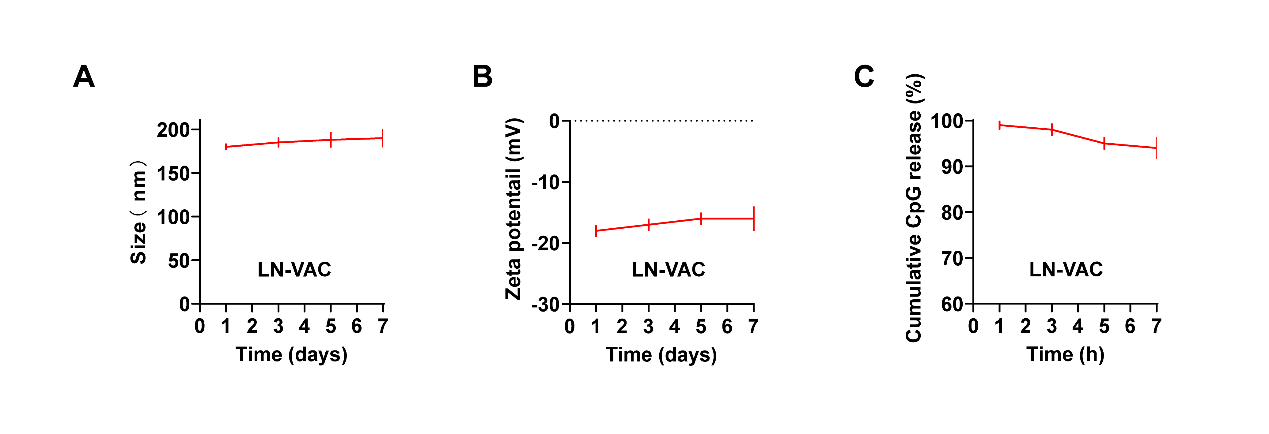


**Figure S6：Characterization of Drug Stability.** (A) Particle size, (B) zeta potential, and (C) CpG release profile of LN-VAC (n = 3). Data are presented as mean ± SD.


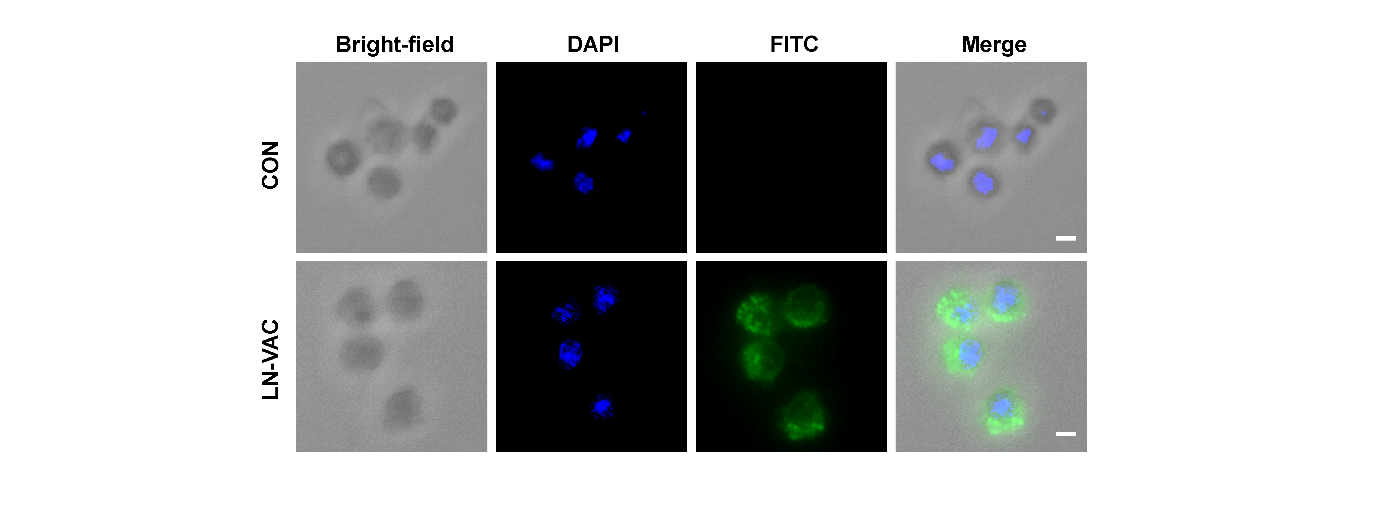


**Figure S7: Immunofluorescence of FITC-labeled LN-VAC uptake by BMDCs after 24 hours co-incubation.** Blue, nuclei (DAPI); green, FITC-LN-VAC. Scale bar, 10 μm.


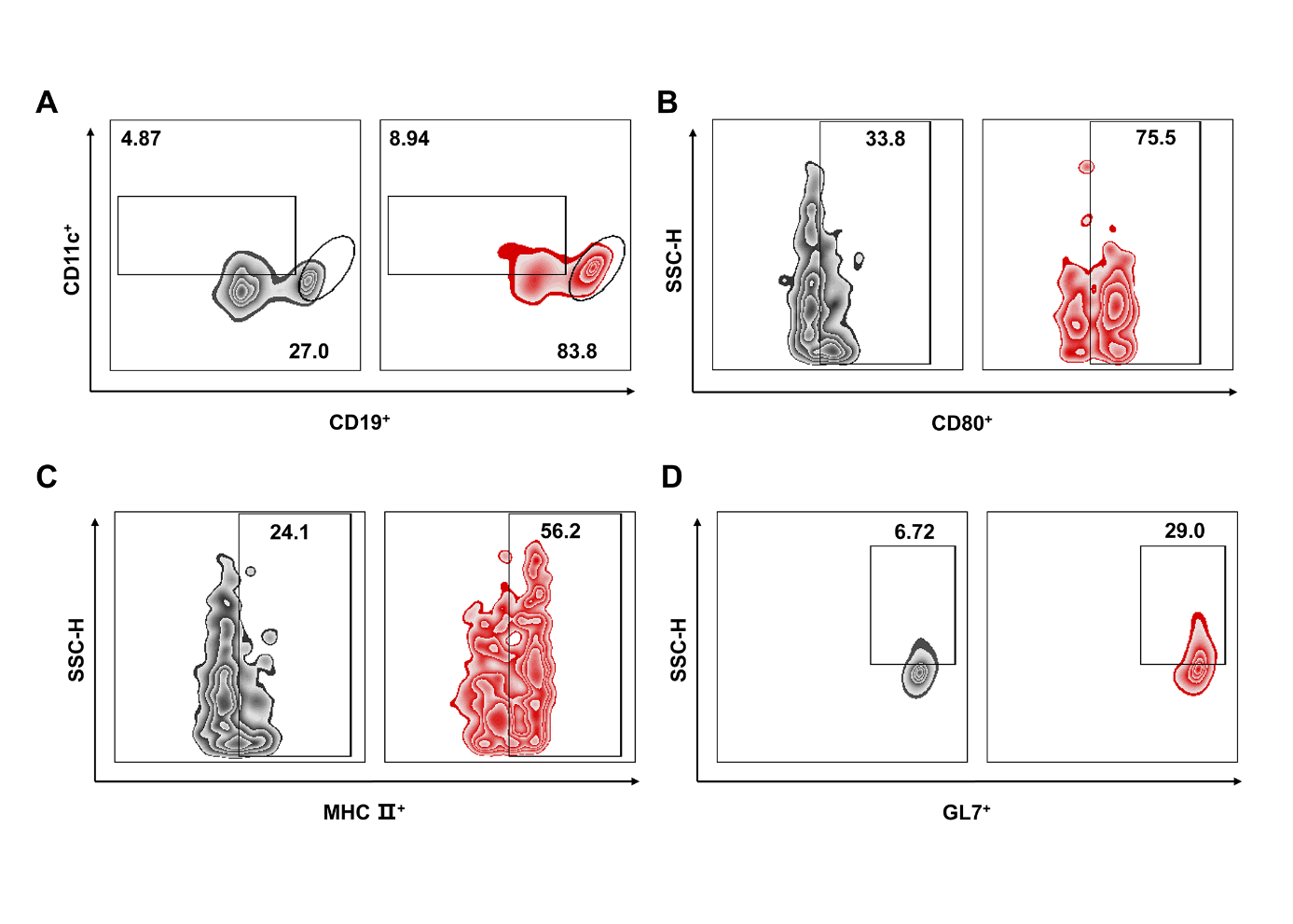
**Figure S8: Representative Flow Cytometry Plots of Lymph Node Immune Activation.**(A)CD11c^+^ and CD19^+^in CD45^+^. (B)CD80^+^ in CD11c^+^. (C) MHC Ⅱ^+^ in CD11c^+^. (D) CD19^+^ GL7^+^ in cd45^+^.


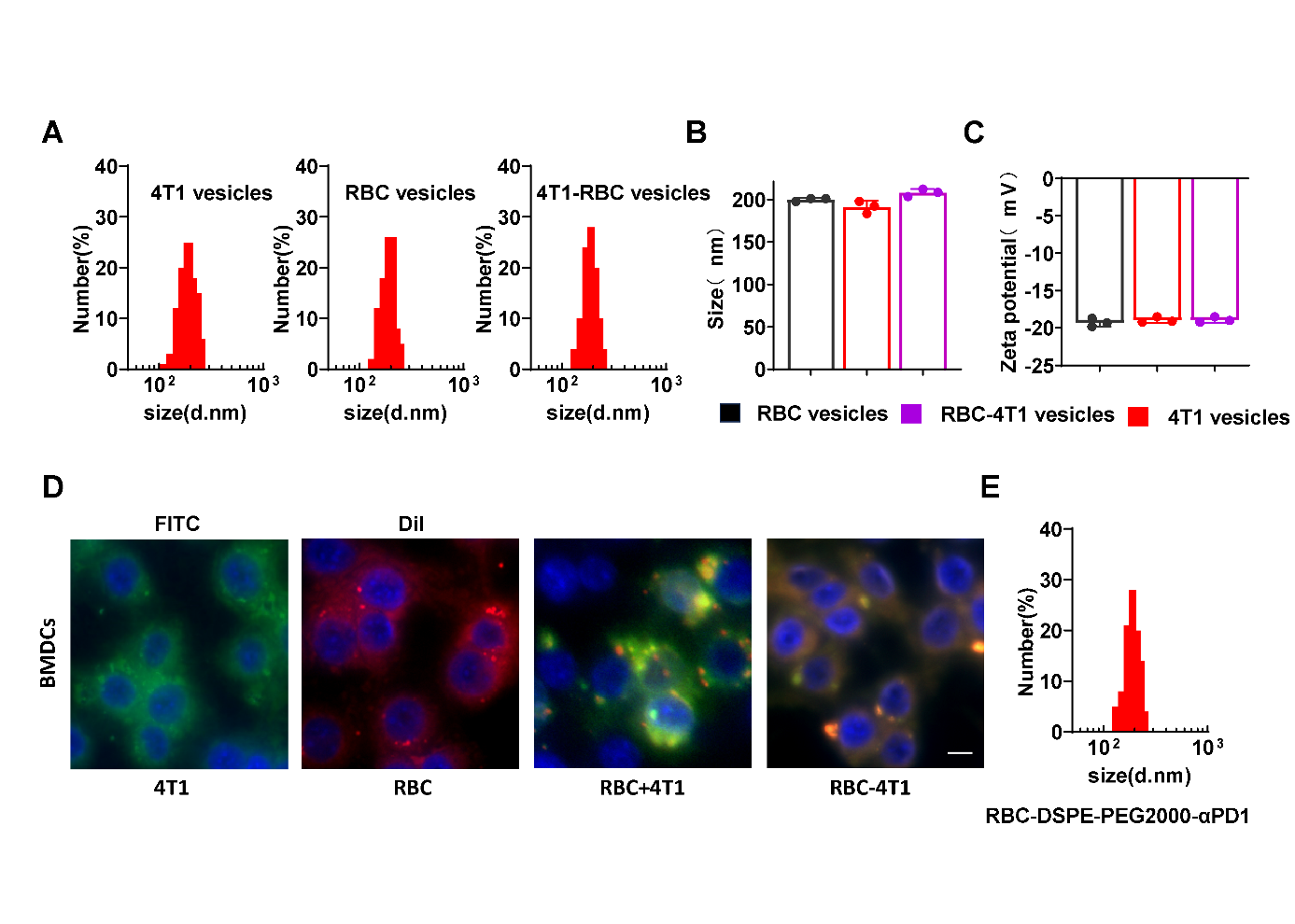
**Figure S9: Characterization of Spleen-Targeted Vaccine.** (A) TEM measurement of Spl-VAC particle size. (B) DLS analysis of Spl-VAC particle sizes. (C) Zeta potential measurements of Spl-VAC (n = 3). (D) IF analysis of membrane fusion and uptake by BMDCs after 6 hours of co-incubation with RBC membrane vesicles, tumor cell membrane vesicles, their mixture, or fused 4T1-RBC membrane vesicles. FITC labels 4T1 cell membranes (green), Dil labels RBC membranes (red), and DAPI labels nuclei (blue). Scale bar, 10 μm. (E) TEM measurement of Spl-αPD1 particle sizes. Data are presented as mean ± SD.


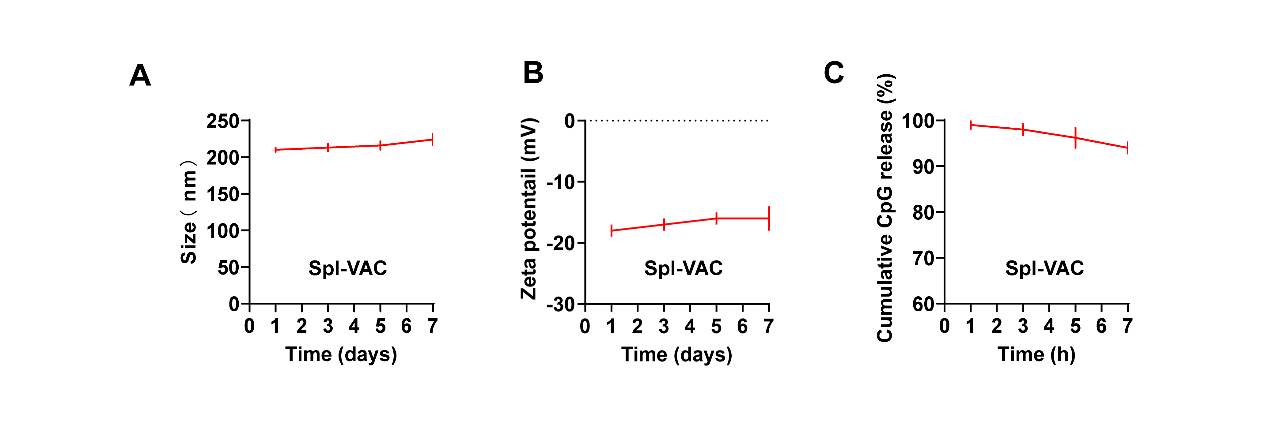


**Figure S10：Characterization of Drug Stability.** (A) Particle size, (B) zeta potential, and (C) CpG release profile of Spl-VAC(n = 3). Data are presented as mean ± SD.


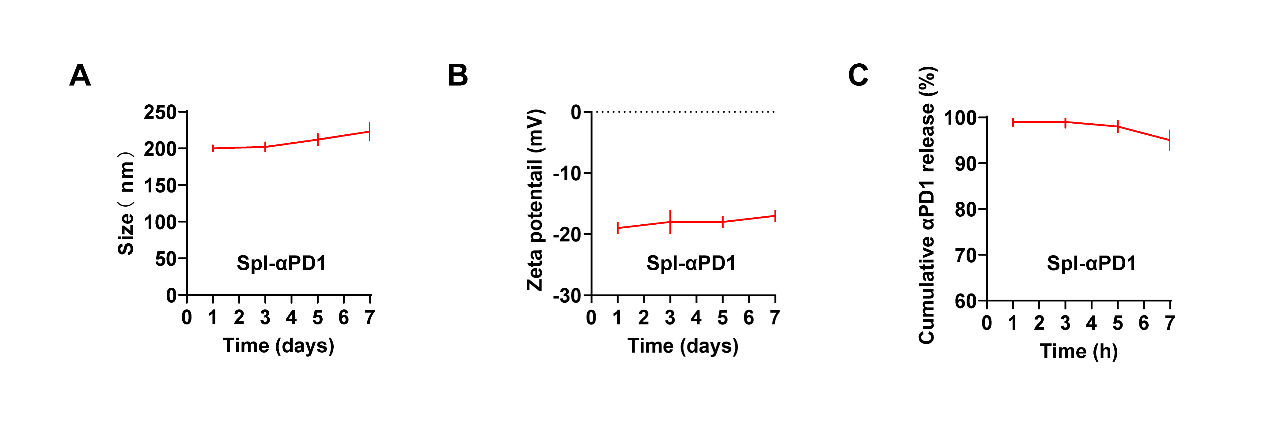


**Figure S11：Characterization of Drug Stability.** (A) Particle size, (B) zeta potential, and (C) CpG release profile of Spl-αPD1(n = 3). Data are presented as mean ± SD.


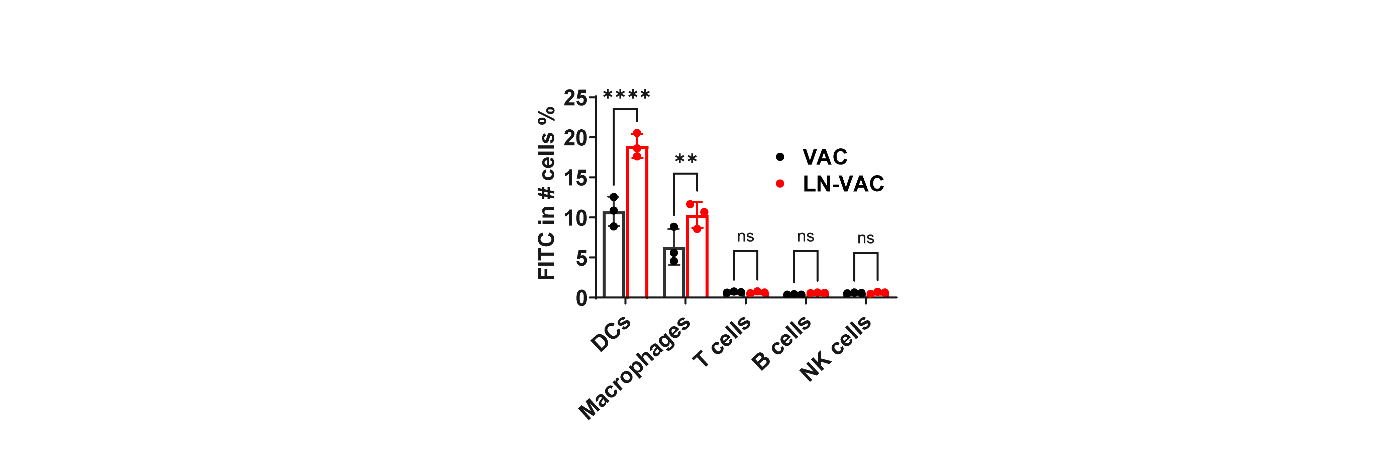


**Figure S12: Flow cytometry analysis of the uptake of lymph node-targeted vaccine in lymph nodes (n = 3).** Data are presented as mean ± SD, *****p* < 0.0001, ns, not significant.


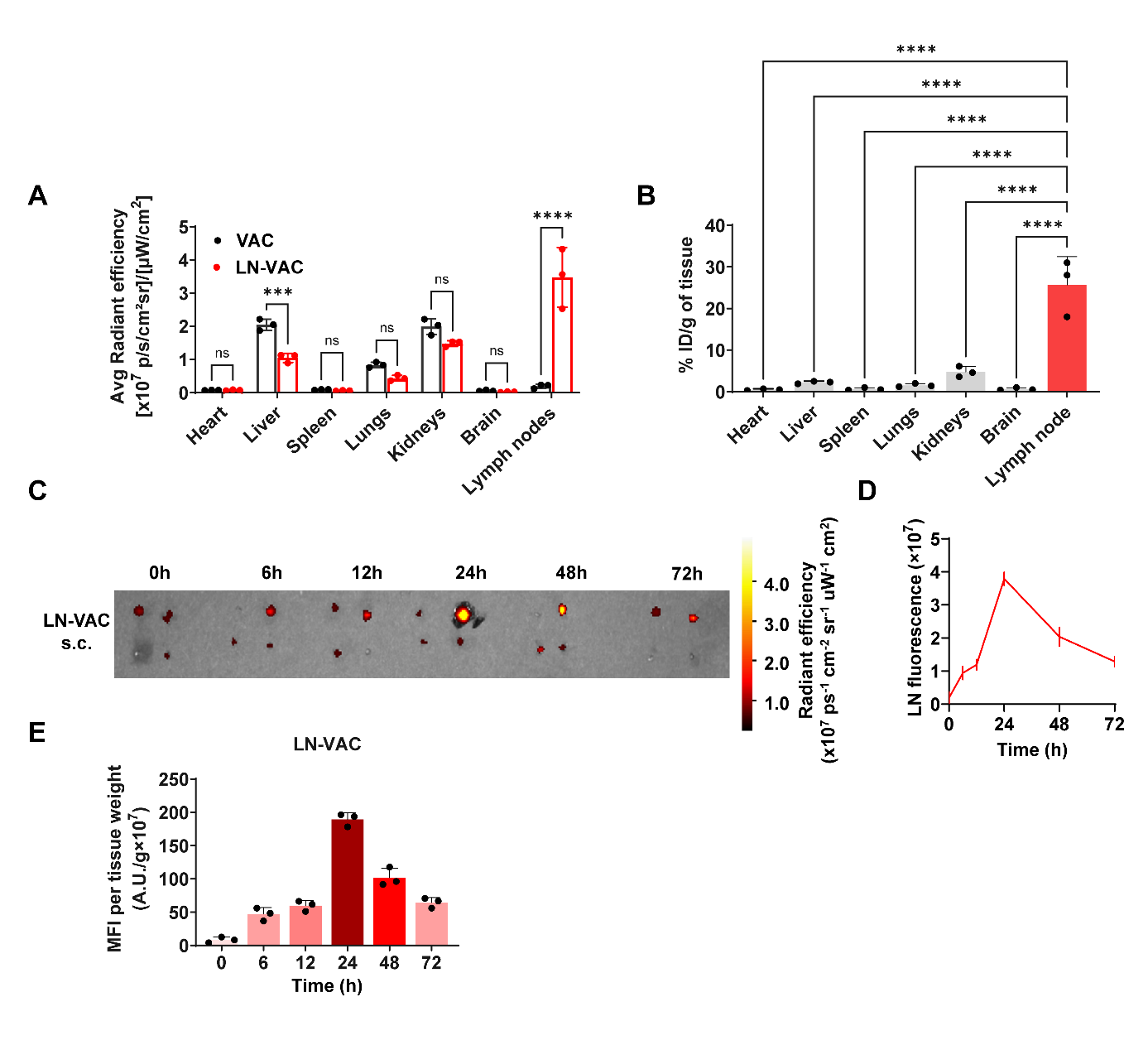


**Figure S13:** **Metabolism of Targeted Tumor Vaccine in Lymph Nodes.** (A) Quantification of *in vivo* fluorescence imaging distribution of FITC-labeled LN-VAC at 24 h after subcutaneous injection (n = 3). (B) *Ex vivo* biodistribution of LN-VAC in draining lymph nodes at the indicated time points after subcutaneous injection, expressed as percentage of injected dose per gram of tissue (%ID/g) (n = 3). (C) IVIS detection of fluorescence intensity in lymph nodes at 0, 6, 12, 24, 48, and 72 hours post-subcutaneous injection of FITC-labeled LN-VAC. (D) Fluorescence intensity in lymph nodes of mice injected with LN-VAC at different time points (n=3). (E) *Ex vivo* fluorescence quantification of draining lymph nodes at indicated time points after subcutaneous injection of LN-VAC. Fluorescence intensity was normalized to tissue weight and expressed as a.u./g (n=3). Data are presented as mean ± SD，****p* < 0.001,*****p* < 0.0001, ns, not significant.


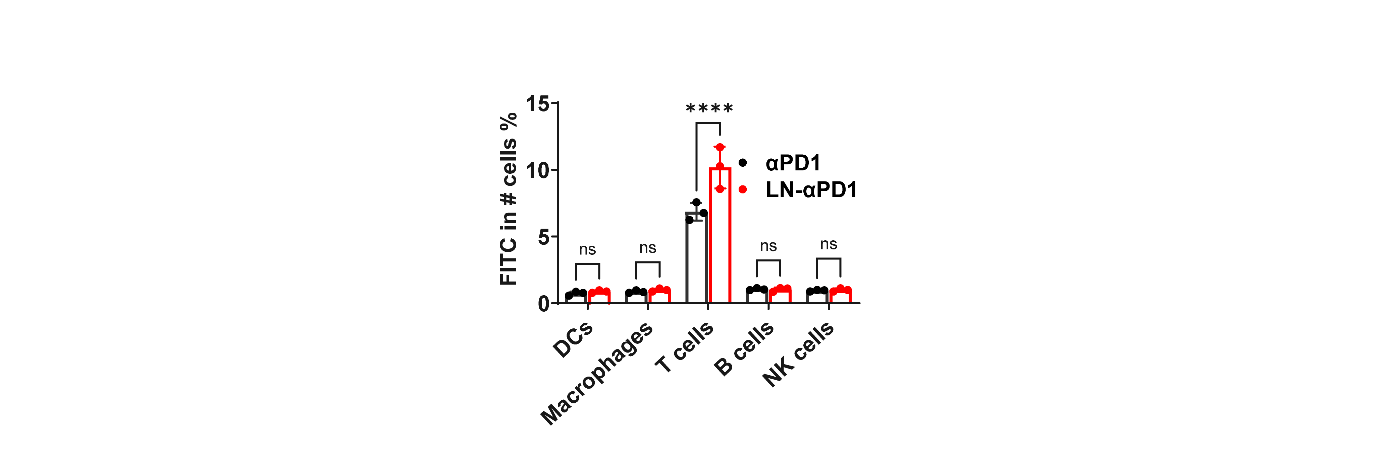


**Figure S14: Flow Cytometry Assessment of LN-αPD1 Uptake in Lymph Nodes (n = 3).** Data are presented as mean ± SD, *****p* < 0.0001, ns, not significant.


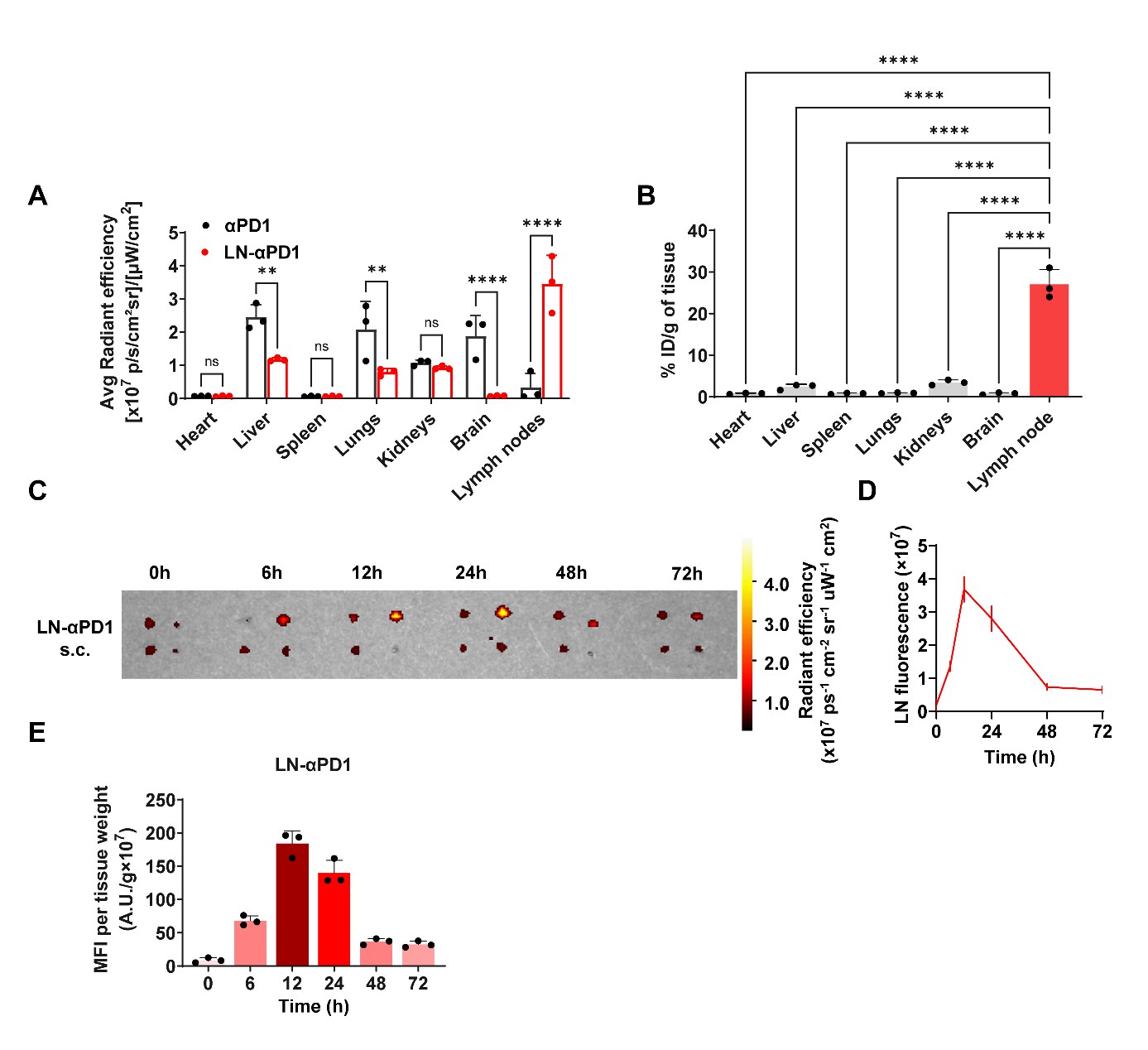
**Figure S15.Metabolism of Targeted αPD1 in Lymph Nodes.** (A) Quantitative analysis of the *in vivo* fluorescence distribution of FITC-labeled LN-αPD1 at 24 h after subcutaneous administration (n = 3). (B) *Ex vivo* biodistribution of LN-αPD1 in draining lymph nodes at the specified time points following subcutaneous injection, reported as %ID/g tissue (n = 3). (C) IVIS imaging of lymph node fluorescence at 0, 6, 12, 24, 48, and 72 h after subcutaneous injection of FITC-labeled LN-VAC. (D) Time-dependent fluorescence intensity in lymph nodes from mice receiving LN-αPD1 (n = 3). (E) *Ex vivo* quantification of fluorescence in draining lymph nodes collected at the indicated time points after subcutaneous injection of LN-αPD1; signals were normalized to tissue weight and expressed as a.u./g×10^7^ (n = 3). Data are presented as mean ± SD，***p* < 0.01,*****p* < 0.0001, ns, not significant.
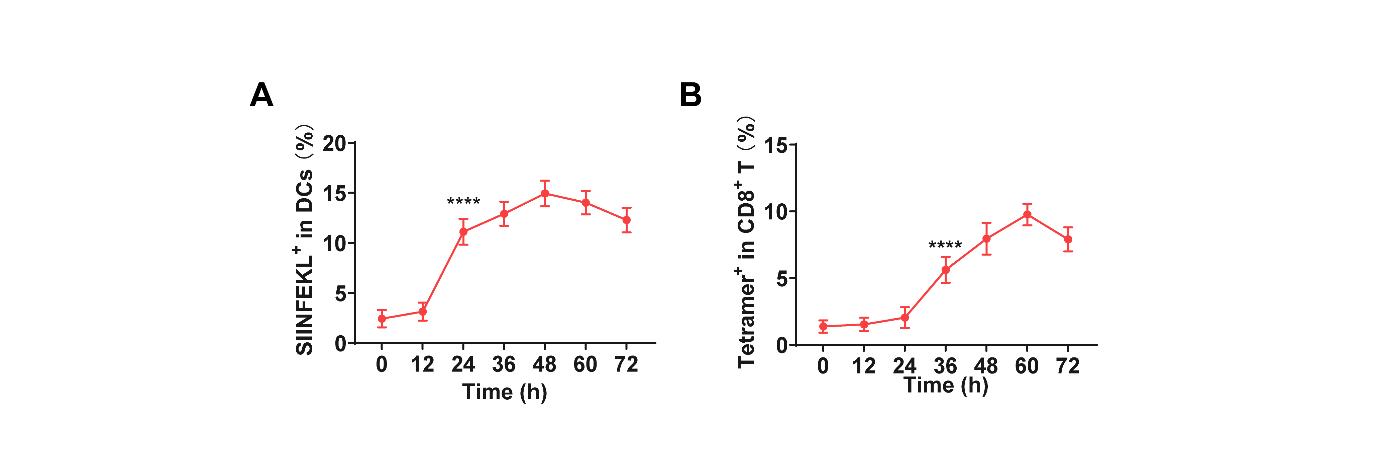


**Figure S16. Antigen Kinetics of LN-VAC.** (A) Flow cytometry analysis of antigen uptake by DCs (n = 3). (B) Flow cytometry analysis of specific receptor expression on T cell surfaces (n = 3). Data are mean ± SD, **** indicates *p* < 0.0001.


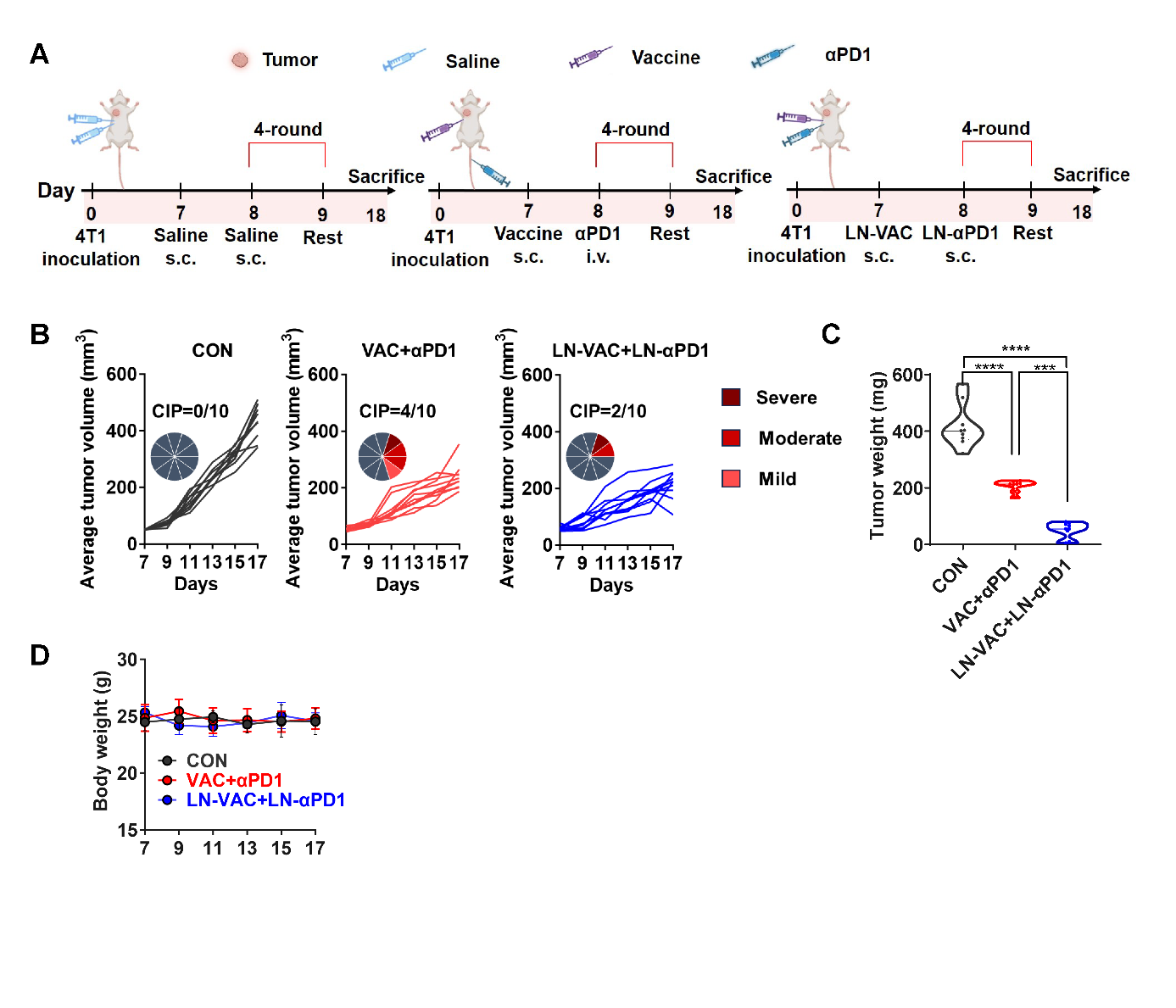


**Figure S17: Antitumor Efficacy of Lymph Node Targeting Delivery Strategy.** (A) Schematic of the treatment schedule for lymph node-targeted delivery in 4T1 tumor-bearing mice.(B)Individual tumor growth curves for 4T1 tumor-bearing mice in CON group, VAC+αPD1 group and LN-VAC+LN-αPD1 group (n = 10)，the red portion of the pie chart represents the number of mice with CIP, with the intensity of the red shade indicating the severity of CIP. (C) Tumor weights at the study endpoint (n = 10). (D) Body weight changes across treatment groups (n = 10). Data are presented as mean ± SD, ****p* < 0.001, *****p* < 0.0001, ns, not significant.


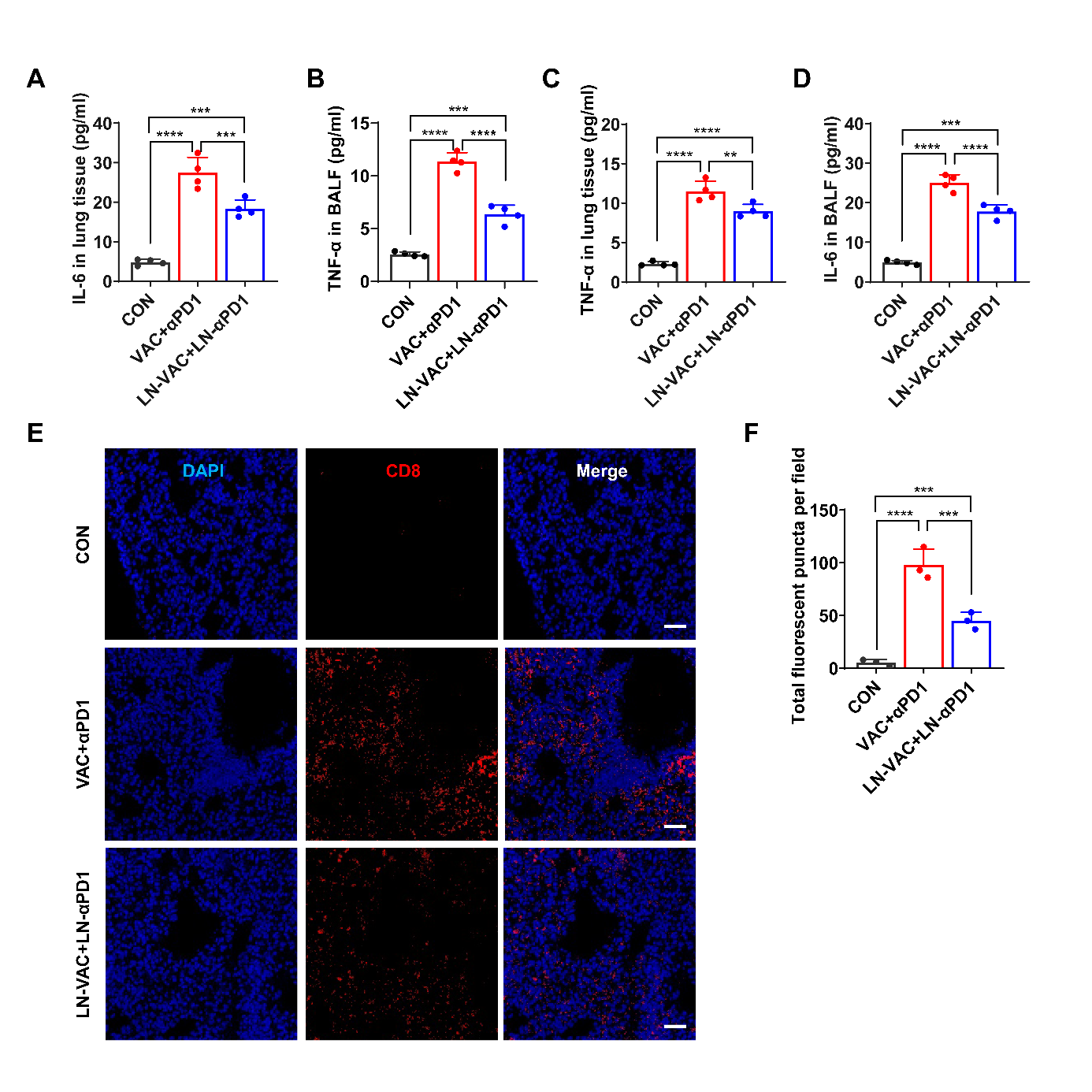
**Figure S18: Characterization of Lung Inflammation in Mice Following Lymph Node-Targeted Therapys.** (A–D) ELISA quantification of (A) IL-6 in lung tissue fluid, (B) IL-6 in BALF, (C) TNF-α in lung tissue fluid, and (D) TNF-α in BALF (n = 4). (E) Representative immunofluorescence images of CD8^+^ T cell infiltration in lung tissues of 4T1 tumor-bearing mice treated with PBS (control), tumor vaccine and αPD1 (VAC+αPD1), LN-VAC+LN-αPD1. Scale bar, 50 μm. (F) Quantification of CD8^+^ T cell numbers in lung tissues across treatment groups (n = 3). Data are presented as mean ± SD, ***p* < 0.01, ****p* < 0.001, *****p* < 0.0001.


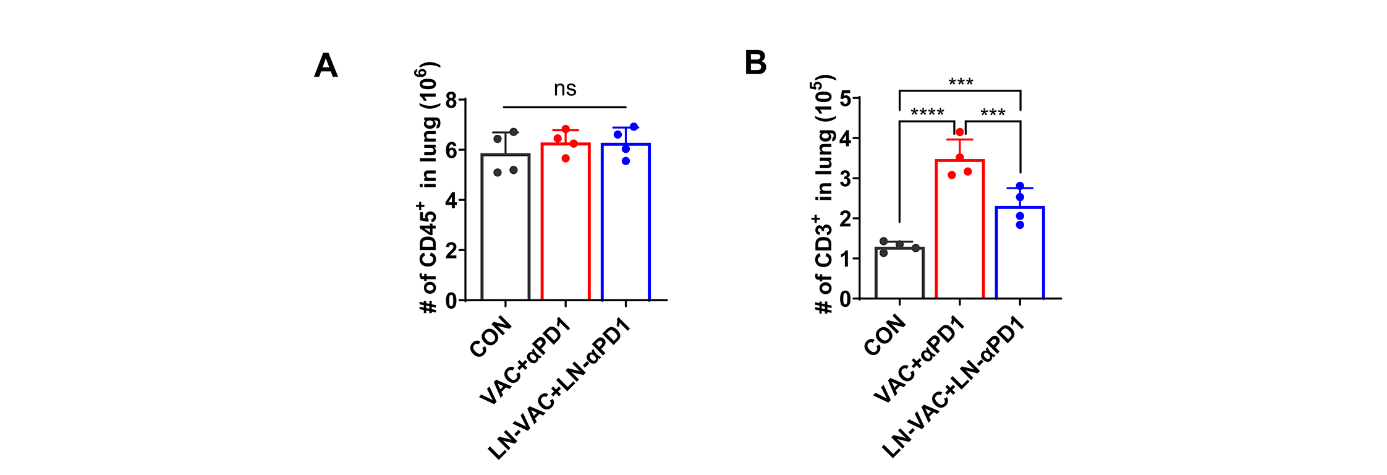
**Figure S19: Flow Cytometric Analysis of Changes in Lung Lymphocytes in Mice Following Lymph Node-Targeted Therapy.** (A-B) Flow cytometry analysis of (A) CD45^+^ cells and (B) CD3^+^ T cells in lung tissues post-treatment (n = 4). Data are mean ± SD, ****p*<0.001, *****p*<0.0001, ns, not significant.


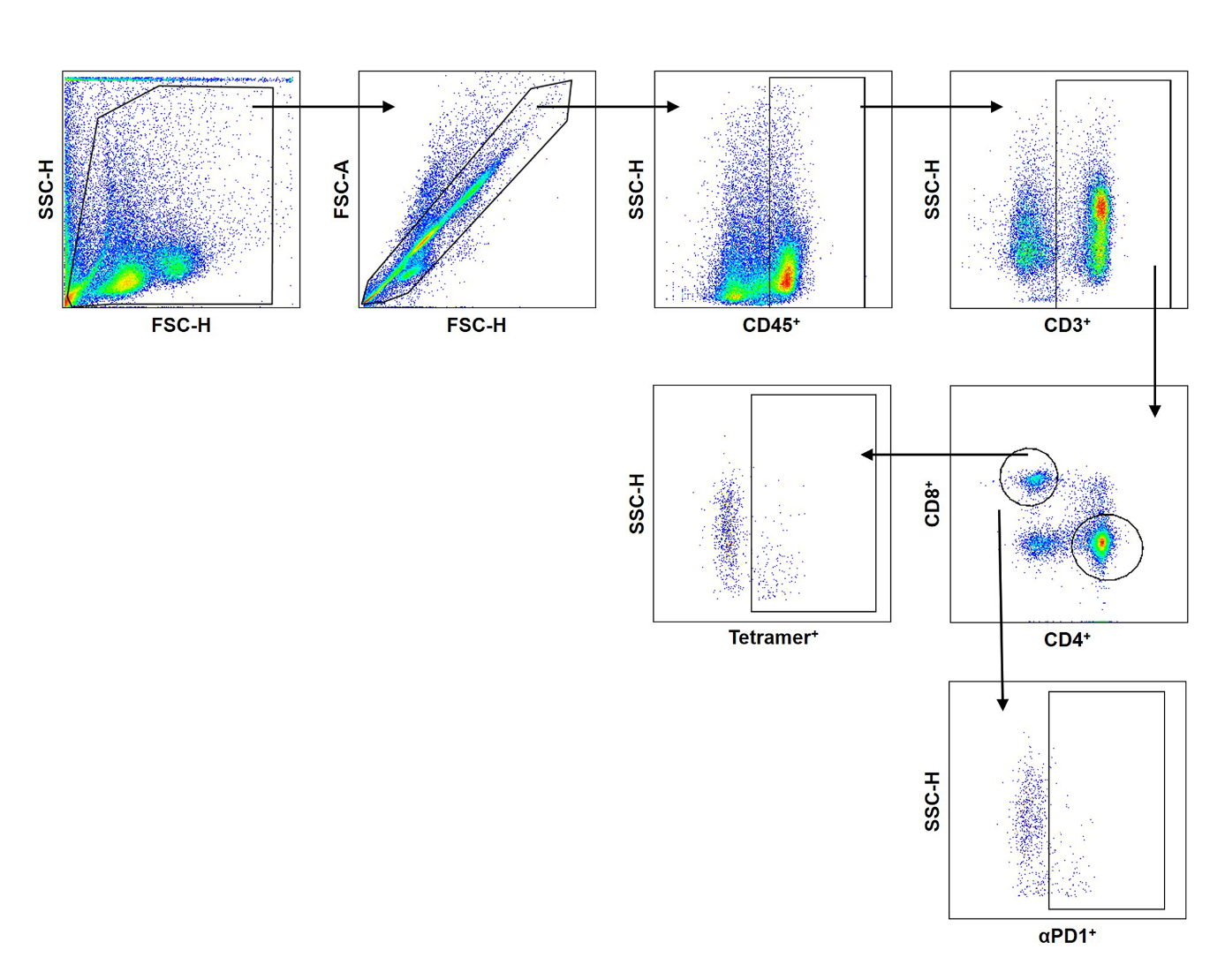


**Figure S20:** **Flow Cytometry Gating Strategy for Lymph Node Targeting Experiment.**


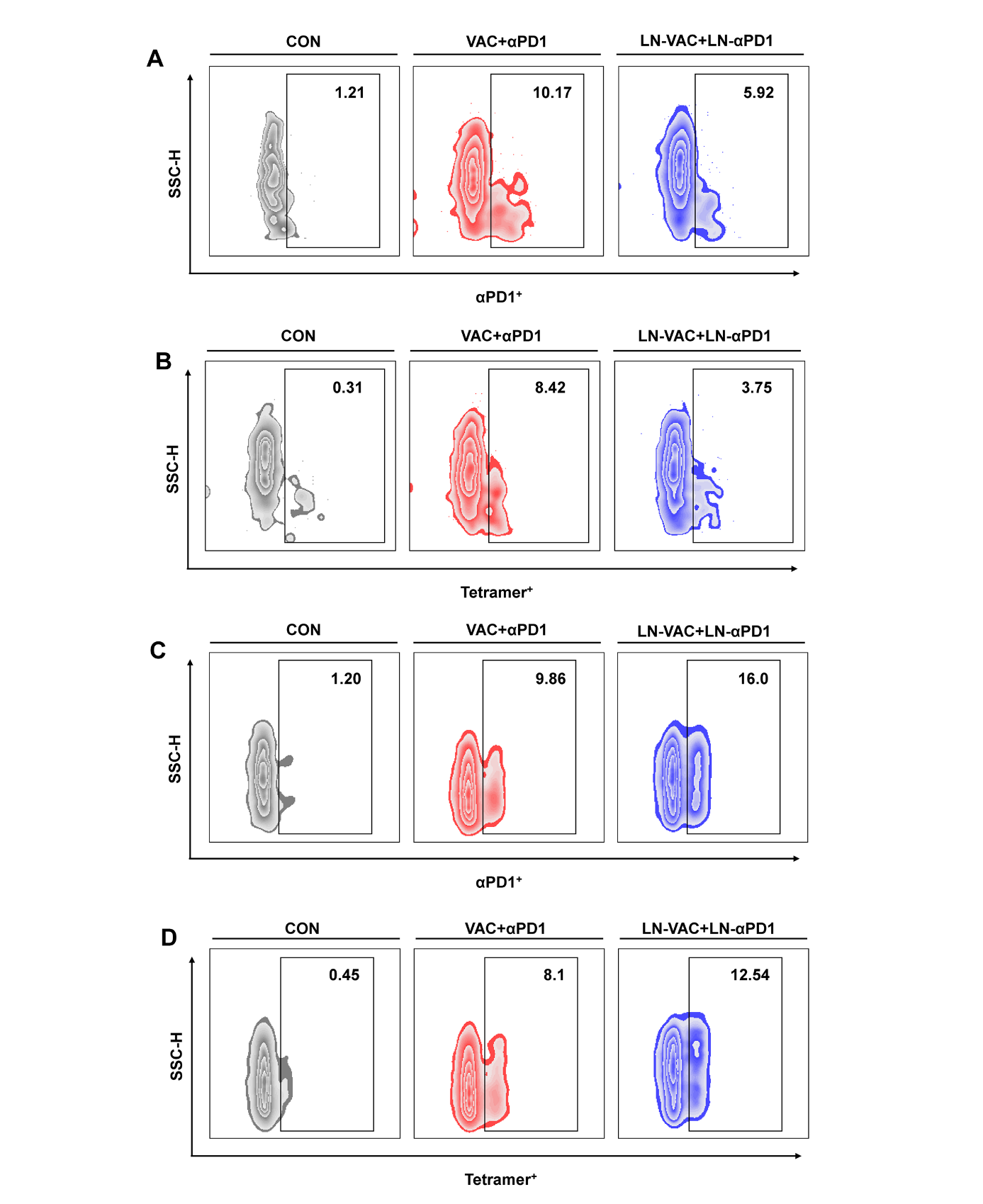
 **Figure S21:** **Representative Flow Cytometry Plots for the Lymph Node Targeting Experiment.** (A) PD1-blocked CD8^+^ T cells in lung tissues. (B) Tetramer^+^ CD8^+^ T cells in lung tissues. (C) PD1-blocked CD8^+^ T cells in lymph nodes. (D) Tetramer^+^ CD8^+^ T cells in lymph nodes.
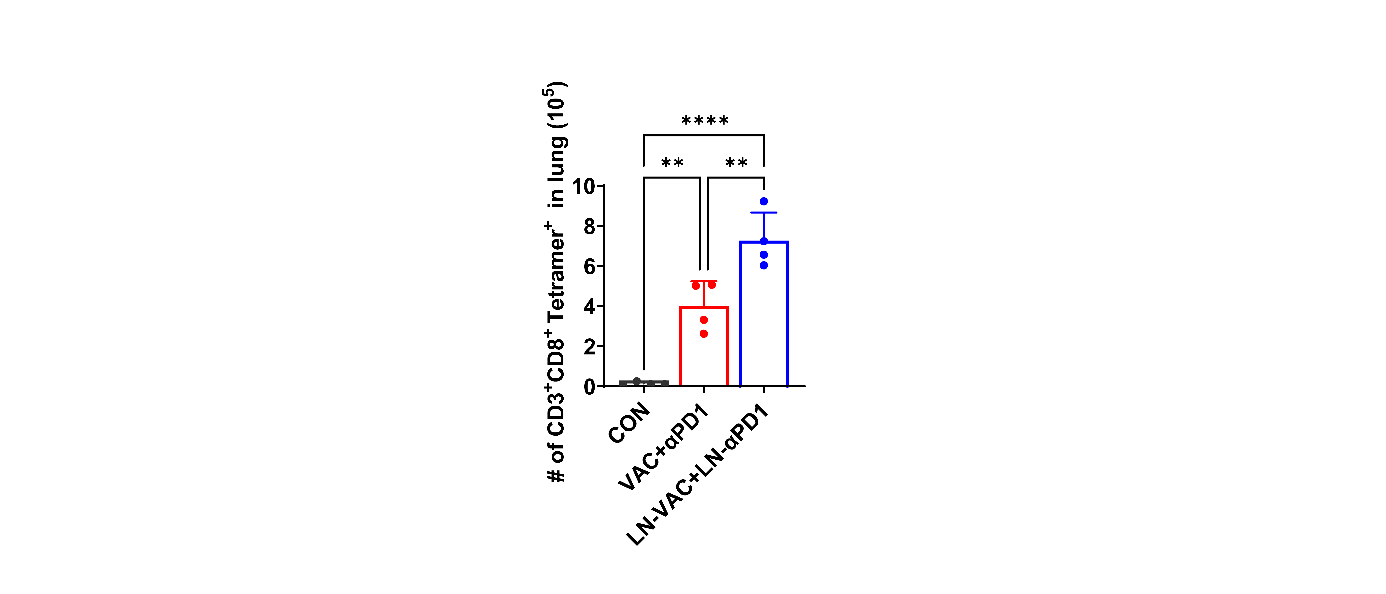
**Figure S22: Flow cytometry quantification of Tumor Specific-T cells in lung tissues (n = 4).** Data are mean ± SD, **p*<0.05, ****p*<0.001, *****p*<0.0001.


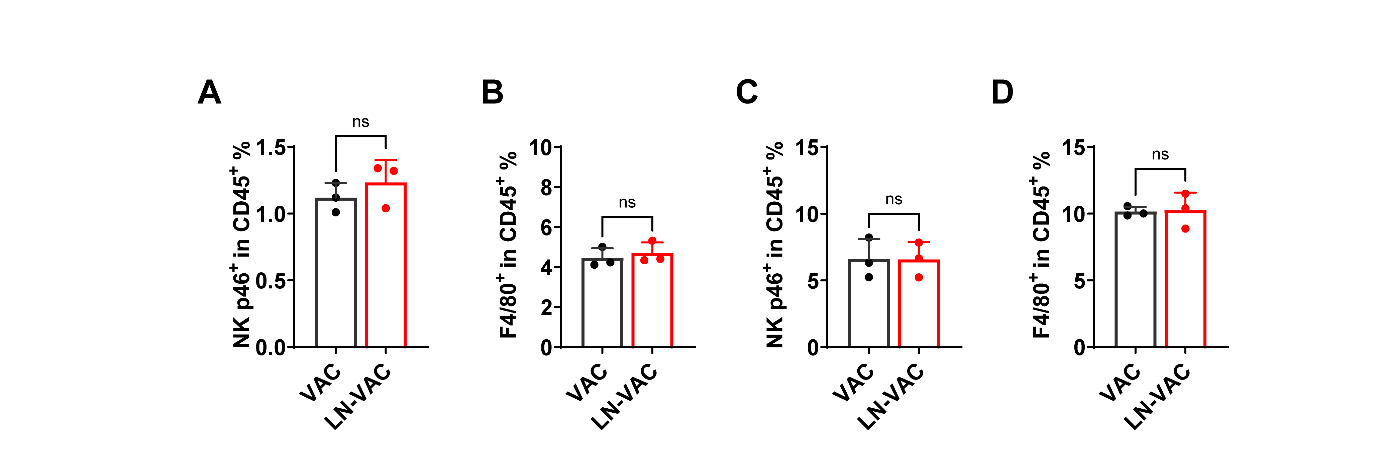
 **Figure S23. Effects of Lymph Node–targeted Tumor Vaccine on Immune Cell populations.** (A) Proportion of NK cells among CD45⁺ cells in lymph nodes (n = 3). (B) Proportion of macrophages among CD45⁺ cells in lymph nodes (n = 3). (C) Proportion of NK cells among CD45⁺ cells in peripheral blood (n = 3). (D) Proportion of macrophages among CD45⁺ cells in peripheral blood (n = 3). Data are presented as mean ± SD, ns, not significant.


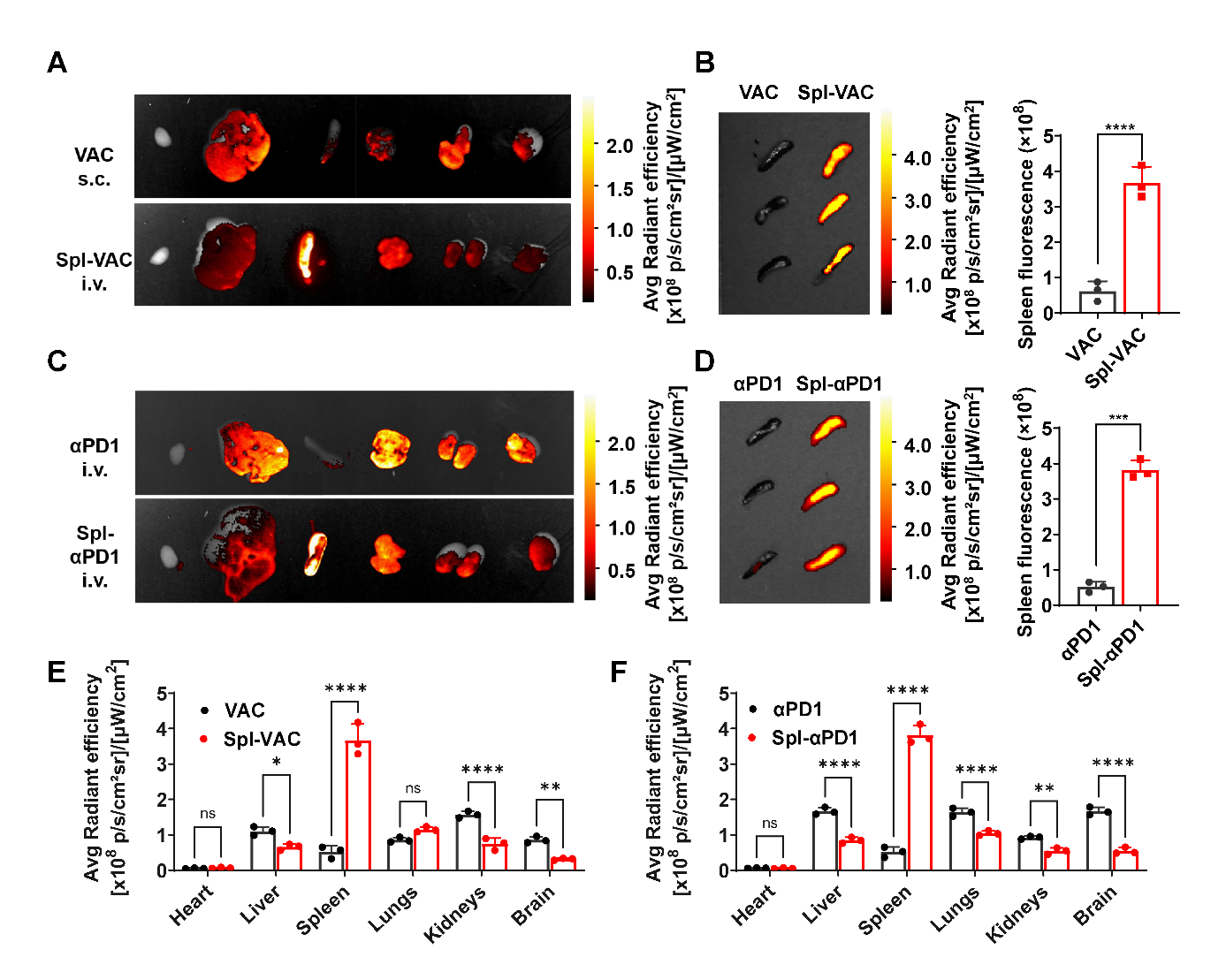


**Figure S24. *In Vivo* Distribution Analysis of Tumor Vaccine and αPD1 Using IVIS.** (A) *In vivo* fluorescence imaging of FITC-labeled Spl-VAC distribution 2 hours post-intravenous injection (organs from left to right: heart, liver, spleen, lungs, kidneys, brain) (n = 3). (B) Spleen imaging and quantitative fluorescence analysis of Spl-VAC accumulation (n = 3). (C) *In vivo* fluorescence imaging of FITC-labeled Spl-αPD1 distribution 2 hours post-intravenous injection (organs from left to right: heart, liver, spleen, lungs, kidneys, brain) (n = 3). (D) Spleen imaging and quantitative fluorescence analysis of Spl-αPD1 accumulation (n = 3). (E-F) Quantitative assessment of the in vivo fluorescence distribution of (E) FITC-labeled Spl-VAC and (F) FITC-labeled Spl-αPD1 at 2 hours after intravenous injection (n = 3). Data are mean ± SD, **p*<0.05, ***p*<0.01, ****p*<0.001, *****p*<0.0001, ns, not significant.


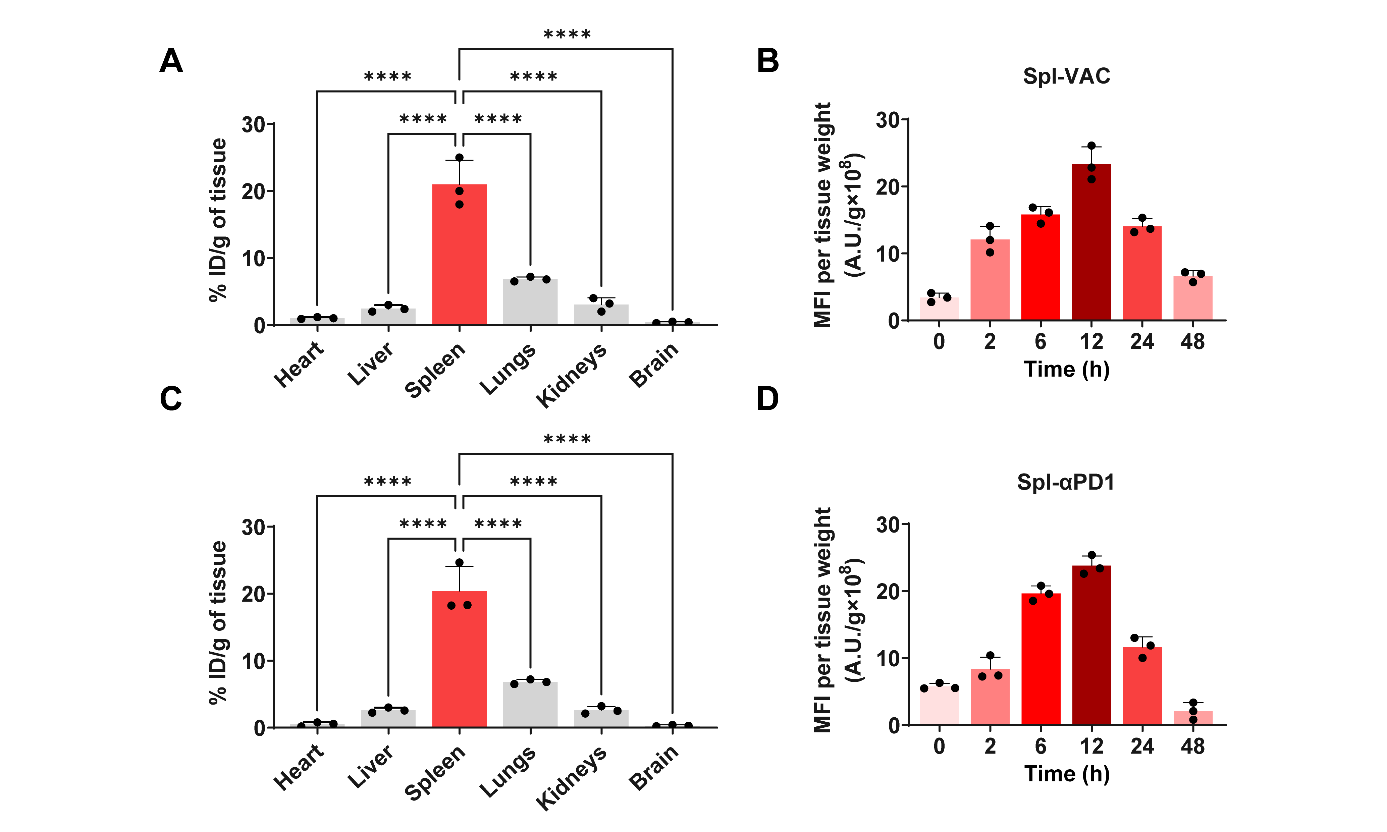


**Figure S25. *Ex vivo* biodistribution and fluorescence quantification of Spl-VAC and Spl-αPD1 in the spleen after intravenous administration.** (A) *Ex vivo* biodistribution of Spl-VAC in spleen at the indicated time points after intravenous injection, expressed as percentage of injected dose per gram of tissue (%ID / g) (n = 3). (B) *Ex vivo* fluorescence quantification of spleen at indicated time points after intravenous injection of Spl-VAC. Fluorescence intensity was normalized to tissue weight and expressed as a.u./g (n = 3). (C) *Ex vivo* biodistribution of Spl-αPD1 in spleen at the indicated time points after intravenous injection of Spl-VAC, expressed as percentage of injected dose per gram of tissue (%ID / g) (n = 3). (D) *Ex vivo* fluorescence quantification of spleen at indicated time points after intravenous injection of Spl-αPD1. Fluorescence intensity was normalized to tissue weight and expressed as a.u./g (n=3). Data are presented as mean ± SD，*****p* < 0.0001, ns, not significant.


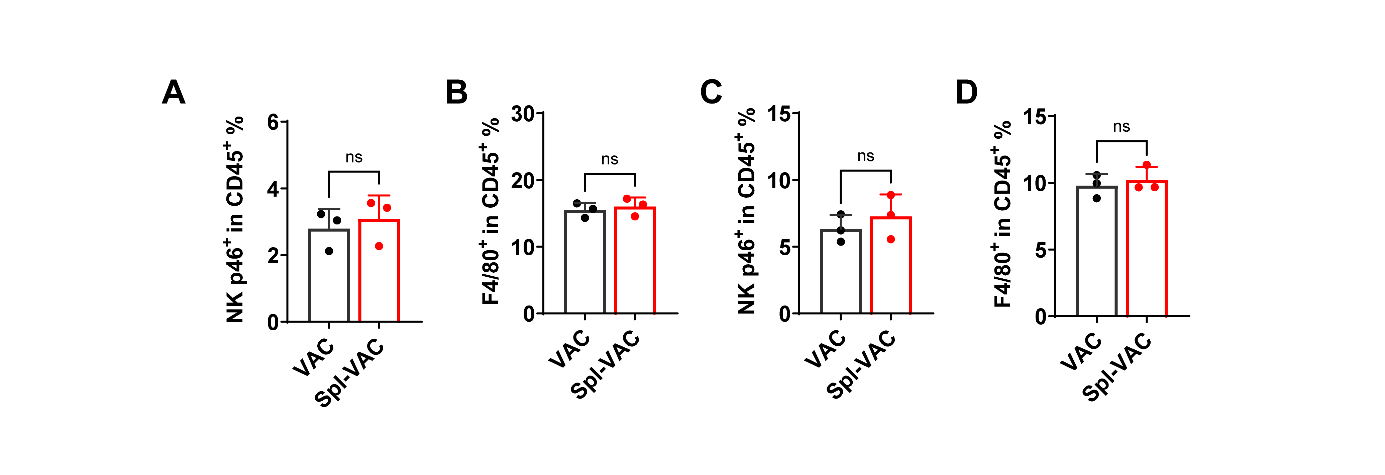
 **Figure S26. Effects of Spleen–targeted Tumor Vaccine on Immune Cell Populations.** (A) Proportion of NK cells among CD45⁺ cells in spleen (n = 3). (B) Proportion of macrophages among CD45⁺ cells in spleen (n = 3). (C) Proportion of NK cells among CD45⁺ cells in peripheral blood (n = 3). (D) Proportion of macrophages among CD45⁺ cells in peripheral blood (n = 3). Data are presented as mean ± SD, ns, not significant.


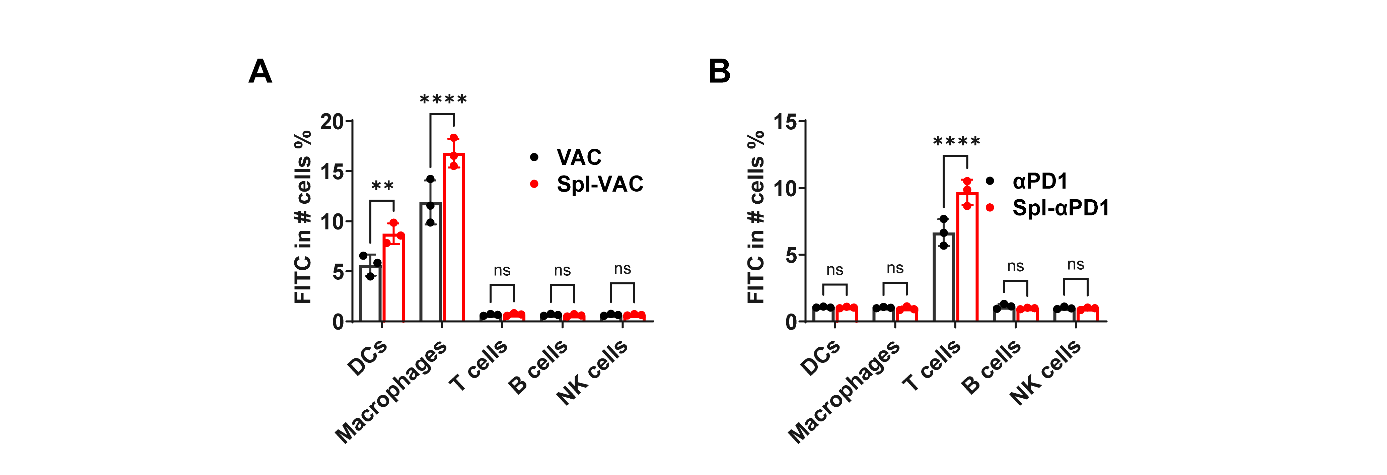
**Figure S27:** **Flow Cytometry Analysis of Lymph Node-targeted Formulation Uptake in the Spleen.** (A) Uptake of spleen-targeted tumor vaccine in the spleen (n = 3). (B) Uptake of spleen-targeted αPD1 in the spleen (n = 3). Data are presented as mean ± SD, ***p* < 0.01, *****p* < 0.0001, ns, not significant.


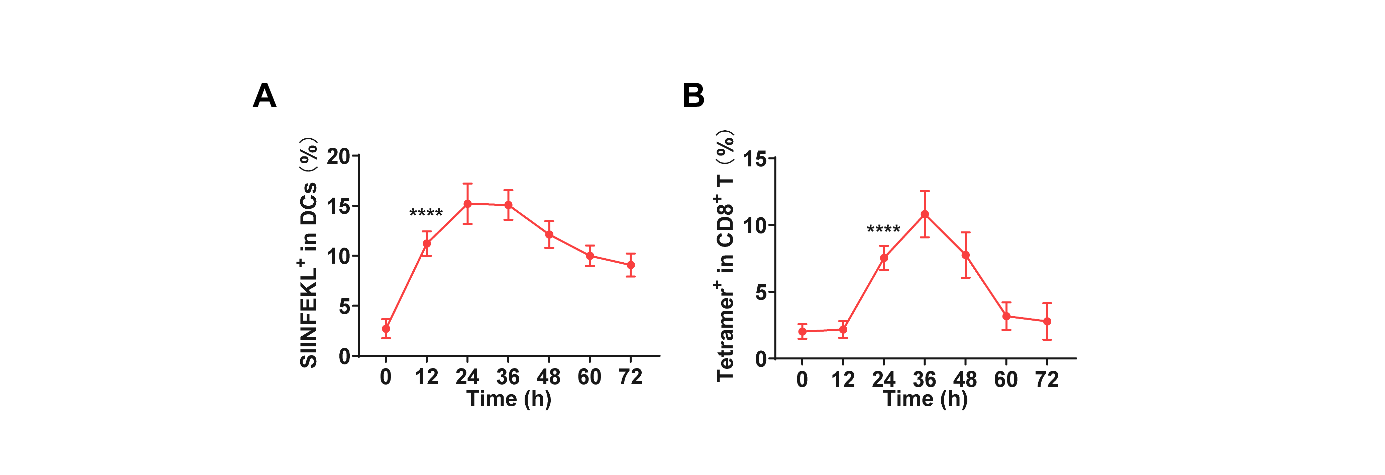
**Figure S28. Antigen Kinetics of Spl-VAC.** (A) Flow cytometry analysis of antigen uptake by DCs (n = 3). (B) Flow cytometry analysis of specific receptor expression on T cell surfaces (n = 3). Data are mean ± SD, *****p* < 0.0001.


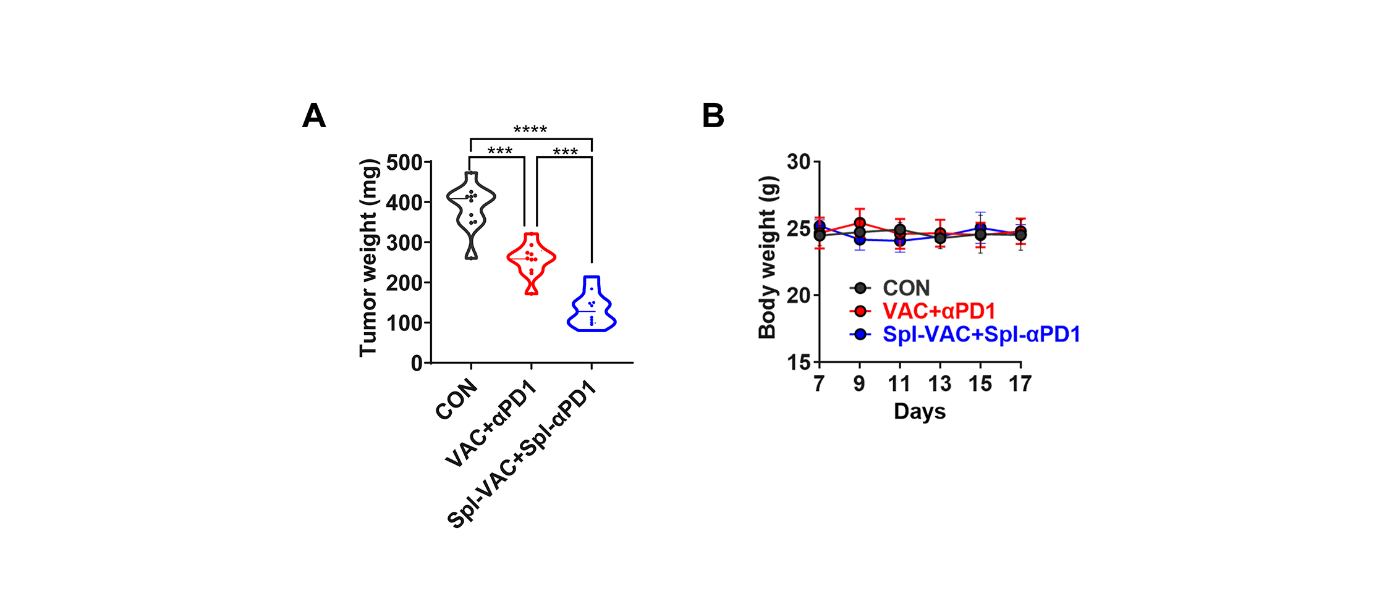
**Figure S29: Changes in Tumor Mass and Body Weight in Mice Following Spleen-Targeted Therapy.** (A) Tumor weight in different groups of mice at the end of treatment (n = 10). (B) Body weight changes across treatment groups (n = 10). Data are presented as mean ± SD, ***p* < 0.01, ****p* < 0.001, *****p* < 0.0001; ns, not significant.


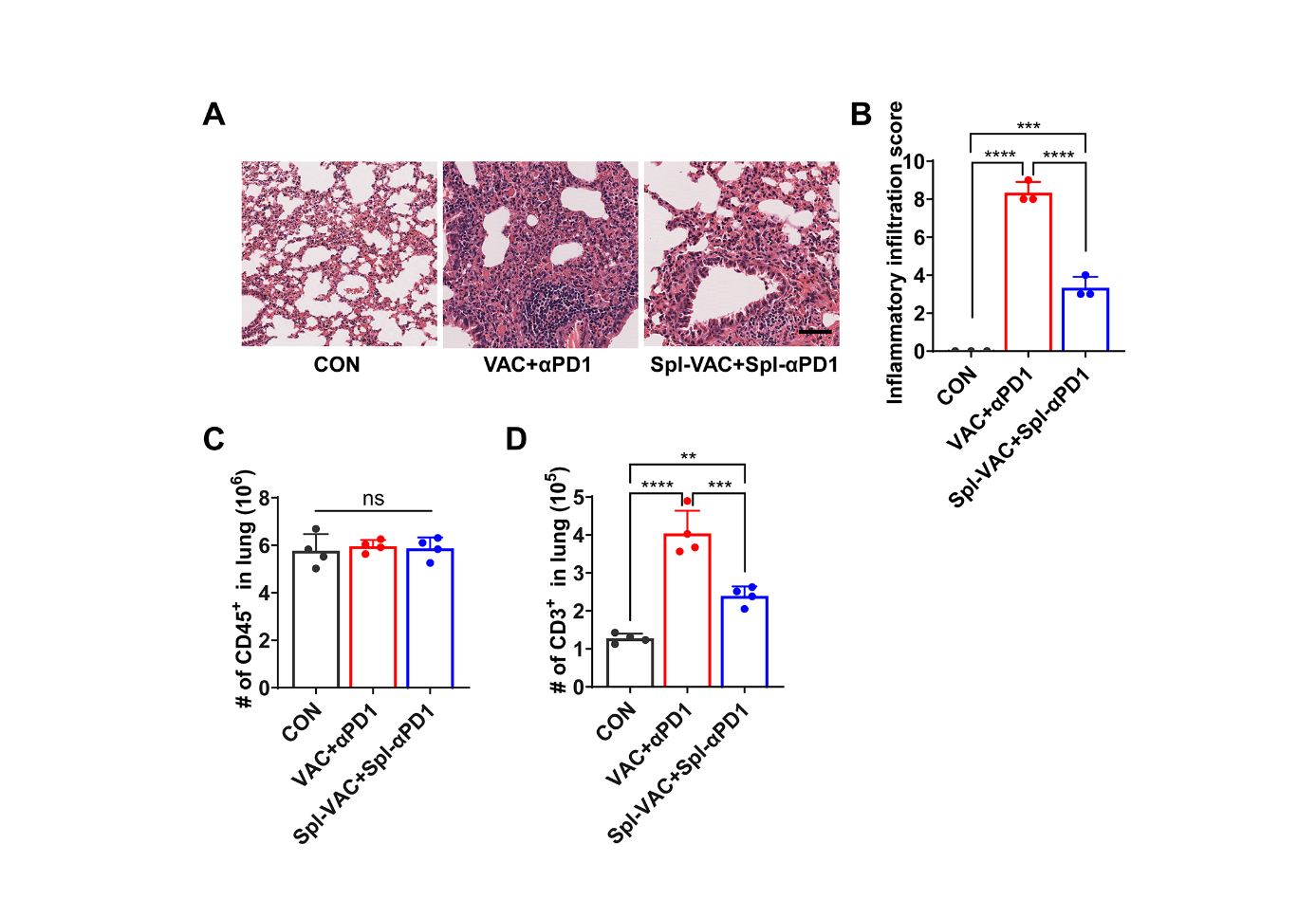
**Figure S30: Inflammatory Changes in the Lungs of Mice with Spleen-Targeted Delivery Strategy.** (A) Assessment of inflammatory infiltration in mouse lung tissue using H&E staining, Scale bar, 50 μm. (B) Quantified inflammatory infiltration scores indicating CIP severity (n = 3). (C-D) Flow cytometry analysis of (C) CD45^+^ cells and (D) CD3^+^ T cells in lung tissues post-treatment (n = 4). Data are presented as mean ± SD, ***p* < 0.01, ****p* < 0.001, *****p* < 0.0001; ns, not significant.


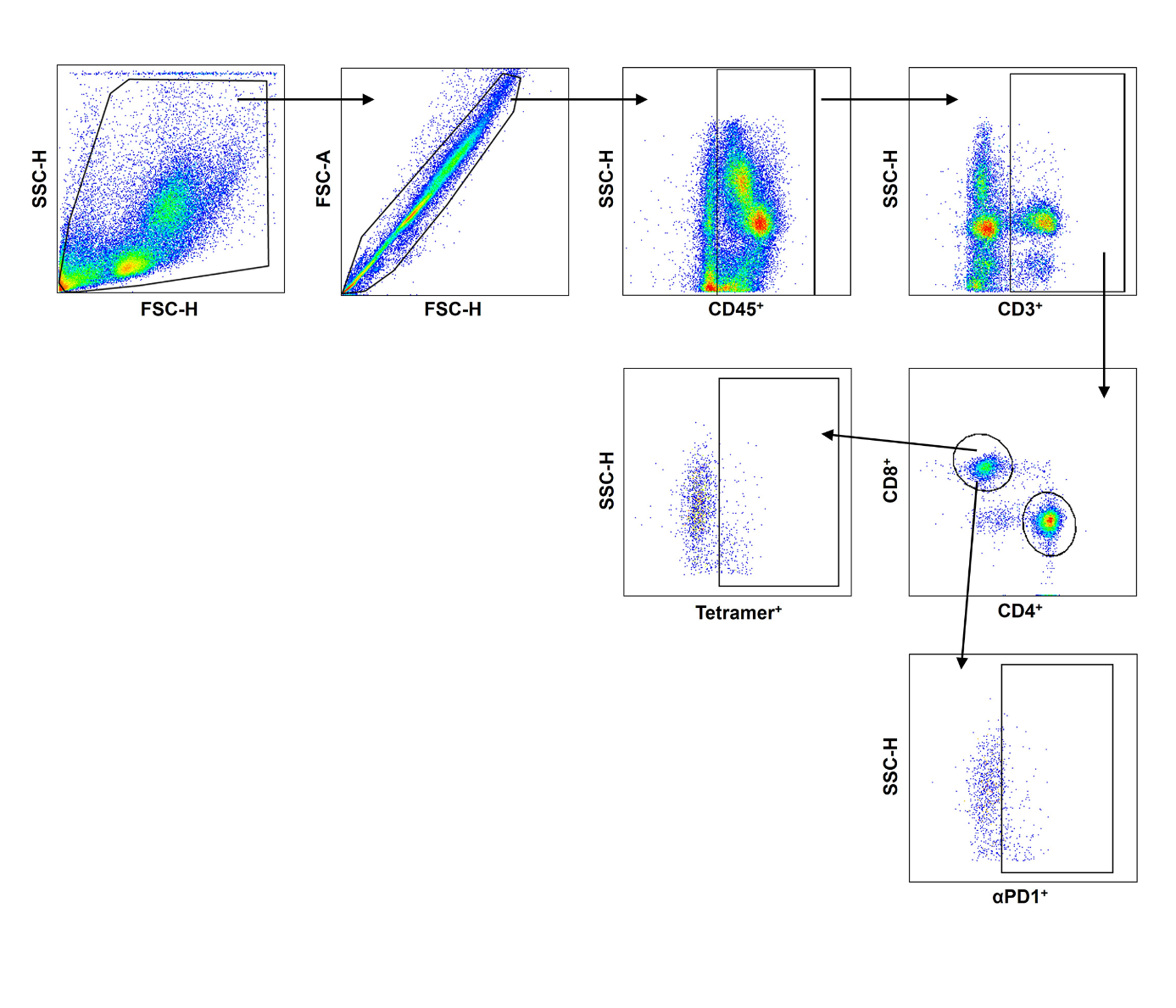
**Figure S31: Flow Cytometry Gating Strategy for Spleen Targeting Experiment.**


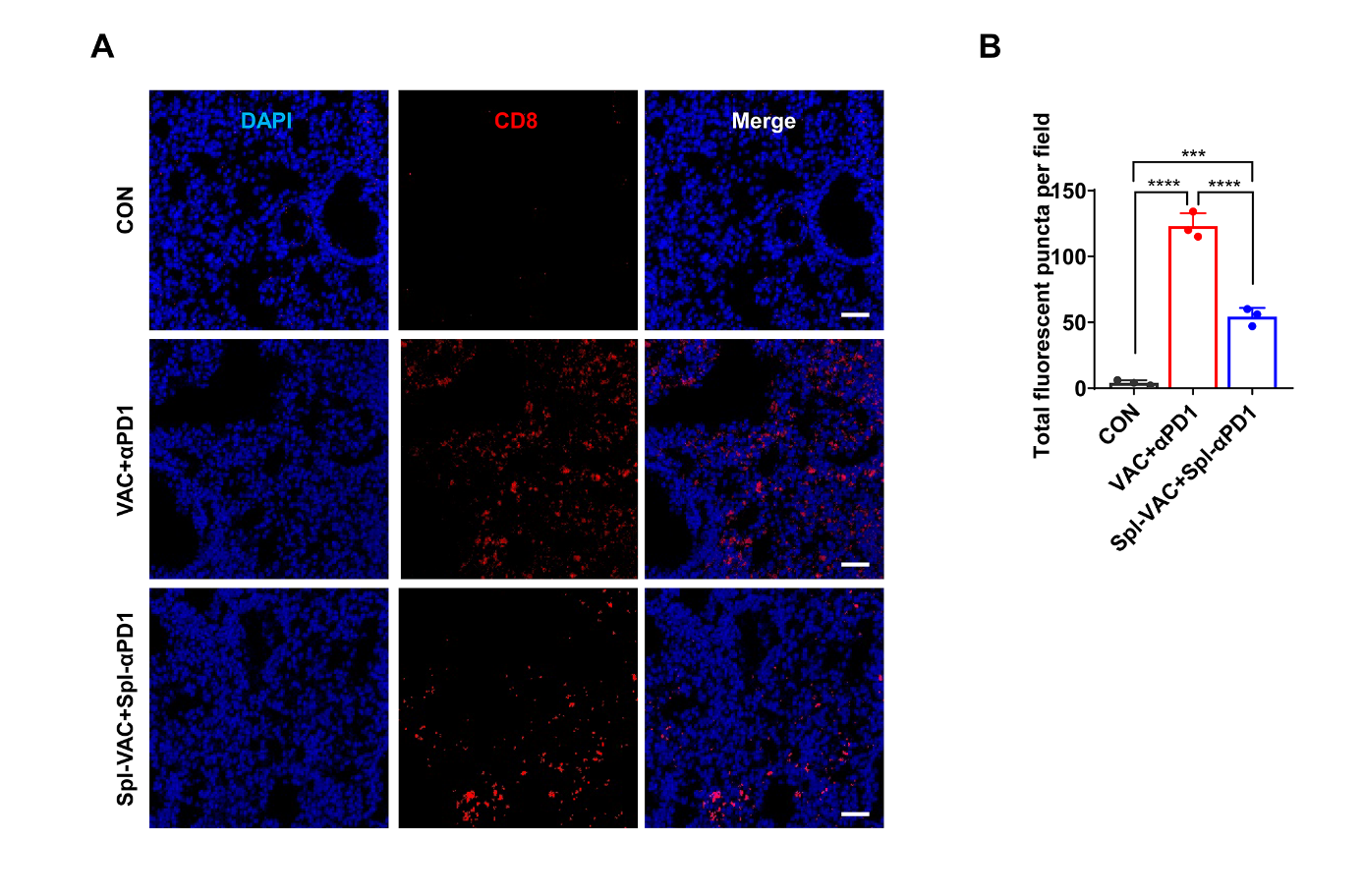


**Figure S32: Immunofluorescence Analysis of CD8^+^ T cell Infiltration in Lung Tissues in Spleen Targeting Experiment.** (A) Representative immunofluorescence images of CD8^+^ T cell infiltration in lung tissues of 4T1 tumor-bearing mice treated with PBS (control), tumor vaccine and αPD1 (VAC+αPD1) and Spl-VAC+Spl-αPD1. Scale bar, 50 μm. (B) Quantification of CD8^+^ T cell numbers in lung tissues across treatment groups (n = 3). Data are mean ± SD, ****p* < 0.001, *****p* < 0.0001.


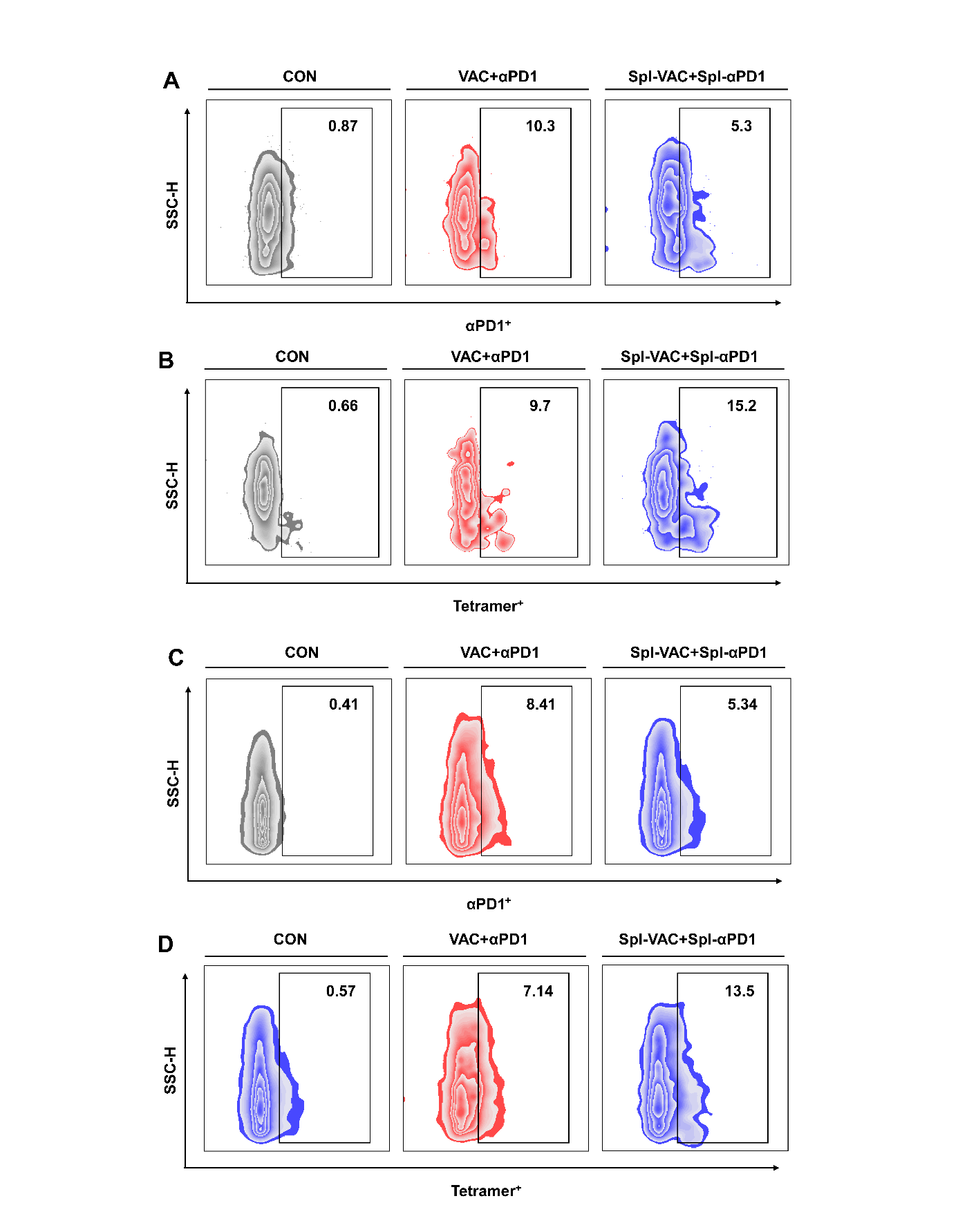


**Figure S33: Representative Flow Cytometry Plots for Assessing Spleen-Targeted Drug Delivery.** (A) PD1-blocked CD8^+^ T cells in lung tissues. (B) Tetramer^+^ CD8^+^ T cells in lung tissues. (C) PD1-blocked CD8^+^ T cells in spleen. (D) Tetramer^+^ CD8^+^ T cells in spleen.


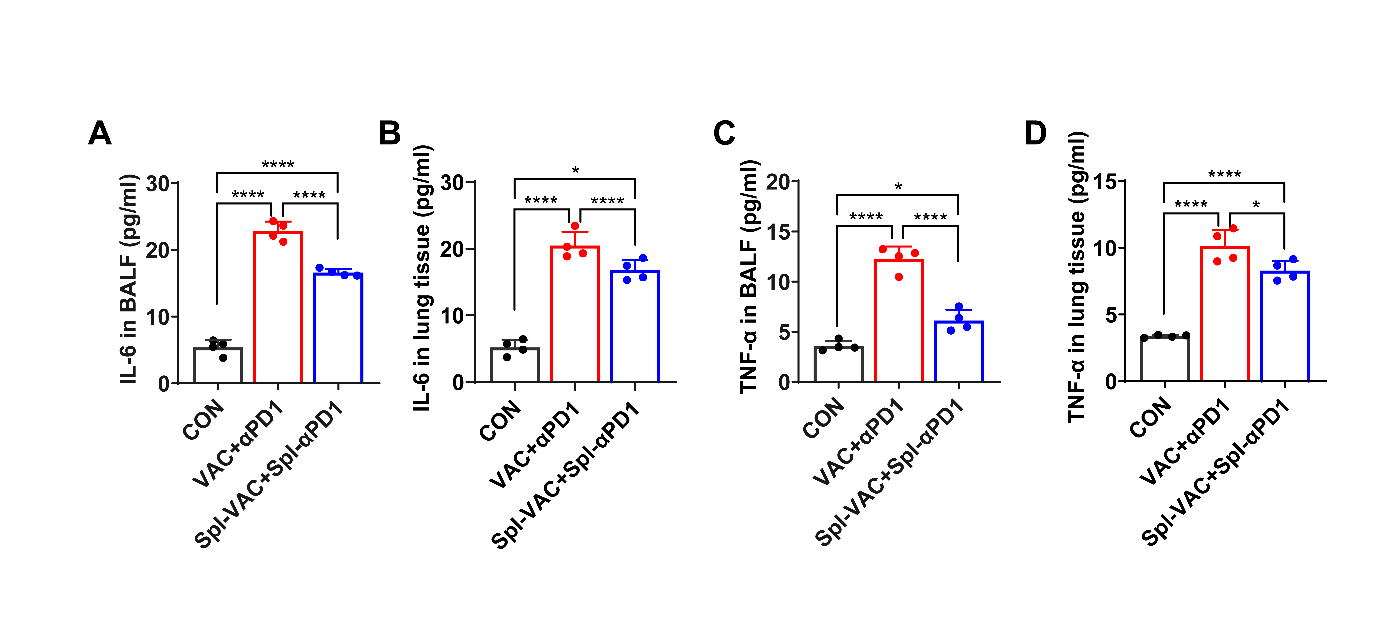


**Figure S34: Changes in Inflammatory Factors in Spleen Targeting Delivery Studies.** (A–D) ELISA quantification of (A) IL-6 in BALF, (B) IL-6 in lung tissue fluid, (C) TNF-α in BALF, and (D) TNF-α in lung tissue fluid (n = 4). Data are presented as mean ± SD, **p* < 0.05, ****p* < 0.001, *****p* < 0.0001.


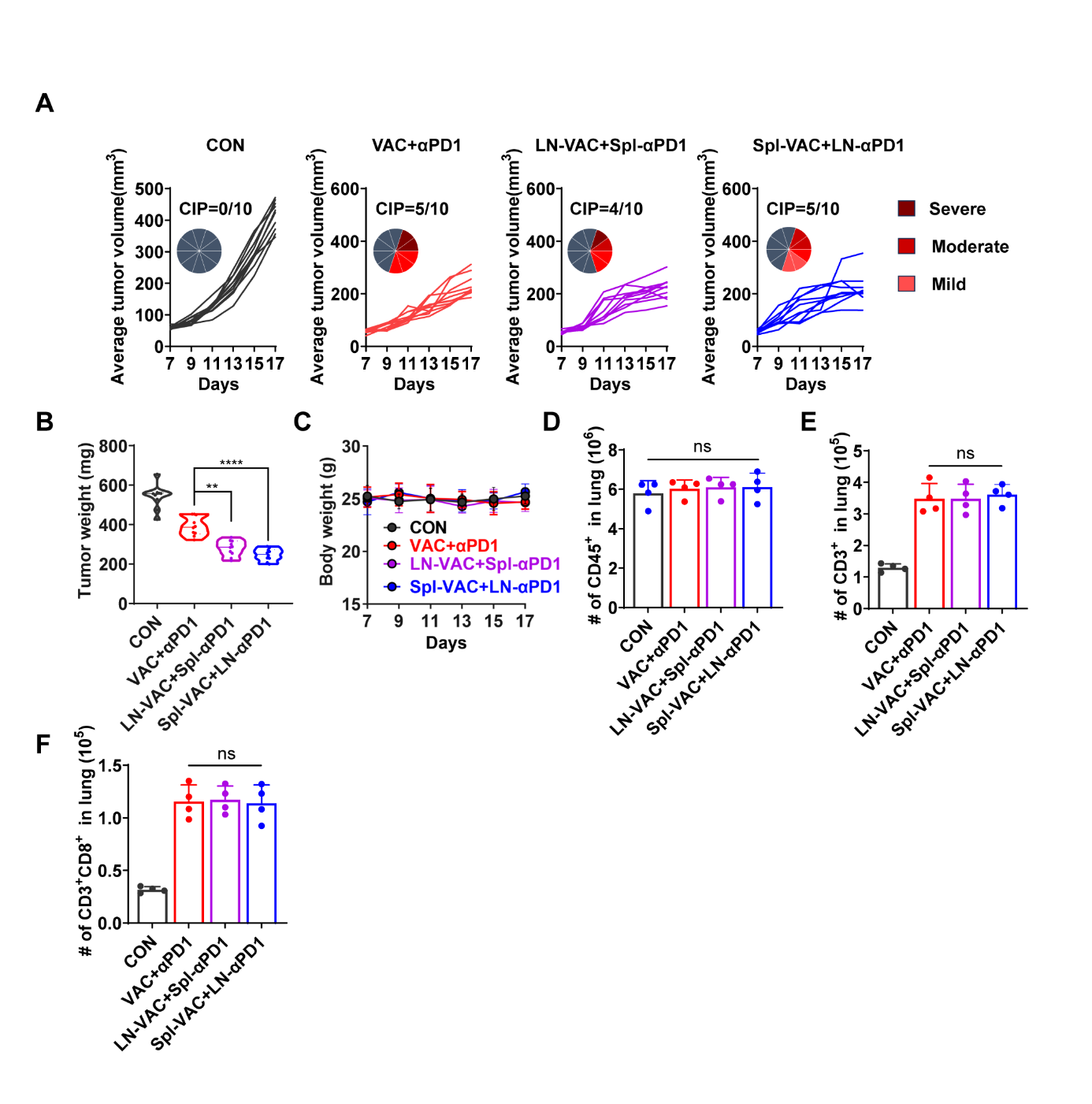


**Figure S35: Investigation of Differential Targeted Delivery Strategies.** (A) Individual tumor growth curves for 4T1 tumor-bearing mice in CON group, VAC+αPD1 group , LN-VAC+Spl-αPD1 group and Spl-VAC+LN-αPD1 group (n = 10)，the red portion of the pie chart represents the number of mice with CIP, with the intensity of the red shade indicating the severity of CIP. (B) Tumor weights at the study endpoint (n = 10). (C) Body weight changes across treatment groups (n = 10). (D–F) Flow cytometry analysis of (D) CD45^+^ cells and (E) CD3^+^ T cells (F) CD8^+^ T cells in lung tissues post-treatment (n = 4). Data are presented as mean ± SD, ***p* < 0.01, *****p* < 0.0001; ns, not significant.


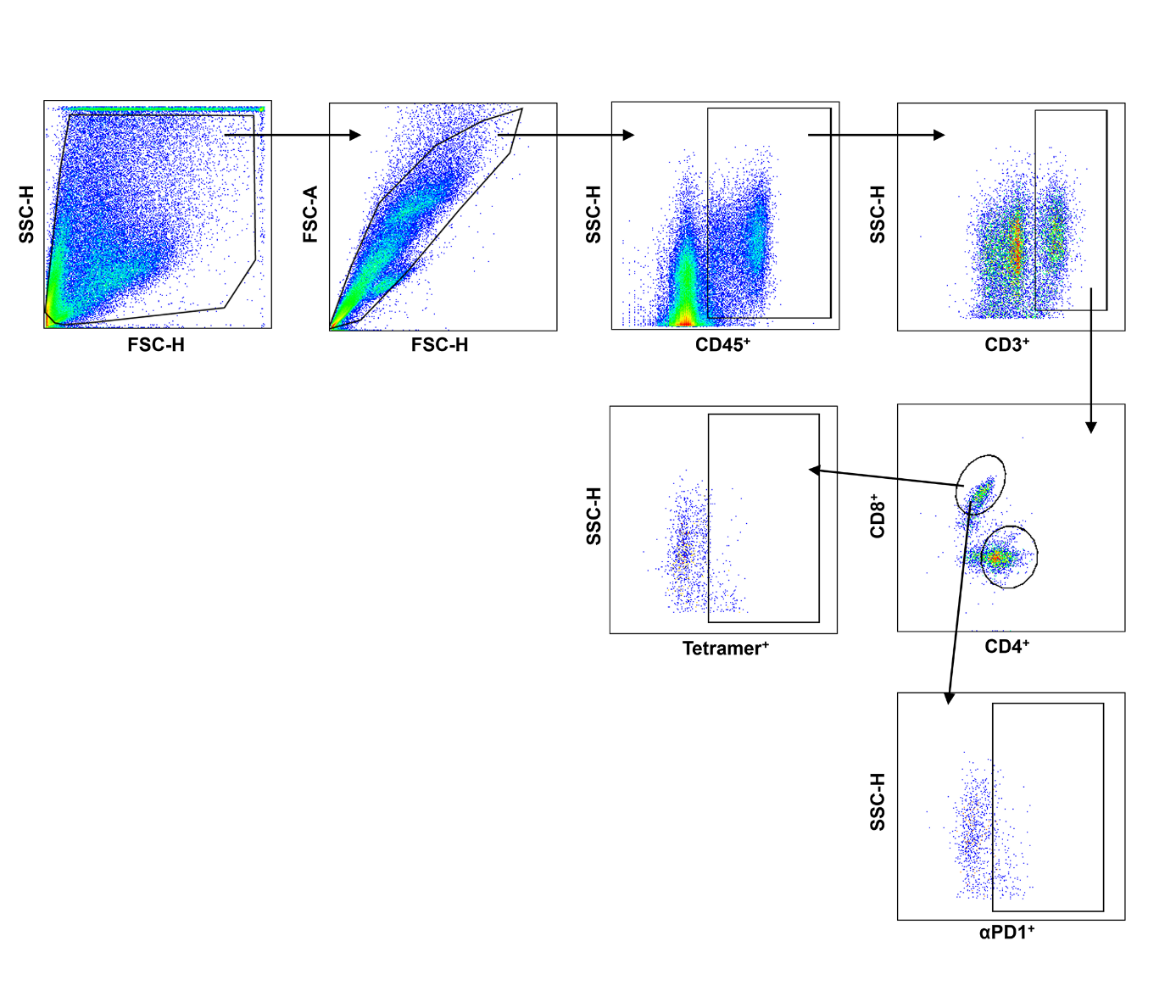
**Figure S36: Flow Cytometry Gating Strategy for Differential targeted Experiment.**
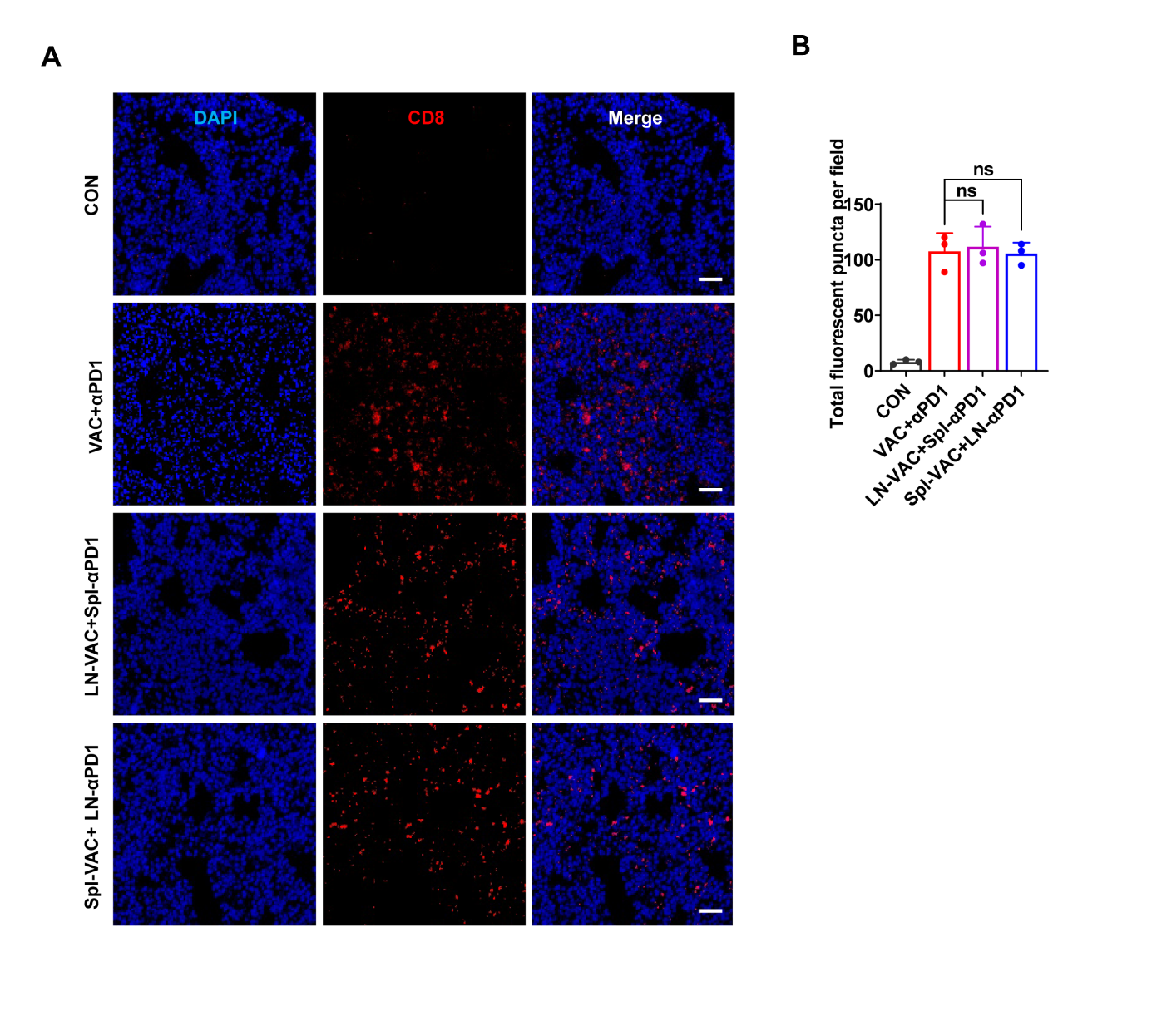


**Figure S37: Immunofluorescence Analysis of CD8^+^ T cell Infiltration in Lung Tissues in Differential Target Experiment.** (A) Representative immunofluorescence images of CD8^+^ T cell infiltration in lung tissues of 4T1 tumor-bearing mice treated with PBS (control), tumor vaccine and αPD1 (VAC+αPD1), LN-VAC+Spl-αPD1 and Spl-VAC+LN-αPD1. Scale bar, 50 μm. (B) Quantification of CD8^+^ T cell numbers in lung tissues across treatment groups (n = 3). Data are mean ± SD, ns, not significant.


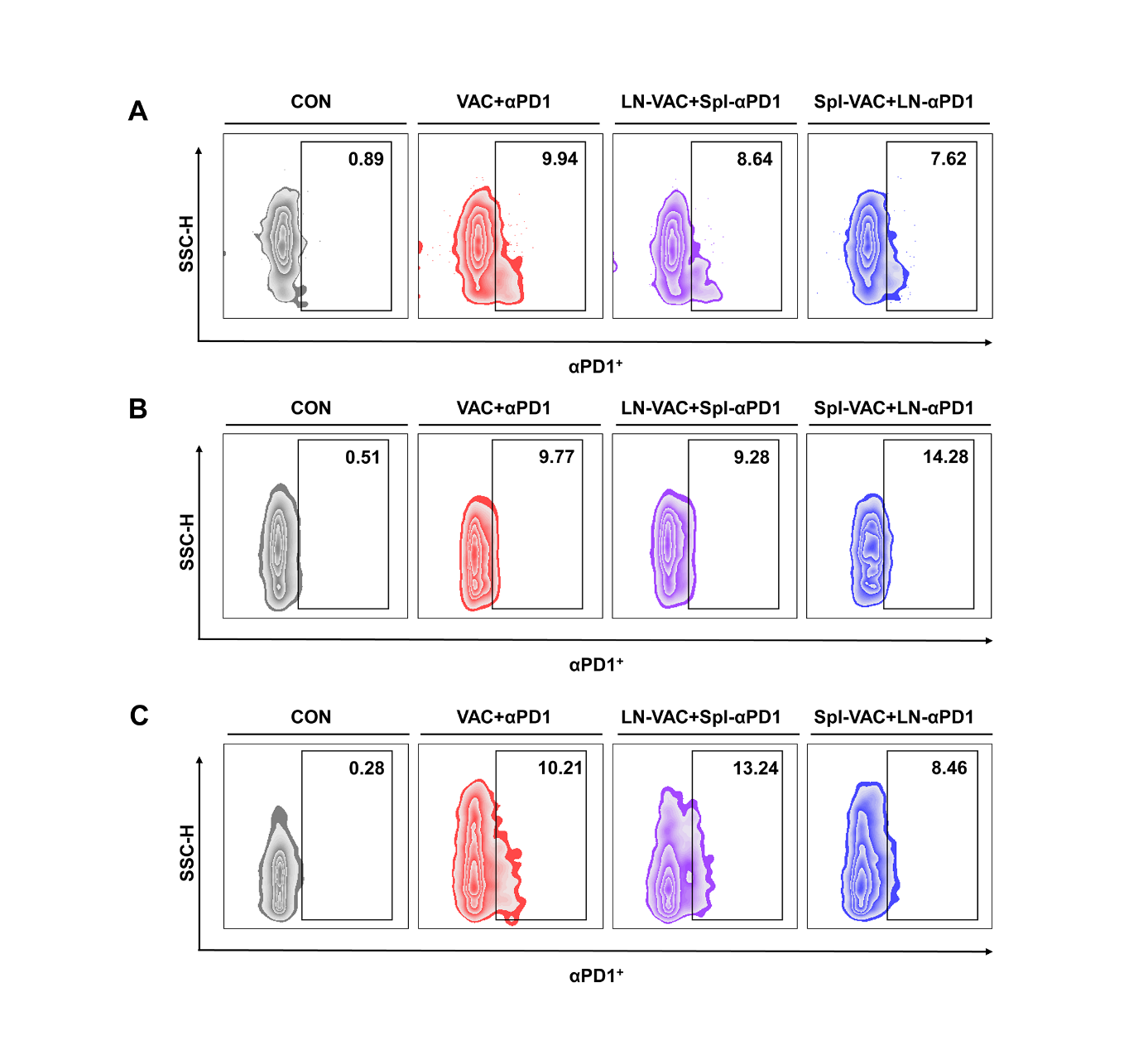


**Figure S38: Representative Flow Cytometry Plots for the Differential target Experiment.** PD1-blocked CD8^+^ T cells in (A) lung tissues,（B）lymph nodes and（C）spleen.


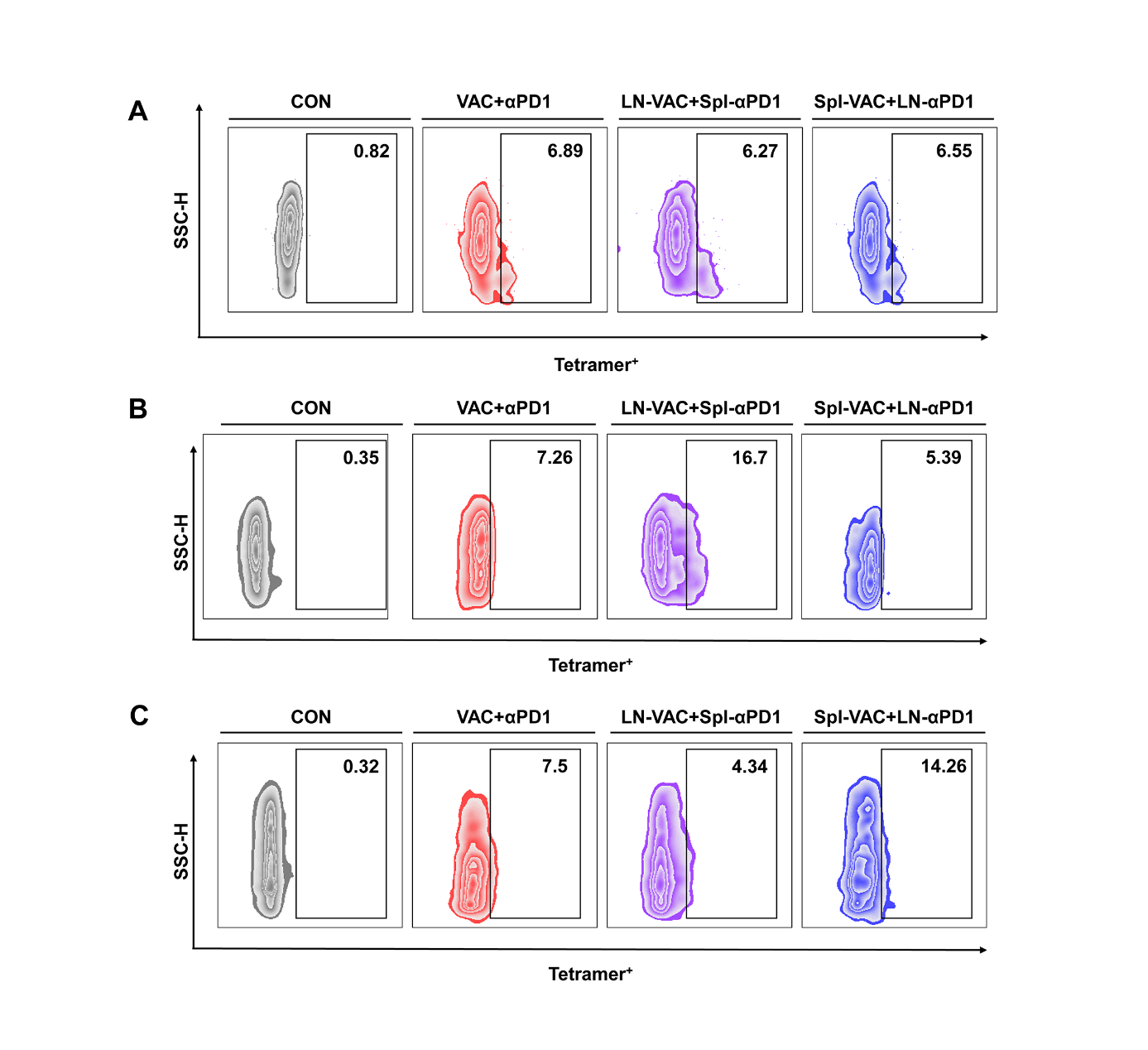


**Figure S****39: Representative Flow Cytometry Plots for the Differential target Experiment.** Tetramer^+^ CD8^+^ T Cell Populations in (A) lung tissue, (B) lymph nodes and (C) spleen.


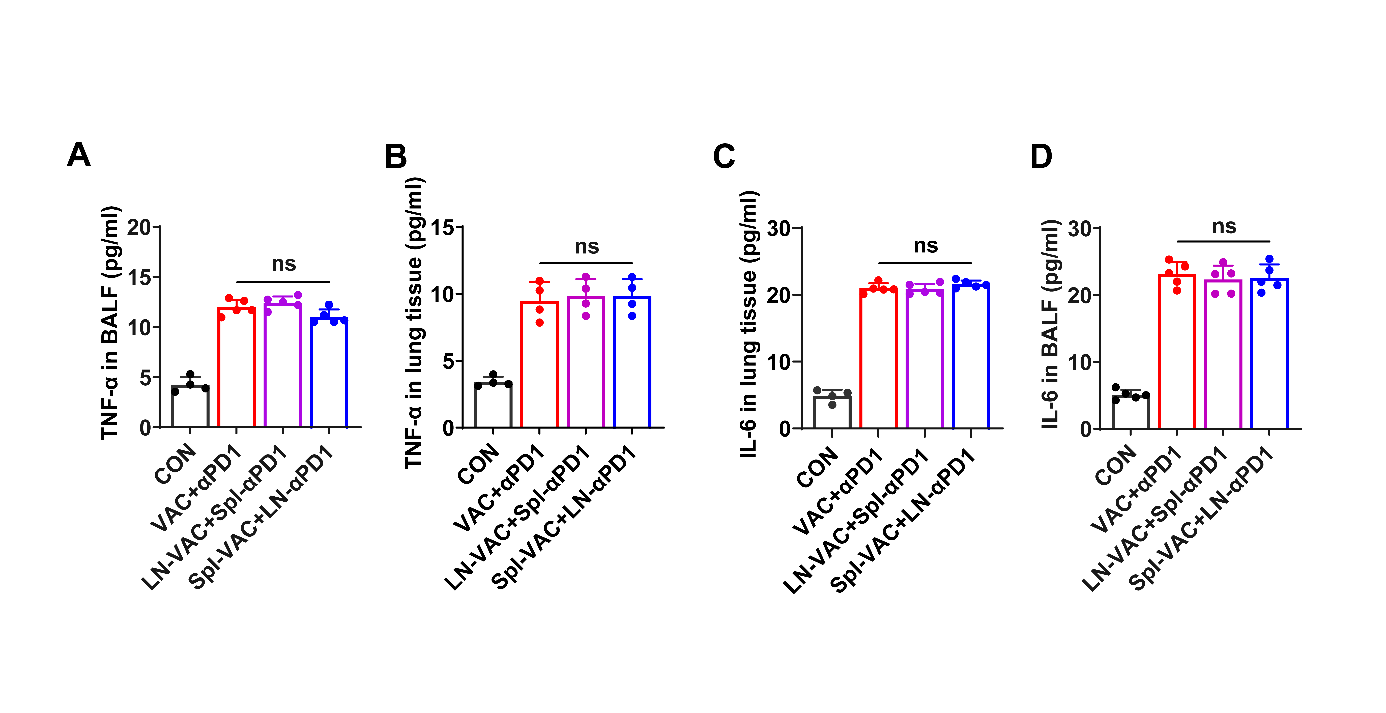


**Figure S40: Changes in Inflammatory Factors in Differential Targeting Delivery Studies.** (A–B) ELISA quantification of TNF-α in (A) BALF and (B) lung tissue fluid (n = 5). (C-D) ELISA quantification of IL-6 in (C) lung tissue fluid and (D) BALF (n = 5). Data are presented as mean ± SD, ns, not significant.


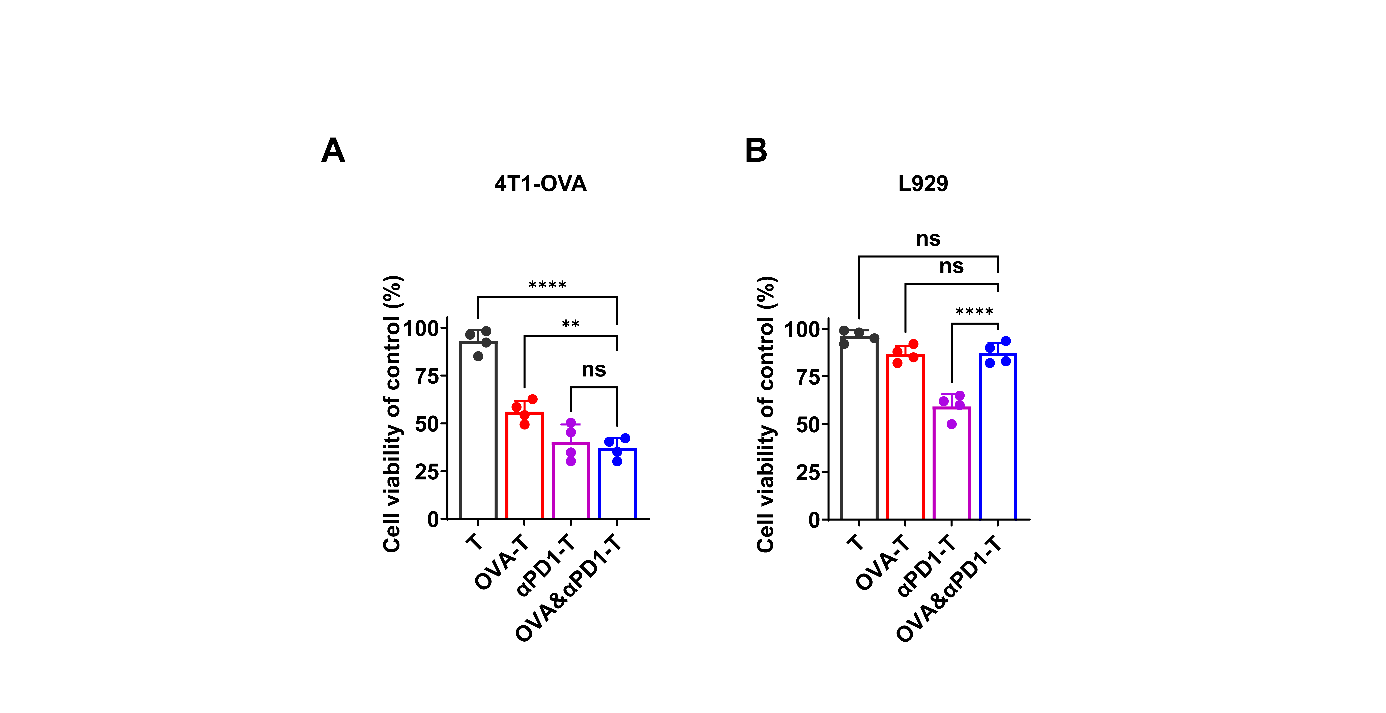


**Figure S41: *In Vitro* Cytotoxicity Assessment of T Cells.** (A–B) Cell viability of (A) 4T1-OVA cells and (B) L929 cells after co-incubation with T, OVA-T, PD1-T, or OVA&αPD1-T cells, assessed by MTT assay (n = 4). Data are presented as mean ± SD, ***p* < 0.01, *****p* < 0.0001，ns, not significant.


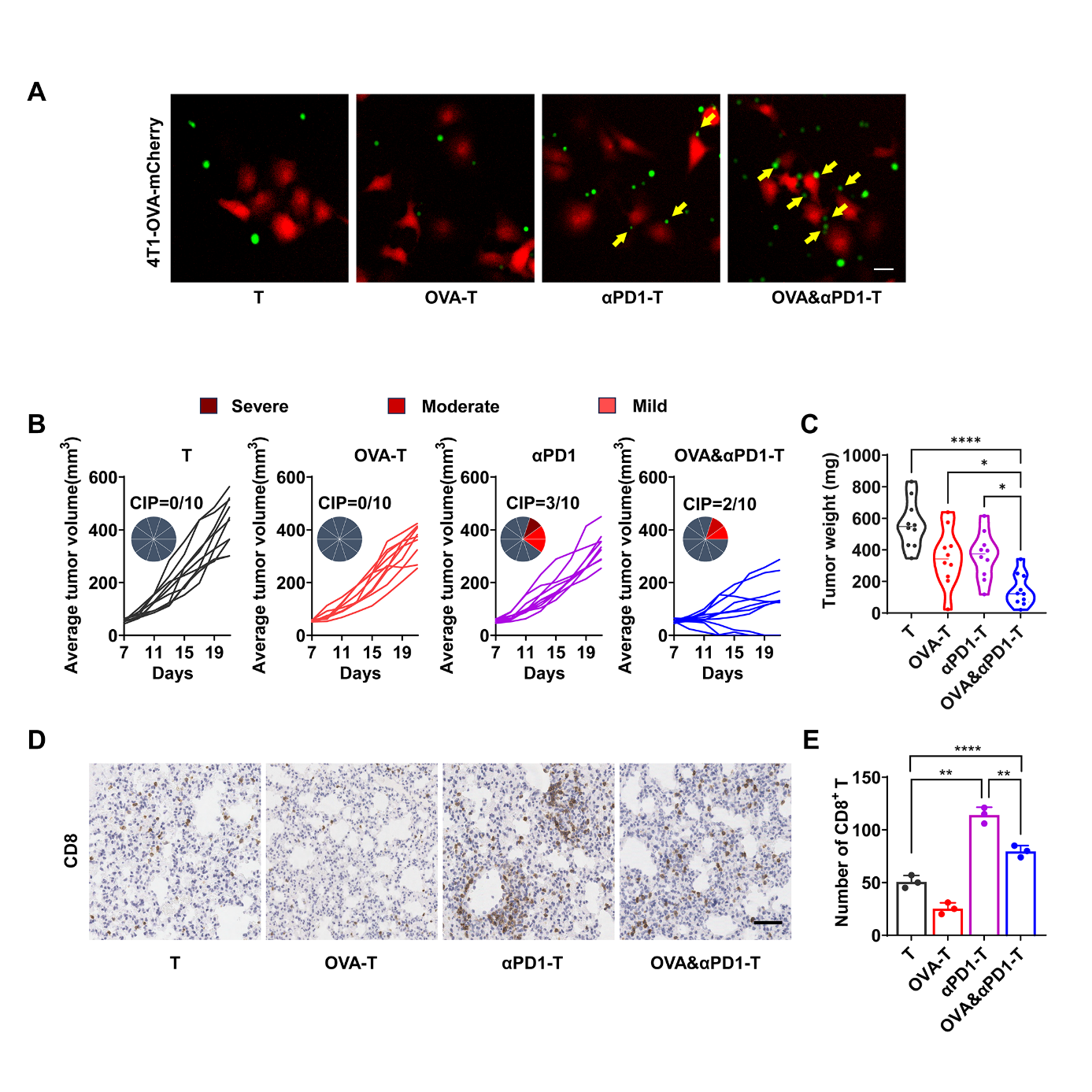


**Figure S42: Tumor-Specific T Cells with PD-1 Blockade Enhance Anti-Tumor Efficacy and Alter T Cell Distribution.** (A) IF analysis of CFSE-labeled T cells (green) co-incubated with 4T1-OVA-mCherry cells for 2 hours (n = 3). Scale bar, 50 μm. (B) Individual tumor growth curves for 4T1-OVA tumor-bearing mice in CON group, OVA-T group , αPD1-T group and OVA&αPD1 group (n = 10)，the red portion of the pie chart represents the number of mice with CIP, with the intensity of the red shade indicating the severity of CIP. (C) Tumor weights at the study endpoint (n = 10). (D) Immunohistochemical detection of CD8 expression in lung tissues. Scale bar, 100 μm. (E) Statistics of (D) (n = 3). Data are presented as mean ± SD, **p* < 0.05, ***p* < 0.01, *****p* < 0.0001.


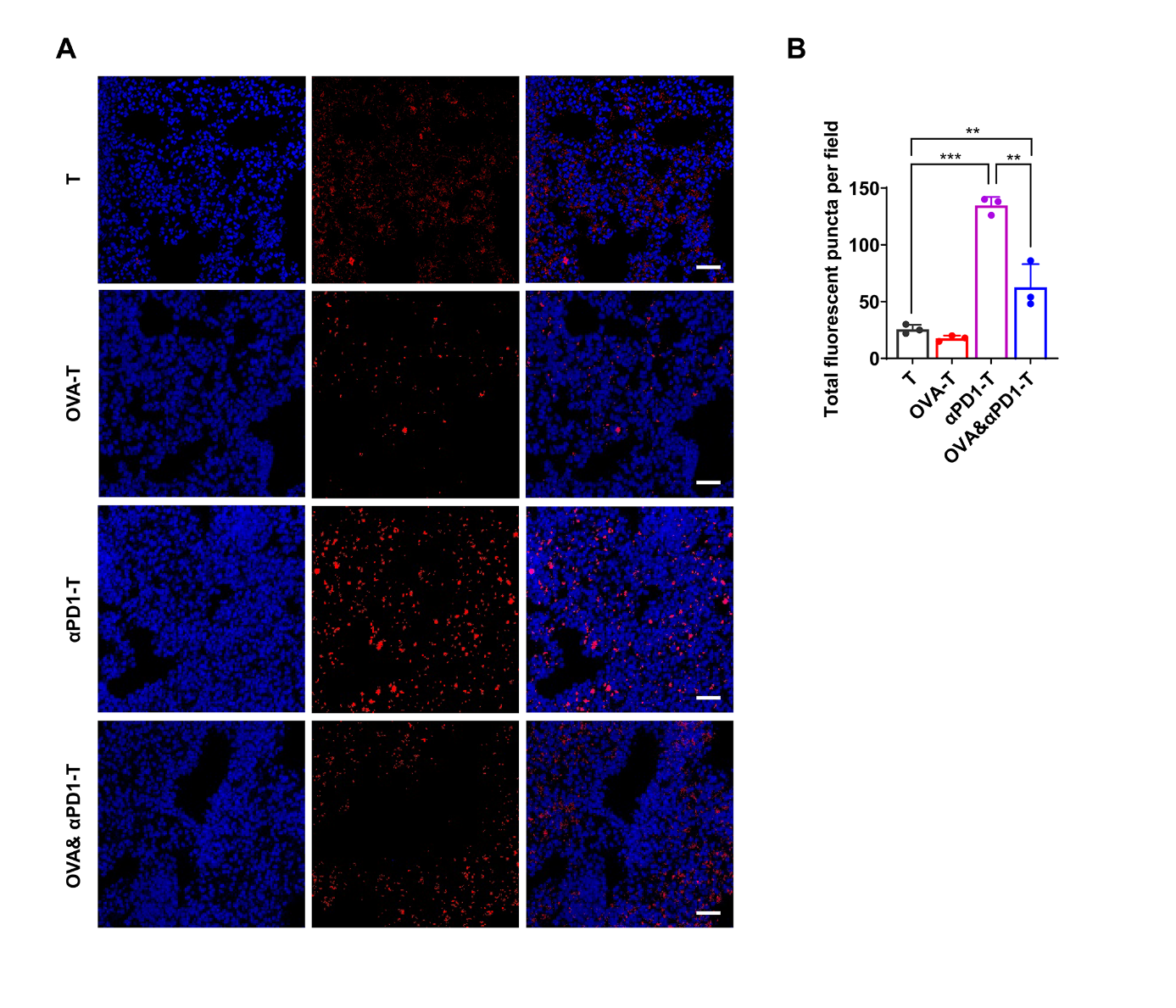


**Figure S43: Immunofluorescence Analysis of CD8^+^ T cell Infiltration in Lung Tissues of Adoptively Transferred Mice.**(A) Representative immunofluorescence images depicting CD8^+^ T cell infiltration in lung tissues of 4T1-OVA tumor-bearing mice treated with adoptive T cell therapy. The adoptively transferred T cells included control T cells, antigen-specific T cells (OVA-T), αPD1-blockade T cells (αPD1-T), and tumor-specific T cells with PD1-blockade. Scale bar, 50 μm. (B) Quantification of CD8^+^ T cell numbers in lung tissues across treatment groups (n = 3). Data are mean ± SD, ***p* < 0.01, ****p* < 0.001.


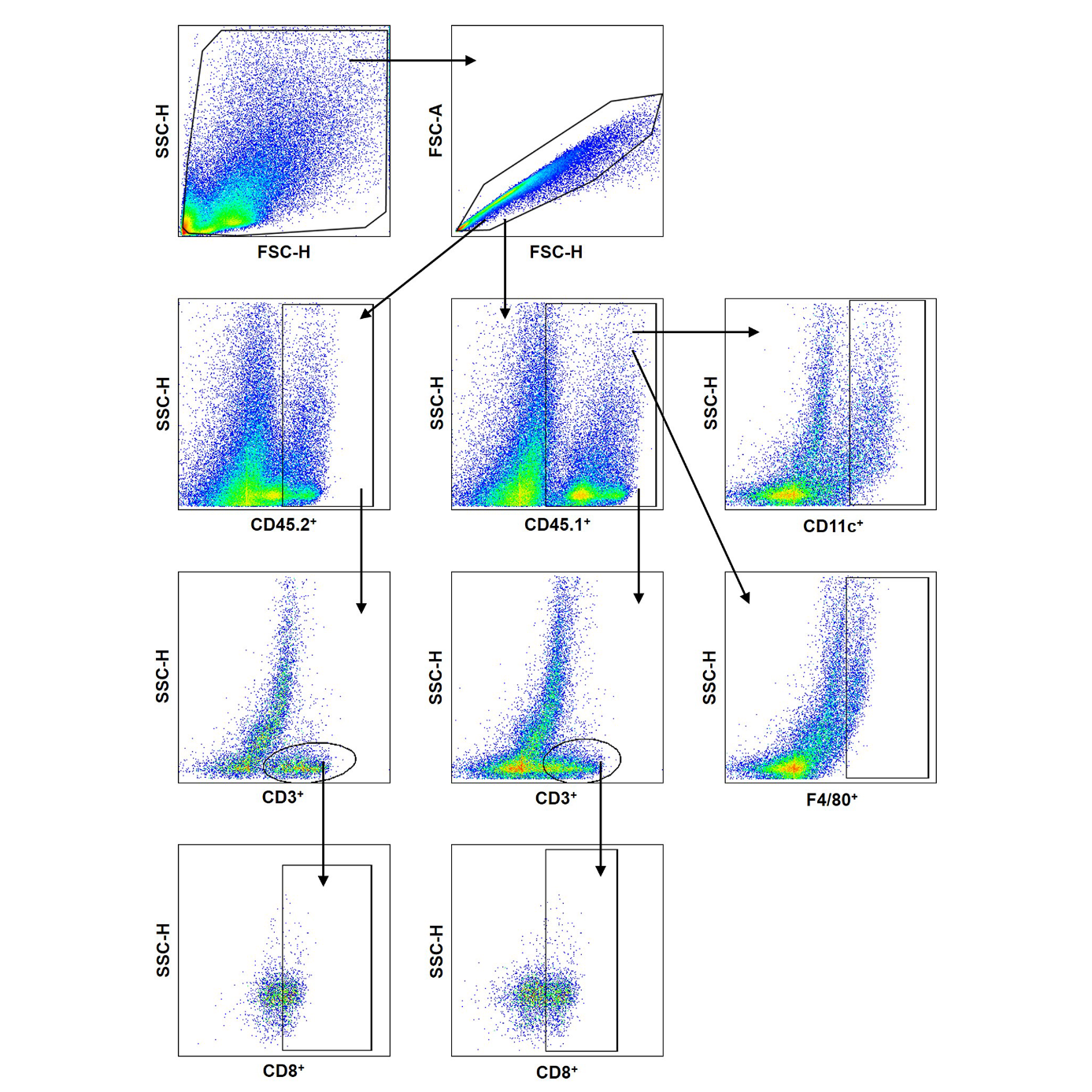


**Figure S44: Flow Cytometry Gating Strategy for Adoptively Transferred Cells in Mice.**


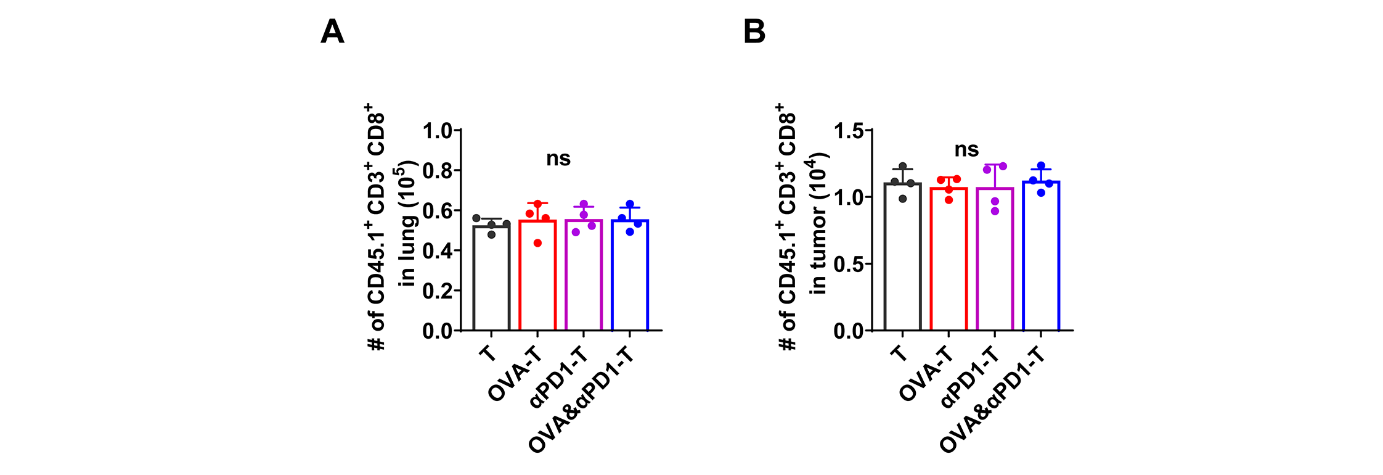


**Figure S45: Changes in CD8^+^ T Cell Populations in CD45.1 Mice.** (A) Alterations in CD8^+^ T cell populations within lung tissue (n = 3). (B) Alterations in CD8^+^ T cell populations within tumor tissue (n = 3). Data are presented as mean ± SD, ns, not significant.
